# A Fujiwara‐Moritani‐Type Alkenylation Using a Traceless Directing Group Strategy: A Rare Example of C−C Bond Formation towards the C2‐Carbon of Terminal Alkenes

**DOI:** 10.1002/ejoc.202201179

**Published:** 2023-01-26

**Authors:** Raheleh Pourkaveh, Maren Podewitz, Michael Schnürch

**Affiliations:** ^1^ Institute of Applied Synthetic Chemistry TU Wien Getreidemarkt 9/163-OC 1060 Vienna Austria; ^2^ Institute of Materials Chemistry TU Wien Getreidemarkt 9/165 1060 Vienna Austria

**Keywords:** benzoic acid, C−H activation, Fujiwara-Moritani, traceless directing group, unactivated alkene

## Abstract

Herein we report, a rhodium‐catalyzed Fujiwara‐Moritani‐type reaction of unactivated terminal alkenes and benzoic acid derivatives bearing electron donating residues under mild conditions. The acid functionality acts as a traceless directing group delivering products alkenylated in *meta*‐position to the electron donating substituent in contrast to the usually obtained *ortho*‐ and *para*‐substitution in Friedel‐Crafts‐type reactions. Remarkably, the new C−C bond is formed to the C2 of the terminal olefin, in contrast to similar reported transformations. Initially formed mixtures of *exo*‐ and *endo*‐double bond isomers can be efficiently isomerized to the more stable *endo*‐products.

## Graphical abstract

We describe the olefination in *ortho*‐position to a carboxylic acid group by employing simple olefins which do not carry electron‐withdrawing groups. The acid initially acts as the directing group but is subsequently removed by decarboxylation. The process introduces an alkene in *meta*‐position to an electron donating residue, which is complementary to classical electrophilic substitution reactions on aromatic systems.

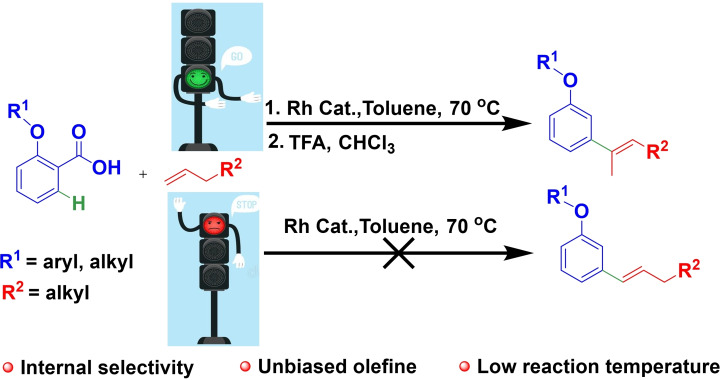

## Introduction

Developing synthetic methods for introducing olefin‐motifs into organic compounds is a topic of continuing importance in organic synthesis. Besides bulk chemicals, these frameworks are key units in natural products, biologically active molecules, and fine chemical products. Many different methods have been reported ranging from classical elimination reactions towards transition metal‐catalyzed approaches such as olefin metathesis,^[1]^ the Mizoroki‐Heck^[2]^ and the Fujiwara‐Moritani reaction.^[3]^ The latter method is an oxidative‐coupling approach which connects two C−H bonds, one of the olefin and one of the second coupling partner. This leads to selectivity issues commonly observed in direct C−H functionalization reactions due to the omnipresence of C−H bonds in organic molecules. This is usually approached by incorporating a directing group (DG) into the molecule.^[4]^ A potential drawback in this strategy is that additional steps for installing and removing of the DG might be required, in case the DG is not desired in the final product.

**Scheme 1:**
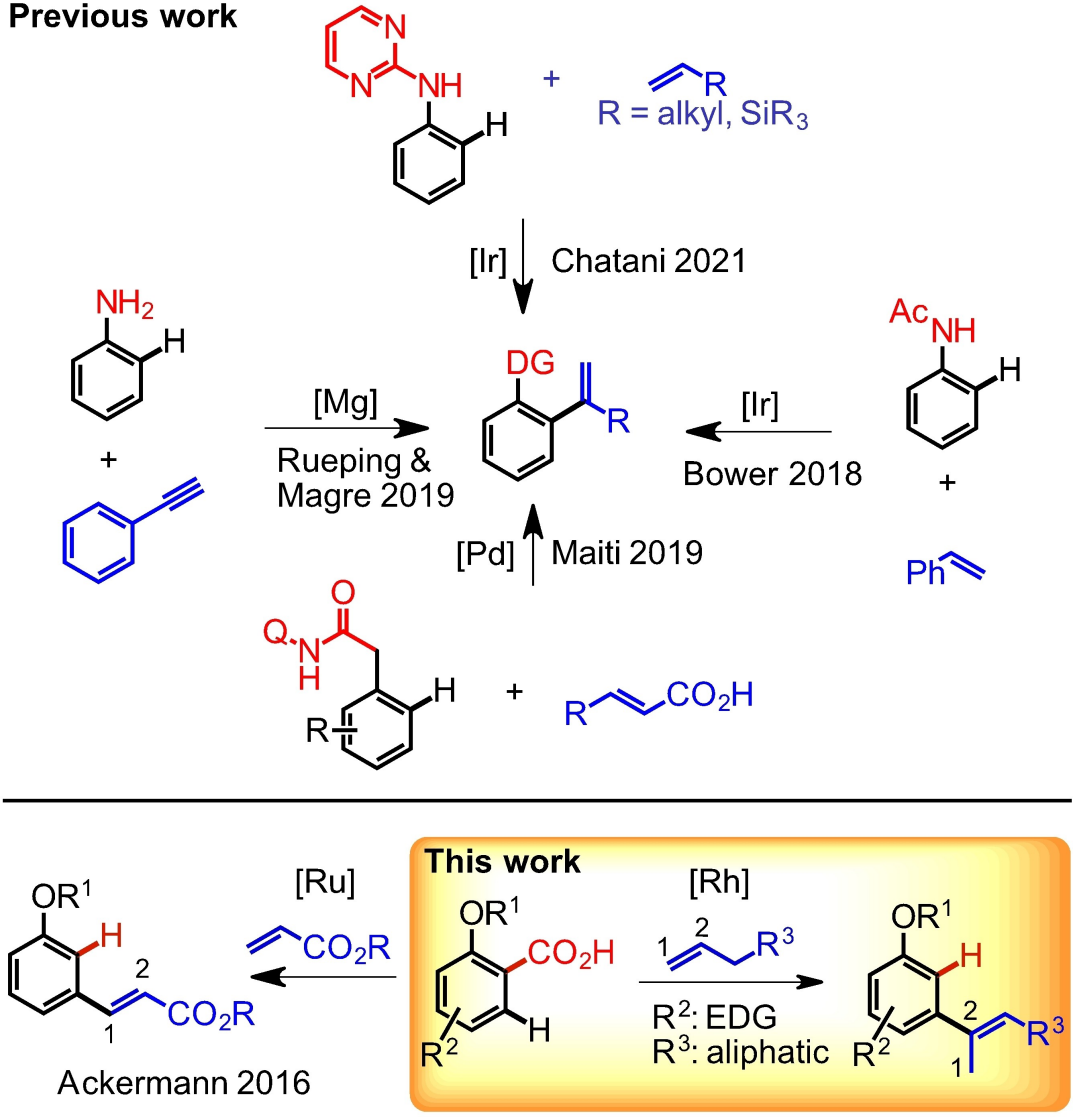
Different strategies for synthesis of substituted alkenes through decarboxylation.

Olefination reactions of aryl‐systems with terminal olefins have been widely explored in DG‐assisted C−H functionalization reactions. Usually, the new C−C bond is formed to the terminal carbon and most frequently acrylates (and other olefins carrying electron withdrawing groups) are the applied olefin coupling partners and unsubstituted, purely hydrocarbon olefins are typically neglected.^[5]^ These two limitations raised our interest and hence we set out to search for an olefination reaction with purely hydrocarbon olefins, in which the new C−C bond is formed to the C2 position of the olefin. Even though scarce, there is literature precedence for such transformations: the groups of Maiti,^[6]^ Bower,^[7]^ Chatani,^[8]^ as well as Rueping and Magre^[9]^ have reported protocols using phenylacetylenes, styrenes, cinnamic acid derivatives and alkyl or silyl substituted olefins as coupling partners using different DGs, which lead to isomeric mixtures of products. In case of the directed methods, olefination took place exclusively in *ortho* position to the DG (Scheme 1).

It was mentioned previously that installation and removal of a DG are potential drawbacks of this type of chemistry. Hence, so called traceless DGs have been developed, which carry out their *ortho*‐ directing effect first and are afterwards cleaved under the applied reaction conditions, a concept which was first introduced by Miura in 2008^[10]^ and immediately inspired further research.^[11]^ Besides *ortho* directing groups, also cleavable *meta*‐directing groups have been reported. For example, Zhou and co‐workers demonstrated the use of a silicon‐based traceless template which enables olefination of phenols at the *meta* site using Pd and Rh catalysts.^[12]^ Another variant for *meta* alkenylation of aromatic alcohols applying specifically designed U‐shaped nitrile templates has been reported by the group of Yu as well.^[13]^ However, these protocols were again only applied with activated olefins and in order to reach to the *meta*‐position, the directing group needs to have considerable size, so that it is frequently encountered that the molecular weight of the directing group surpasses the molecular weight of the product after removal of the DG. Additionally, synthesis of these DGs requires several synthetic steps.

In contrast, the carboxylic acid motif proved to be a convenient traceless DG since it is comparably small, many derivatives are commercially available often at low cost, and additionally, this concept can be applied for formal *meta*‐substitution, since an *ortho*‐substituted benzoic acid derivative delivers a 1,3‐disubstituted benzene derivative after the C−H functionalization‐decarboxylation sequence.^[14]^ In context of olefination reactions with terminal olefins the group of Ackerman reported in 2016 that acrylate‐based olefins can undergo alkenylation/decarboxylation reactions providing *meta*‐alkenylarenes where the new bond is formed to the C1 of the olefin (Scheme 1 bottom left),^[15]^ but no examples with simple olefins have been reported to the best of our knowledge. Hence, we set out to develop such a protocol.

## Results and Discussion

**Scheme 2:**
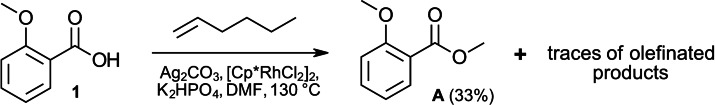
Unexpected esterification towards methyl 2‐methoxybenzoate A.

The initial screening studies were conducted using 2‐methoxybenzoic acid **1** as model substrate and 1‐hexene as olefin coupling partner to optimize diverse combinations of reaction temperature, solvent, base, oxidant, and catalyst (Table 1). The initial reaction conditions were inspired by a protocol of Jeganmohan,^[16]^ where carboxylic acid directed olefination towards a branched olefin took place, however without concomitant decarboxylation. As starting point, the reaction was carried out with [Cp*RhCl_2_]_2_ as catalyst, K_2_HPO_4_ as base, Ag_2_CO_3_ as oxidant in DMF as solvent at 130 °C (Entry 1). Under these conditions, methyl 2‐methoxybenzoate **A** formed as predominant product (33 %) besides trace amounts of several olefinated products (according to GC‐MS) (Scheme 2).

**Table 1:** Reaction optimization.^[a]^

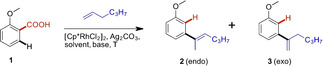					
Entry	Temp. [°C]	Solvent	Base	Yield^[b]^	**2: 3** ^[c]^
1^[d]^	130	DMF	K_2_HPO_4_	trace	2: 1
2^[d]^	130	dioxane	K_2_HPO_4_	15	2: 1
3^[d]^	130	toluene	K_2_HPO_4_	16	2: 1
4	70	toluene	K_2_HPO_4_	28	1: 2
5	70	toluene	DABCO	30	1: 2
6	70	toluene	Hexamine	27	1: 2.1
7	70	toluene	KOAc	25	1: 2.3
8	70	toluene	K_3_PO_4_	26	1: 1.9
9	70	toluene	KOH	24	1: 5
10	70	toluene	–	11	1: 1.9
11^[d]^	70	toluene	DABCO	24	1: 2
12^[e]^	70	toluene	DABCO	22	1: 2.2

[a] Reaction conditions: 2‐methoxybenzoic acid (0.2 mmol), *n*‐hexene (0.6 mmol), base (0.4 mmol), Ag_2_CO_3_ (20 mol%), [Cp*RhCl_2_]_2_ (5 mol%), solvent (1 mL). [b] Combined GC yields of branched (*exo* and *endo*) products (dodecane was used as an internal standard). [c] Determined by GC using dodecane as internal standard. [d] Methyl 2‐methoxybenzoate **A** was observed as dominant product. [e] Reaction was conducted under Ar atmosphere. [f] 2 equiv. Ag_2_CO_3_.

The esterification of substrate **1** was unexpected, but a literature survey revealed that DMF can also act as methylating agent under oxidative conditions and [Cp*RhCl_2_]_2_ as catalyst.^[17]^ Hence, it was replaced by other solvents. However, also in 1,4‐dioxane (9 %, Entry 2) and toluene (22 %, Entry 3) methyl 2‐methoxybenzoate was formed as by‐product, which indicates that DMF cannot be the sole methyl source. According to literature, methyl salicylate can act as a methylating agent for the esterification of carboxylic acids (see Scheme S1 in the Supporting Information for details) and trace amounts of a methyl ester present in **1** could initiate formation of **A** as well.^[18]^ Since this was not the aim of this study, no further investigations in this direction were undertaken.

The C2‐arylated olefin products formed were obtained as a mixture of *endo*‐ and *exo*‐double bond isomers **2** and **3** respectively. Amongst the other tested solvents (see Supporting Information for the complete list), toluene gave the best results and was selected for further optimization. Lowering the temperature to 70 °C gave an improved yield of 28 % (Entry 4), always as a mixture of isomers.

Next, a series of bases, both organic and inorganic, was investigated (Entries 5–9). Amongst the inorganic bases, it was noticed that potassium containing bases typically performed best (for a full list of tested bases see the Supporting Information). However, the highest conversion was observed with the organic bases DABCO (30 %, Entry 5) and hexan‐1‐amine (27 %, Entry 6). Since upon employing DABCO as base at 70 °C the formation of methyl 2‐methoxybenzoate **A** was suppressed, DABCO was selected as the base of choice for further investigations. In the absence of base, only 11 % of the product mixture were observed (Entry 9). Any other oxidant than Ag_2_CO_3_ did not perform well (see Supporting Information for the complete list), which is also true for other catalyst systems such as Pd(OAc)_2_, Pd(dba)_2_ + DPPF, Ni(cod)_2_, and Rh_2_(OOCCH_3_)_4_ which did not give any conversion. Conducting the reaction under argon atmosphere furnished a lower yield of desired product showing that presence of O_2_ has a beneficial effect on the reaction (Table 1, entry 11). Raising the amount of Ag_2_CO_3_ to 2 equiv. gave a lower yield (Entry 12).

Even though extensive optimization efforts were undertaken, the best conversion remained at a relatively low 30 % giving a mixture of isomers **2** **a** and **3** **a**. However, these two products could be isomerized using TFA in chloroform giving the *endo*‐products of type **2** exclusively in most cases (see substrate scope in Table 2).

**Table 2:**
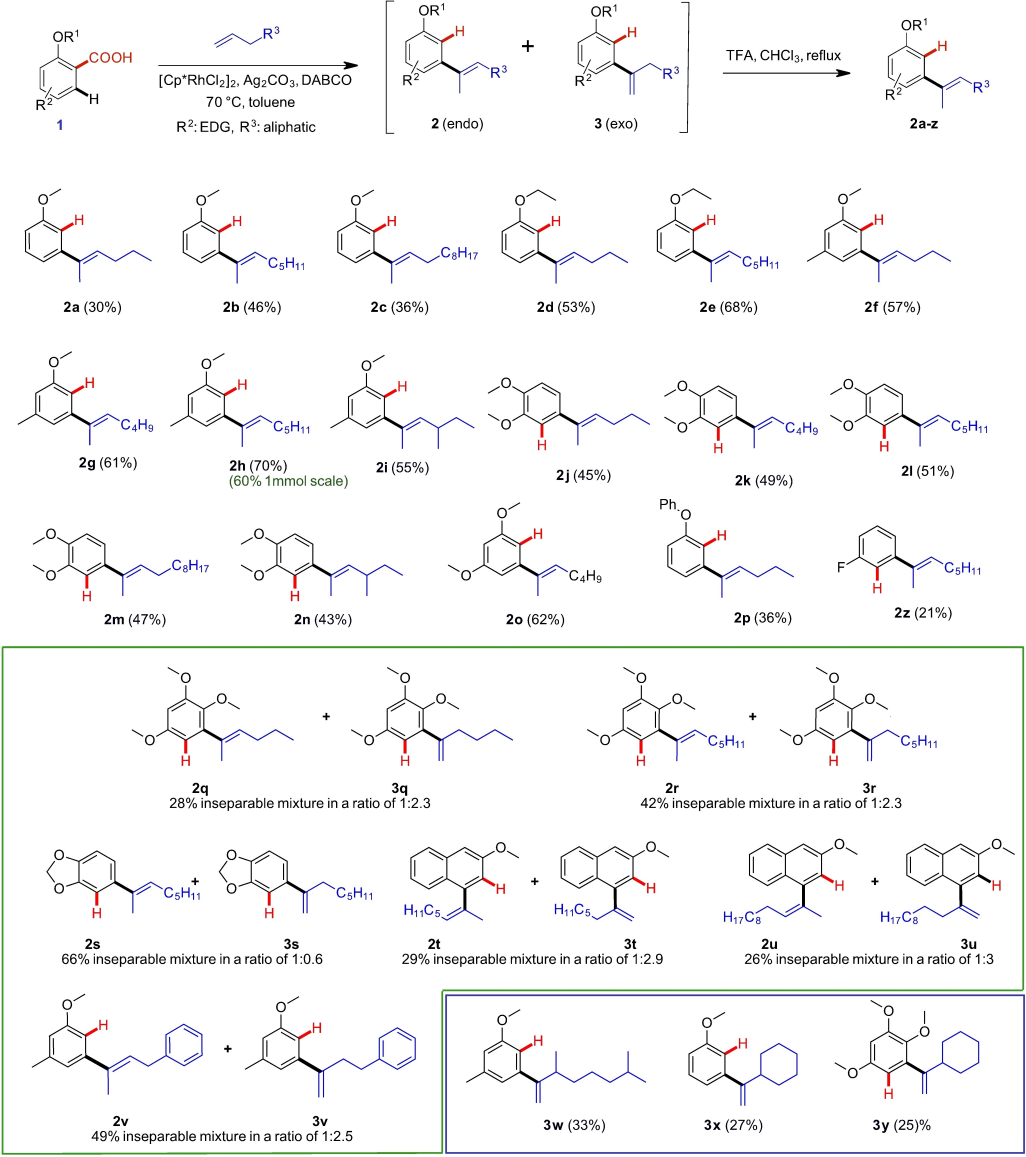
Substrate scope of *meta*‐olefination.^[a]^

[a] Reaction conditions: acid (0.2 mmol), olefin (0.6 mmol), DABCO (0.4 mmol), Ag_2_CO_3_ (0.04 mmol), [Cp*RhCl_2_]_2_ (5 mol%), toluene (1 mL) at 70 °C for 48 h. All reported yields are isolated yields. Ratio of *exo*/*endo* isomers obtained by H NMR after isolation.

Despite the low yield it was decided that due to the scarce examples for alkenylation at the C2 carbon a substrate scope evaluation is still justified, which turned out to be rewarding indeed, since it was found that the chosen test system was amongst the examples with the lowest yields. All results are reported in Table 2.

As mentioned, product **2** **a** of our initial test reaction could be obtained in 30 % isolated yield after isomerization. Using 1‐octene as olefin coupling partner, the yield could be improved and **2** **b** was obtained in 46 %. Even longer olefins undergo the transformation, however the yield was reduced again when 1‐dodecene was used (**2** **c**, 36 %). Interestingly, switching from a methoxy‐ to an ethoxy‐group in the substrate, the corresponding coupling products with 1‐hexene (**2** **d**) and 1‐octene (**2** **e**) were isolated in an improved 53 % and 68 % yields respectively. An additional electron donating methyl group in *para*‐position to the carboxylic acid moiety in the substrate had a beneficial effect on the yields as well, as can be seen in products **2** **f‐i**. Again, the reaction with 1‐octene towards **2** **h** was best performing giving 70 % yield, the highest of all investigated examples. This observation may be attributed to the higher boiling point of 1‐octene (121 °C) relative to 1‐hexene (63 °C).

In this series it was also demonstrated that the reaction is not strictly limited to linear terminal olefins, since 4‐methyl‐1‐hexene was also reactive giving the corresponding product **2** **i** in 55 % yield. An additional methoxy‐group *meta* to the COOH group of the substrate had a minor positive effect on the yield of products **2** **j**–**2** **n** always delivering the corresponding compounds ∼50 % isolated yield. An additional methoxy group in *para*‐position was more favorable giving **2** **o** in 62 % yield. Substituting the *ortho*‐alkoxy‐ for an *ortho*‐phenoxy group led to similar results and **2** **p** was obtained in 36 % (vs. 30 % for **2** **a**). Since additional EDGs showed a positive effect on isolated yields, we investigated 2,4,5‐trimethoxybenzoic acid as substrate as well with the hope to get high conversions and isolated yields. However, it turned out that this was not the case and additionally, the isomerization process was unsuccessful. Products **2** **q** and **3** **q** as well as **2** **r** and **3** **r** could only be isolated as an inseparable mixture of isomers in 28 % and 42 % yield respectively. The same is true for compound pairs **2** **s** and **3** **s**, **2** **t** and **3** **t**, **2** **u** and **3** **u**, as well as **2** **v** and **3** **v**. The reason for the inefficiency of the isomerization protocol remains puzzling, especially since product **2** **k** could be obtained in pure form after isomerization but the very similar product pair **2** **s** and **3** **s** proved to be resistant to isomerization. Finally, it was found that terminal olefins carrying a substituent at C3 (3,7‐dimethyloct‐1‐ene and vinylcyclohexane) also undergo the olefination process, but deliver solely the *endo* products **3** **w‐3** **y**, however with low yield and again resistant to isomerization, even though the thermodynamically favored tetra‐substituted double bond system would be formed. Besides alkoxy and phenoxy groups *ortho* to the COOH group, substrates carrying other electron donating substituents such as CH_3_, OH, NH_2_, NHMe, NMe_2_, and SH were tested as well, however without any conversion to an olefinated product (see Supporting Information for a complete list of investigated substrates, Scheme S2). Additionally, it was confirmed that the olefin needs to be terminal and mono‐substituted since neither cyclohexene and 2‐octene nor 3‐methyleneheptane underwent an olefination reaction. Furthermore, carboxylic acids substituted by electron withdrawing substituents were investigated as well. Only in the case of 2‐fluorobenzoic acid the reaction with 1‐octene delivered product **2** **z** in 21 % yield. The other substrates tested, namely 2‐chlorobenzoic acid, 2‐nitro benzoic acid, and 2‐methoxy‐5‐nitrobenzoic acid did not show any product formation.

We performed the olefination of 2‐methoxy‐4‐methylbenzoic acid with 1‐octene on a 1 mmol scale to prove the scalability of this method. The desired product **2** **h** was isolated in a yield of 60 %.

**Scheme 3:**
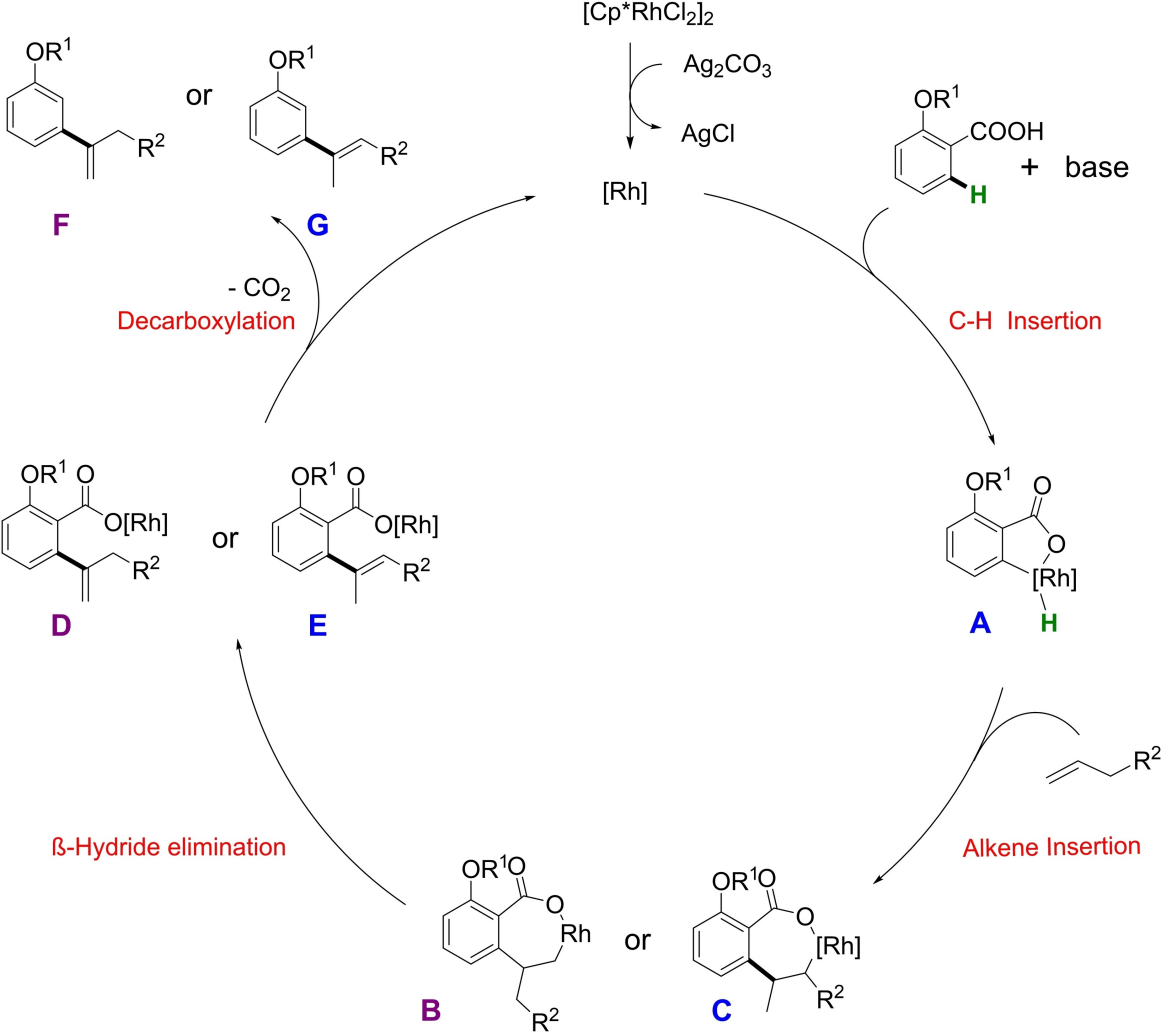
Proposed reaction mechanism.

Based on the known chemistry of Rh‐catalyzed regioselective C−H bond activation,^[19]^ metal‐mediated deciduous decarboxylation reactions,^[20]^ and own observations, a rational mechanism is proposed (Scheme 3).

Initially, the catalyst dissociates into a monomer species whereas Ag_2_CO_3_ could act as halide abstractor. However, the exact nature of the catalytically active species could not be identified. In the presence of base, the starting material will form a carboxylate anion, which brings the catalyst into proximity of the *ortho*‐C−H bond relative to the carboxylate. Through C−H insertion, a five‐membered rhodacycle **A** is formed, most likely through an electrophilic deprotonation pathway. Then, olefin insertion takes place into the Rh−C bond to create the seven‐membered rhodacycles **B** and **C**. β‐Hydride elimination from **B** will form the *exo*‐isomer **D**, whereas **C** will deliver the *endo*‐isomer **E**.^[21]^ A final decarboxylation step of **D** and **E** followed by protonation delivers the final products **F** and **G** and regenerates the active catalyst. When monitoring the reaction progress over time, it was observed that both product isomers **F** and **G** were formed at a constant ratio of 2 : 1 in favor of the *exo*‐isomer (See Scheme S3 in Supporting Information). This suggests that these two isomers are formed independently from each other and hence structures **B** and **C** are suggested as plausible intermediates.

It has to be mentioned that olefinated product still bearing the carboxylic acid moiety can be detected in small amount at the beginning of the reaction, however, the amount decreases over time. This is an indication that olefination occurs prior to the decarboxylation step. Furthermore, initial calculations revealed that the observed C2 substituted products is less stable than the C1, hence, it potentially has to be formed through a thermodynamically less favored transition state (see Supporting Information), which leads to the conclusion that the present transformation is under kinetic control. This goes in line with our observation during reaction optimization, where at 130 °C the product from C1 substitution of the olefin was observed. To elucidate the whole mechanistic cycle, in depth studies are required, including DFT‐calculations and ideally isolation of intermediates of the catalytic cycle. However, this is beyond the scope of this study.

## Conclusion

In summary, we have successfully developed a decarboxylative oxidative C−H alkenylation reaction which affords exclusively *meta*‐substituted electron rich arenes in preparatively useful yields. Benzoic acid derivatives reacted smoothly with simple alkenes under mild reaction conditions. Our method does not require activated olefins (e.g., acrylate derivatives), in contrast to most literature contributions and is one of the rare cases where the new C−C bond is formed to the C2 of the olefin. Furthermore, isomerization of the initially obtained mixture of double bond isomers can be achieved in many cases to deliver the thermodynamically more stable double bond, another feature of this reaction unprecedented so far. Only in cases where the C3 of the terminal olefin is substituted, this isomerization does not occur and exclusively the *exo* double bond isomers of type **3** are obtained. Mechanistic investigations are currently on the way.

## Experimental Section

Chemicals were purchased from commercial suppliers and used without further purification. All reactions were magnetically stirred and heated in a metallic reaction block. Purification was accomplished using preparative thin layer chromatography on 20×20 cm^2^ silica gel plates (layer thickness 1,000 μm). Removal of high‐boiling solvent (toluene) was carried out by azeotropic distillation (adding 69 wt% of methanol). NMR‐spectra were recorded in CDCl_3_ on an Avance III HD 600 (600 MHz) or Bruker Avance UltraShield 400 spectrometer, and chemical shifts (δ) are reported in ppm and are referenced to the solvent peak. For CDCl_3_, proton NMR spectra were referenced to 7.26 ppm and carbon NMR spectra to 77.16 ppm. Coupling constants (J) are given in Hertz (Hz). Multiplicities of the signals are abbreviated as follows: s=singlet, d=doublet, t=triplet, q=quartet, m=multiplet, dd=doublet of doublet, dt=doublet of triplet, td=triplet of doublet, ddd=doublet of doublet of doublet and bs=broad singlet. Carbon NMR were recorded either as APT, DEPTQC or standard decoupled C^13^ spectra. GC–MS runs were performed on a Thermo Finnigan Focus GC / DSQ II using a standard capillary column BGB 5 (30 m x 0.32 mm ID). GC spectra were recorded on a Thermo Focus GC using a BGB‐5 capillary column (30 m×0.32 mm, 1.0 μm film, achiral) with the following oven temperature program: Start at 100 °C (hold 2 min), 35 °C/min, 300 °C (hold 4 min). GC yields were calculated by using the response factor of the corresponding compound relative to dodecane as internal standard, which was determined by calibration. HR‐MS for literature unknown compounds were carried out by I. JelenkoviC‐Didic at TU Wien, Institute for Chemical Technologies and Analytics; all samples were analyzed by LC‐IT‐TOF‐MS in only positive ion detection mode with the recording of MS and MS/MS spectra. All samples were filtered through PALL Acrodisc CR 13 mm syringe filters with 0.2 μm PTFE membrane prior to GC analysis.

**General procedure A for the rhodium‐catalyzed olefination reaction**: Benzoic acid (0.2 mmol, 1 equiv.), DABCO (44 mg, 0.4 mmol, 2 equiv.), Ag_2_CO_3_ (11 mg, 0.04 mmol, 0.2 equiv.), [Cp*RhCl_2_]_2_ (6 mg, 0.01 mmol, 0.05 equiv.) and alkene (0.6 mmol, 3 equiv.) were weighed into an 8‐mL glass vial. Dry toluene (1.0 mL) was added to the reaction mixture. The reaction mixture was stirred at 70 °C for 48 h. The progress of the reaction was monitored by GCMS. The crude residue was purified by preparative thin layer chromatography on 20×20 cm^2^ silica gel plates (layer thickness 1000 μm), PE:EtOAc (10 : 1).

**General procedure B for acid‐catalyzed isomerization reaction**: Mixtures of isomers obtained in the olefination reaction were dissolved in CHCl_3_ (0.1 M) and trifluoro acetic acid (58 μL) was added. Subsequently, the reaction mixture was refluxed until complete isomerization occurred (∼6 h) (or no more progress was detected) according to GCMS. After cooling to room temperature, the solvent was evaporated. The residue was dissolved in dichloromethane/ether and filtered off on a short celite pad. Finally, the solvent was evaporated to give a colorless oil.

**General procedure C for isomerization reaction**: Mixtures of isomers obtained in the olefination reaction were dissolved in 0.5 mL CDCl3 and kept in that solvent without stirring for 5 days. Complete isomerization occurred which could be determined directly via ^1^H‐ and ^13^C NMR spectroscopy. After that, the solvent was removed by evaporation and pure *endo* product obtained.

## Conflict of interest

The authors declare no conflict of interest.

1

## Supporting information

As a service to our authors and readers, this journal provides supporting information supplied by the authors. Such materials are peer reviewed and may be re‐organized for online delivery, but are not copy‐edited or typeset. Technical support issues arising from supporting information (other than missing files) should be addressed to the authors.

Supporting Information

## Data Availability

The data that support the findings of this study are available in the supplementary material of this article.
