# Supplementary material for: A Fujiwara‐Moritani‐Type Alkenylation Using a Traceless Directing Group Strategy: A Rare Example of C−C Bond Formation towards the C2‐Carbon of Terminal Alkenes
Source: European J Org Chem. 2023 Jan 26;26(8):e202201179. doi: 10.1002/ejoc.202201179 (PMC10946834; doi:10.1002/ejoc.202201179)

European Journal of Organic Chemistry

Supporting Information

A Fujiwara-Moritani-Type Alkenylation Using a Traceless Directing Group Strategy: A Rare Example of C–C Bond Formation towards the C2-Carbon of Terminal Alkenes

Raheleh Pourkaveh, Maren Podewitz, and Michael Schnürch*

Table of Contents

Complete Table of further Optimization S2

Scheme S1: Potential pathway for the formation of methyl 2-methoxybenzoate (compound A)........ S3

Scheme S2: Additional substrates tested S3

Scheme S3: Time screening of the reaction between n-hexene and 2-methoxybenzoic acid............. S4

DFT Calculations........ S5

Analytical data of products S6

NMR Spectra........S20

Complete Table of further Optimization

Table S1: Screening of conditions for branched meta alkenylation[a]

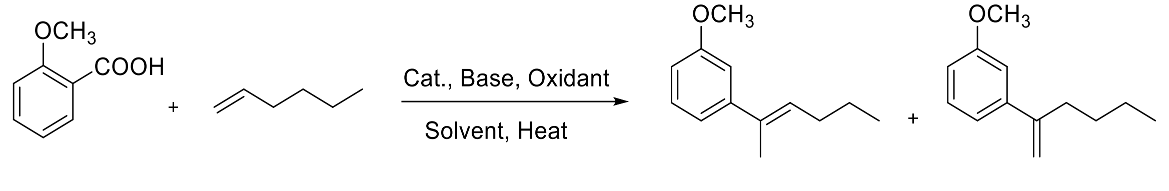

Entry	Temp. (°C)	Solvent	Oxidant	Base	Cat. (mol%)	Yield (%)[b]	2:3[c]
1	110	Toluene	Ag_2CO_3	K_2HPO_4	$[Cp^*RhCl_2]_2$	20	2:1
2	90	Toluene	Ag_2CO_3	K_2HPO_4	$[Cp^*RhCl_2]_2$	23	1.5:1
3	50	Toluene	Ag_2CO_3	K_2HPO_4	$[Cp^*RhCl_2]_2$	14	1:2.2
4	RT	Toluene	Ag_2CO_3	K_2HPO_4	$[Cp^*RhCl_2]_2$	10	1:2.3
5	70	DMSO	Ag_2CO_3	K_2HPO_4	$[Cp^*RhCl_2]_2$	trace	0:1
6	70	CH_3CN	Ag_2CO_3	K_2HPO_4	$[Cp^*RhCl_2]_2$	8	1:1.6
7	70	HFIP	Ag_2CO_3	K_2HPO_4	$[Cp^*RhCl_2]_2$	trace	0:1
8	70	NMP	Ag_2CO_3	K_2HPO_4	$[Cp^*RhCl_2]_2$	trace	1.2:1
9	70	EtOH	Ag_2CO_3	K_2HPO_4	$[Cp^*RhCl_2]_2$	10	1:1.7
10	70	1,4-Dioxane	Ag_2CO_3	K_2HPO_4	$[Cp^*RhCl_2]_2$	23	1:2
11	70	o-Xylene	Ag_2CO_3	K_2HPO_4	$[Cp^*RhCl_2]_2$	25	1:2
12	70	Toluene	Ag_2CO_3	Et_3N	$[Cp^*RhCl_2]_2$	-	-
13	70	Toluene	Ag_2CO_3	Guanidin carbonate	$[Cp^*RhCl_2]_2$	15	1:2.7
14	70	Toluene	Ag_2CO_3	DTBP	$[Cp^*RhCl_2]_2$	10	1:1.5
15	70	Toluene	Ag_2CO_3	K_2CO_3	$[Cp^*RhCl_2]_2$	-	-
16	70	Toluene	Ag_2CO_3	Cs_2CO_3	$[Cp^*RhCl_2]_2$	10	1:2
17	70	Toluene	Ag_2CO_3	LiCl	$[Cp^*RhCl_2]_2$	7	0:1
18	70	Toluene	Ag_2CO_3	LiOAc	$[Cp^*RhCl_2]_2$	8	1:2.5
19	70	Toluene	AgOAc	DABCO	$[Cp^*RhCl_2]_2$	12	2.6:1
20	70	Toluene	Ag_2O	DABCO	$[Cp^*RhCl_2]_2$	10	1.6:1
21	70	Toluene	AgOTf	DABCO	$[Cp^*RhCl_2]_2$	trace	1.5:1
22	70	Toluene	$Cu(OAc)_2$	DABCO	$[Cp^*RhCl_2]_2$	-	-
23	70	Toluene	BQ	DABCO	$[Cp^*RhCl_2]_2$	-	-
24	70	Toluene	O_2	DABCO	$[Cp^*RhCl_2]_2$	-	-
25	70	Toluene	Ag_2CO_3	DABCO	$Pd(OAc)_2$	-	-
26	70	Toluene	Ag_2CO_3	DABCO	$Pd(dba)_2$ + DPPF	-	-
27	70	Toluene	Ag_2CO_3	DABCO	$Ni(cod)_2$	-	-
28	70	Toluene	Ag_2CO_3	DABCO	$Rh_2(OOCCH_3)_4$	-	-
29	70	Toluene	Ag_2CO_3	DABCO	$[Cp^*RhCl_2]_2$+Neocuproine	29	1:3

[a]reaction conditions: 2-methoxybenzoic acid (0.2 mmol), *n*-hexene (0.6 mmol), base (0.4 mmol), oxidant (20 mol%), catalyst (5mol%), solvent (1mL).
[b]GC yields of branched (both exo and endo) products (dodecane was used as an internal standard).
[c]exo/endo isomeric ratio determined by GC.

Scheme S1: Potential pathway for the formation of methyl 2-methoxybenzoate (compound A)

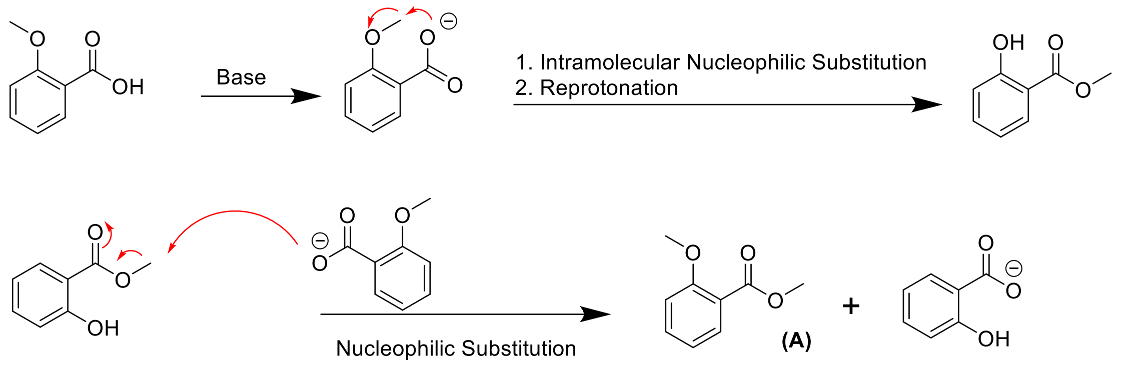

Initially, in the presence of base, a carboxylate anion is formed. After that, intramolecular nucleophilic substitution (and eventually reprotonation) leads to the formation of methyl salicylate which can act as a methylating agent as previously described in literature,[1] now in our case for an intermolecular nucleophilic substitution, generating compound **A**.

Scheme S2: Additional substrates tested

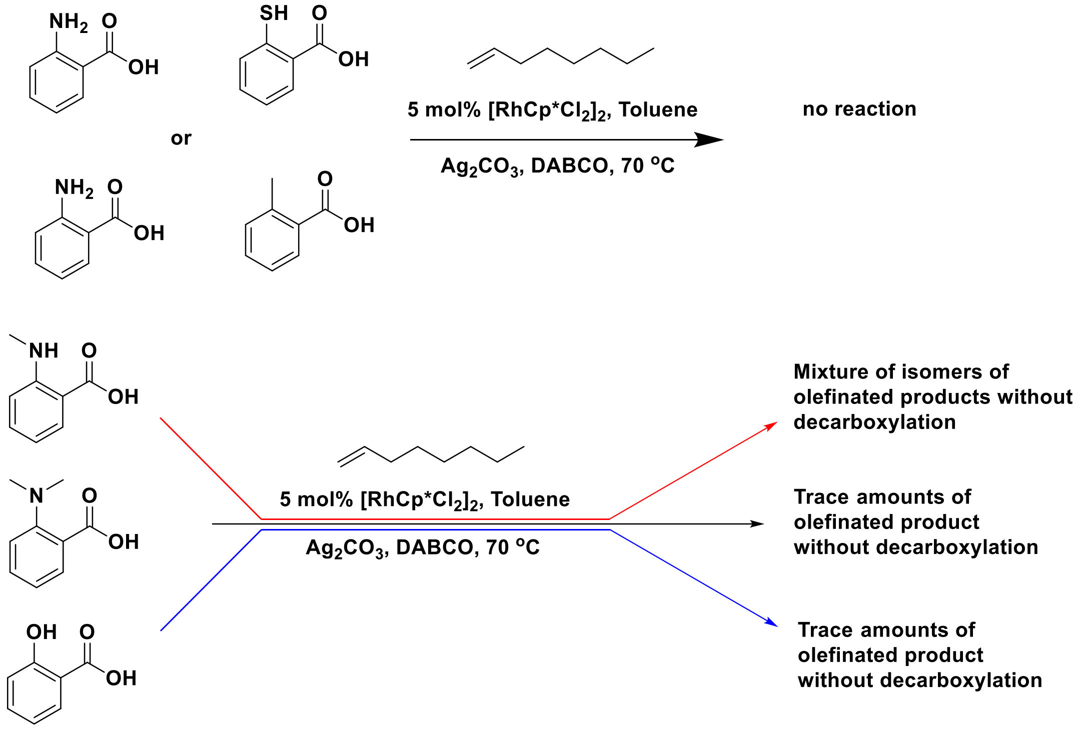

[1] Chen, S.; Jia, L.; Li, X.; Luo, M. Methyl Salicylate as a Selective Methylation Agent for the Esterification of Carboxylic Acids. *Synthesis.* **2014,** *46,* 263-268.

Scheme S3: Time screening of the reaction between n-hexene and 2-methoxybenzoic acid

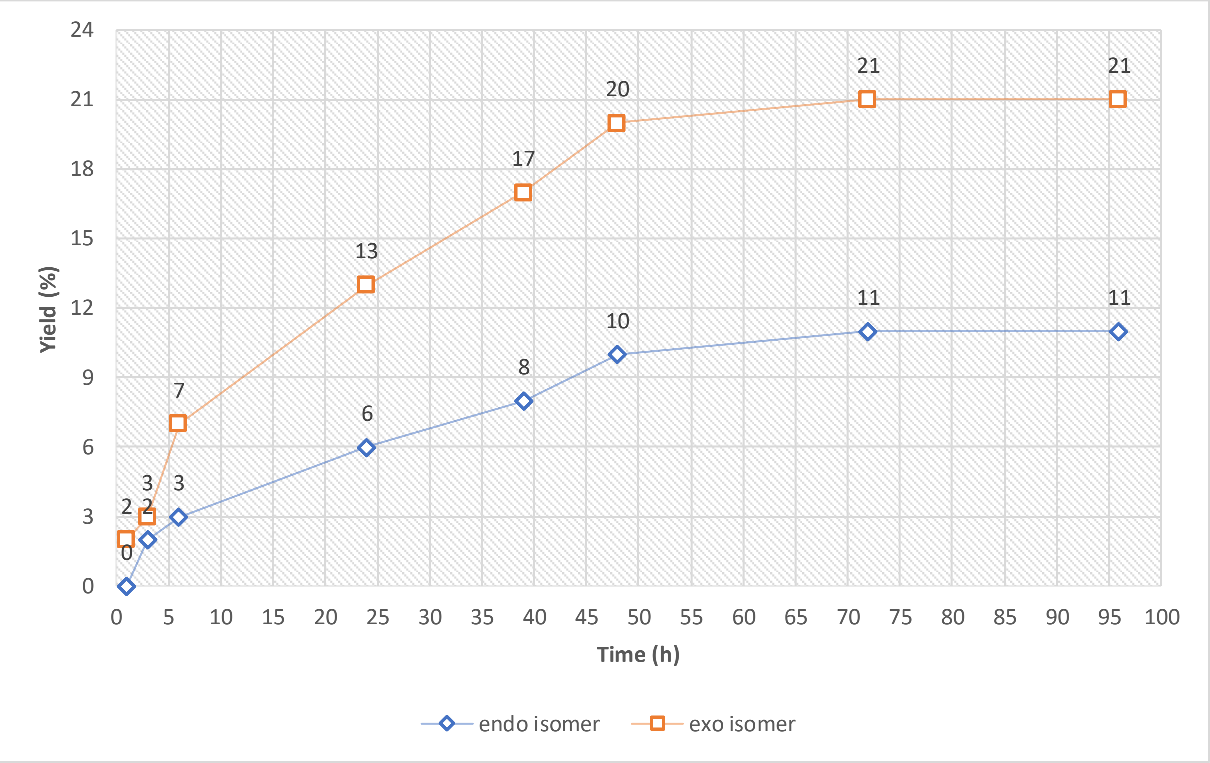

DFT Calculations

To further elucidate the nature of the two intermediates **B** and **C**, we performed density functional theory (DFT) calculations in toluene modelled as implicit solvent. Initial structures were optimized with BP86[2]/def2-SVP[3]/D3[4] and final single point energies were obtained with ωB97xd[5]/def2-TZVP[6]/toluene in both cases toluene was modelled using the Conductor-Like Screening Model (COSMO)[7] as implemented in Turbomole.[8] To identify the most stable conformer the Conformer-Rotamer-Ensemble Tool (CREST)[9] was used and the obtained conformers were reoptimized with DFT (see above). All calculations were performed in Turbomole, structures were visualized with PyMol.[10]

Our calculations revealed that both intermediates are stable and their geometries in line with chemical intuition (compare Scheme 3 in manuscript and Figure S1). The C2 substituted product **B** is found to be less stable by 10.2 kJ/mol than the C1 product **C**. Consequently, we surmise the formation of **B** is kinetically controlled and has therefore a lower lying transition state than the formation of **C**.

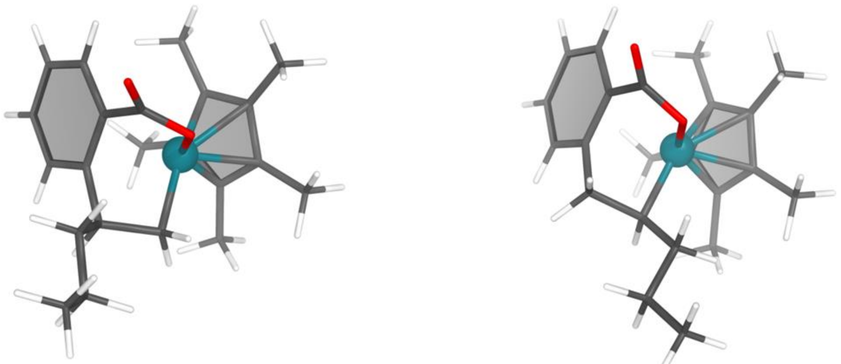

B C

Figure S1: Optimized structures of the C2 product (**B**) and the C1 product (**C**).

---

[2] A. D. Becke, *Phys. Rev. A*, **1988**, *38*, 3098-3100. J. P. Perdew, *Phys. Rev. B*, **1986**, *33*, 8822-8824.
[3] F. Weigend and R. Ahlrichs, *Phys. Chem. Chem. Phys.*, **2005**, *7*, 3297-3305.
[4] S. Grimme, S. Ehrlich and L. Goerigk, *J. Comput. Chem.*, **2011**, *32*, 1456-1465.
[5] J.-D. Chai and M. Head-Gordon, *Phys. Chem. Chem. Phys.*, **2008**, *10*, 6615-6620.
[6] F. Weigend and R. Ahlrichs, *Phys. Chem. Chem. Phys.*, **2005**, *7*, 3297-3305.
[7] A. Schäfer, A. Klamt, D. Sattel, J. C. W. Lohrenz and F. Eckert, *Phys. Chem. Chem. Phys.*, **2000**, *2*, 2187-2193. A. Klamt and G. Schürmann, *J. Chem. Soc. Perkin Trans. 2* **1993**, 799– 805.
[8] S. G. Balasubramani, G. P. Chen, S. Coriani, M. Diedenhofen, M. S. Frank, Y. J. Franzke, F. Furche, R. Grotjahn, M. E. Harding, C. Hättig, A. Hellweg, B. Helmich-Paris, C. Holzer, U. Huniar, M. Kaupp, A. M. Khah, S. K. Khani, T. Müller, F. Mack, B. D. Nguyen, S. M. Parker, E. Perlt, D. Rappoport, K. Reiter, S. Roy, M. Rückert, G. Schmitz, M. Sierka, E. Tapavicza, D. P. Tew, C. v. Wüllen, V. K. Voora, F. Weigend, A. Wodyński and J. M. Yu, *J. Chem. Phys.*, 2020, **152**, 184107. TURBOMOLE V7.5 2021, a development of University of Karlsruhe and Forschungszentrum Karlsruhe GmbH, 1989-2007, TURBOMOLE GmbH, since 2007; available from http://www.turbomole.com.
[9] P. Pracht, F. Bohle, S. Grimme. *Phys. Chem. Chem. Phys.* **2020**, *22*, 7169-7192.
[10] The PyMOL Molecular Graphics System, Version 2.6.0 (open source), Schrödinger, LLC.

Analytical data of products

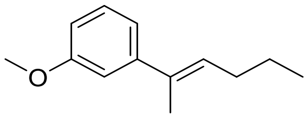

(E)-1-(hex-2-en-2-yl)-3-methoxybenzene ***(2a).*** Following the general procedures A and C sequentially to yield 11.5 mg (30%) of the title compound as a colorless oil. ^{1}H NMR (600 MHz, $CDCl_3$): δ = 7.22 (t, *J* = 8.0 Hz, 1H), 6.98 (ddd, *J* = 7.6, 1.8, 1.0 Hz, 1H), 6.92 (t, *J* = 1.8 Hz, 1H), 6.79-6.78 (m, 1H), 5.79 (td, *J* = 7.2, 1.4 Hz, 1H), 3.82 (s, 3H), 2.14 – 2.19 (m, 2H), 2.02 (d, *J* = 1.3 Hz, 3H), 1.48 (m, 2H), 0.96 (t, *J* = 7.4 Hz, 3H). ^{13}C{^{1}H} NMR (151 MHz, $CDCl_3$): δ = 159.6, 145.8, 134.7, 129.2, 128.9, 118.4, 111.8, 111.8, 55.4, 30.9, 22.9, 21.2, 14.4. HRMS (ESI) m/z: $[M + H]^+$ Calcd for $C_{13}H_{18}O$ 191.1431; Found 191.1427. GC−MS *m*/z: 190 (51), 175 (16), 161 (100), 146 (17), 115 (20), 91 (25), 91 (35), 77 (13).

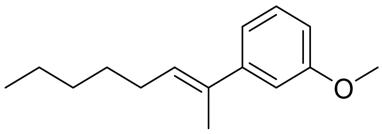

(E)-1-methoxy-3-(oct-2-en-2-yl)benzene ***(2b).*** Following the general procedures A and B sequentially to yield 20 mg (46%) of the title compound as a colorless oil. ^{1}H NMR (600 MHz, $CDCl_3$): δ = 7.22 (t, *J* = 7.9 Hz, 1H), 6.98 (ddd, *J* = 7.8, 1.8, 1.0 Hz, 1H), 6.90 – 6.94 (t, *J* = 2.4 Hz, 1H), 6.77 (ddd, *J* = 8.2, 2.5, 1.0 Hz, 1H), 5.79 (tq, *J* = 7.2, 1.4 Hz, 1H), 3.82 (s, 4H), 2.18 (q, *J* = 8.2, 7.7 Hz, 2H), 2.01 (d, *J* = 1.3 Hz, 3H), 1.41 – 1.50 (m, 2H), 1.26 – 1.39 (m, 4H), 0.88 (t, *J* = 7.2 Hz, 3H). ^{13}C{^{1}H} NMR (151 MHz, $CDCl_3$): δ = 159.8, 146.0, 134.7, 129.4, 129.4, 118.6, 112.0, 55.5, 32.0, 29.6, 29.1, 23.0, 16.2, 14.5. HRMS (ESI) m/z: $[M + H]^+$ Calcd for $C_{15}H_{22}O$ 219.1744; Found 219.1735. GC−MS *m*/z: 219 (7), 218 (43), 203 (4), 162 (15), 161 (100), 148 (47), 136 (47), 135 (19), 117 (14), 91 (35), 77 (13).

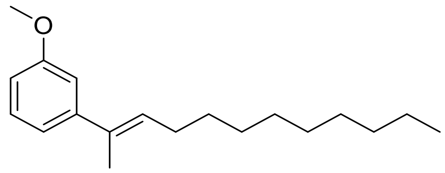

(E)-1-(dodec-2-en-2-yl)-3-methoxybenzene ***(2c).*** Following the general procedures A and B sequentially to yield 20 mg (36%) of the title compound as a colorless oil. ^{1}H NMR (600 MHz, $CDCl_3$): δ = 7.22 (t, *J* = 7.9 Hz, 1H), 6.98 (ddd, *J* = 7.6, 1.7, 1.0 Hz, 1H), 6.93 – 6.90 (m, 1H), 6.77 (ddd, *J* = 8.2, 2.6, 0.9 Hz, 1H), 5.79 (td, *J* = 7.2, 1.4 Hz, 1H), 3.82 (s, 3H), 2.18 (q, *J* = 7.2 Hz, 2H), 2.01 (s, 3H), 1.23 – 1. 30 (m, 14H), 0.87 (t, *J* = 7.2, 3H). ^{13}C{^{1}H} NMR (151 MHz, $CDCl_3$): δ = 159.6, 145.8, 134.5, 129.2, 129.2, 118.4, 111.8, 111.8, 111.1, 53.7, 32.1, 29.8, 29.7, 29.6, 29.5, 28.9, 22.8, 16.0, 14.3. HRMS (ESI) m/z: $[M + H]^+$ Calcd for $C_{19}H_{30}O$ 275.2370; Found 275.2369. GC−MS *m*/z: 274 (31), 161 (100), 148 (73), 136 (56), 135 (31), 115 (12), 91 (25), 77 (6).

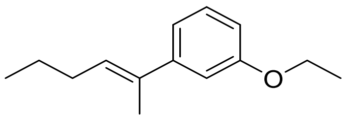

(E)-1-ethoxy-3-(hex-2-en-2-yl)benzene ***(2d).*** Following the general procedures A and B sequentially to yield 22.5 mg (53%) of the title compound as a colorless oil. ^{1}H NMR (600 MHz, $CDCl_3$): δ = 7.20 (t, *J* = 7.9 Hz, 1H), 6.97 (dt, *J* = 7.6, 1.3 Hz, 1H), 6.91 – 6.93 (m, 1H), 6.76 (dd, *J* = 8.2, 2.6 Hz, 1H), 5.79 (tq, *J* = 7.2, 1.4 Hz, 1H), 4.05 (q, *J* = 7.0 Hz, 2H), 2.17 (q, *J* = 7.8 Hz, 2H), 2.01 (d, *J* = 1.3 Hz, 3H), 1.47 (m, 2H), 1.42 (t, *J* = 7.0 Hz, 4H), 0.95 (t, *J* = 7.3 Hz, 3H). ^{13}C{^{1}H} NMR (151 MHz, $CDCl_3$): δ = 157.8, 144.6, 133.5, 127.9, 127.7, 117.1, 111.3, 111.1, 62.3, 29.8, 21.8, 14.8, 13.9, 12.9. GC−MS *m*/z: 204 (55), 176 (10), 175 (65), 147 (100), 119 (49), 91 (59), 77 (25).

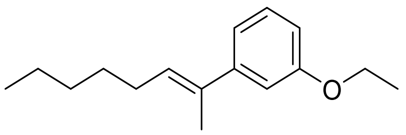

(E)-1-ethoxy-3-(oct-2-en-2-yl)benzene ***(2e).*** Following the general procedures A and B sequentially to yield 32 mg (68%) of the title compound as a colorless oil. ^{1}H NMR (600 MHz, $CDCl_3$): δ = 7.20 (t, *J* = 7.9 Hz, 1H), 6.96 – 6.98 (m, 1H), 6.91 – 6.95 (m, 1H), 6.75 (ddd, *J* = 8.2, 2.6, 1.0 Hz, 1H), 5.79 (tq, *J* = 7.2, 1.5 Hz, 1H), 4.05 (q, *J* = 7.0 Hz, 2H), 2.15 – 2.20 (m, 2H), 2.01 (d, *J* = 1.2 Hz, 3H), 1.42 (m, 9H), 0.87 (t, *J* = 7.0 Hz, 3H). ^{13}C{^{1}H} NMR (151 MHz, $CDCl_3$): δ = 158.8, 148.7, 143.0, 129.0, 118.5, 112.9, 112.7, 112.0, 63.3, 35.4, 31.6, 29.0, 28.2, 22.6, 14.8, 14.0. HRMS (ESI) m/z: [M + H]$^+$ Calcd for $C_{16}H_{24}O$ 233.1900; Found 233.1889. GC−MS *m*/z: 233 (16), 232 (94), 175 (87), 150 (59), 147 (100), 133 (39), 91 (46), 77 (19).

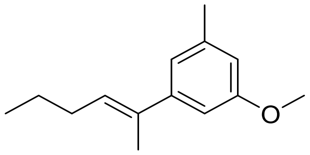

(E)-1-(hex-2-en-2-yl)-3-methoxy-5-methylbenzene ***(2f).*** Following the general procedures A and B sequentially to yield 23 mg (57%) of the title compound as a colorless oil. ^{1}H NMR (400 MHz, $CDCl_3$): δ = 6.80 (t, *J* = 2.0 Hz, 1H), 6.73 (t, *J* = 2.0 Hz, 1H), 6.58 (t, *J* = 2.0 Hz, 1H), 5.77 (ddt, *J* = 7.2, 5.9, 1.4 Hz, 1H), 3.80 (s, 3H), 2.33 (d, *J* = 0.7 Hz, 3H), 2.12 – 2.20 (m, 2H), 2.00 (d, *J* = 1.0 Hz, 3H), 1.46 (m, 2H), 0.95 (t, *J* = 7.3 Hz, 3H). ^{13}C{^{1}H} NMR (151 MHz, $CDCl_3$): δ = 159.8, 145.9, 139.3, 135.0, 129.0, 119.5, 112.9, 109.0, 55.5, 31.2, 23.1, 22.0, 16.3, 14.3. HRMS (ESI) m/z: [M + H]$^+$ Calcd for $C_{14}H_{20}O$ 205.1587; Found 205.1596. GC−MS *m*/z: 204 (69), 189 (29), 175 (100), 160 (24), 128 (14), 105 (20), 91 (19), 77 (10).

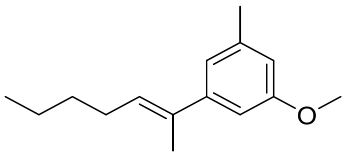

(E)-1-(hept-2-en-2-yl)-3-methoxy-5-methylbenzene ***(2g).*** Following the general procedures A and B sequentially to yield 27 mg (61%) of the title compound as a colorless oil. ^{1}H NMR (400 MHz, $CDCl_3$): δ = 6.80 (s, 1H), 6.72 (s, 1H), 6.59 (s, 1H), 5.77 (td, *J* = 7.2, 1.4 Hz, 1H), 3.80 (s, 3H), 2.33 (d, *J* = 0.7 Hz, 3H), 2.18 (m, 2H), 2.00 (d, *J* = 1.0 Hz, 3H), 1.36 – 1.49 (m, 4H),

0.93 (t, J = 7.1 Hz, 3H). $^{13}C\{^1H\}$ NMR (101 MHz, $CDCl_3$): δ = 159.5, 148.9, 142.9, 139.1, 119.6, 113.3, 111.9, 109.2, 55.2, 35.4, 31.6, 27.9, 22.5, 21.7, 14.1. HRMS (ESI) m/z: $[M + H]^+$ Calcd for $C_{15}H_{22}O$ 219.1744; Found 219.1752. GC−MS *m*/z: 218 (59), 176 (15), 175 (100), 145 (22), 105 (17), 91 (15), 77 (7).

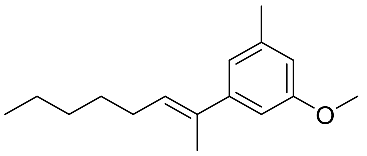

(E)-1-methoxy-3-methyl-5-(oct-2-en-2-yl)benzene ***(2h).*** Following the general procedures A and B sequentially to yield 32.5 mg (70%) of the title compound as a colorless oil. ^1H NMR (400 MHz, $CDCl_3$): δ = 6.70 (t, J = 2.0 Hz, 1H), 6.66 (t, J = 2.3 Hz, 1H), 6.53 (t, J = 2.0 Hz, 1H), 5.66 – 5.74 (m, 1H), 3.73 (s, 3H), 2.26 (d, J = 0.6 Hz, 3H), 2.10 (s, 2H), 1.93 (d, J = 1.4 Hz, 3H), 1.21 – 1.30 (m, 6H), 0.81 (t, J = 7.2 Hz, 3H). $^{13}C\{^1H\}$ NMR (101 MHz, $CDCl_3$): δ = 159.7, 143.1, 139.2, 129.0, 119.7, 113.5, 112.1, 109.3, 55.3, 31.8, 29.4, 28.9, 22.8, 21.8, 16.0, 14.2. HRMS (ESI) m/z: $[M + H]^+$ Calcd for $C_{16}H_{24}O$ 233.1900; Found 233.1907. GC−MS *m*/z: 232 (32), 217 (6), 176 (11), 175 (100), 150 (34), 149 (23), 115 (24), 91 (24), 77 (11).

Compound 2h was also prepared on a 1 mmol scale as follows: A 50 mL pressure tube was charged with of 2-methoxy-4-methylbenzoic acid (166 mg, 1 mmol, 1 equiv), 1-octene (0.47 mL, 3 mmol, 3 equiv), Ag_2CO_3 (55 mg, 0.2 mmol, 0. 2 equiv), DABCO (224 mg, 2 mmol, 2 equiv), $[Cp*RhCl_2]_2$ (30 mg, 0.05 mmol, 0.05 equiv), and toluene (9 mL). The resulting mixture was heated to 70°C in an oil bath. After 48 h, the reaction was cooled to room temperature. The obtained crude product was purified via flash column chromatography (90 g silica, LP, and EtOAc 0−10%). The obtained mixture of isomers was dissolved in $CHCl_3$ (0.1 M) and trifluoro acetic acid (58 μL) was added. Subsequently, the reaction mixture was refluxed for 5h. After cooling to room temperature, the solvent was evaporated. The residue was dissolved in dichloromethane and filtered off on a short celite pad. Finally, the solvent was evaporated to

yield 139 mg (60%) of the title compound as a colorless oil. Analytical data were in accordance with the previous finding.

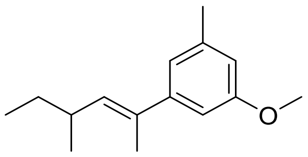

(E)-1-methoxy-3-methyl-5-(4-methylhex-2-en-2-yl)benzene ***(2i).*** Following the general procedures A and B sequentially to yield 24 mg (55%) of the title compound as a colorless oil. ^{1}H NMR (600 MHz, $CDCl_3$): δ = 6.81 (s, 1H), 6.73 (s, 1H), 6.60 (s, 1H), 5.53 (dq, *J* = 9.5, 1.3 Hz, 1H), 3.80 (s, 3H), 2.39 – 2.46 (m, 1H), 2.33 (s, 3H), 2.01 (d, *J* = 1.3 Hz, 3H), 1.37 – 1.47 (m, 2H), 1.01 (d, *J* = 6.7 Hz, 3H), 0.89 (t, *J* = 7.4 Hz, 3H). ^{13}C{^{1}H} NMR (151 MHz, $CDCl_3$): δ = 139.1, 135.2, 119.3, 112.7, 108.9, 55.3, 34.9, 30.6, 21.8, 20.8, 16.3, 12.2. HRMS (ESI) m/z: $[M + H]^+$ Calcd for $C_{15}H_{22}O$ 219.1744; found 219.1720. GC−MS *m*/z: 218 (45), 190 (15), 189 (100), 174 (23), 129 (10), 115 (14), 91 (14).

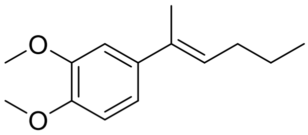

(E)-4-(hex-2-en-2-yl)-1,2-dimethoxybenzene ***(2j).*** Following the general procedures A and B sequentially to yield 20 mg (45%) of the title compound as a colorless oil. ^{1}H NMR (600 MHz, $CDCl_3$): δ = 6.94 (d, *J* = 2.4 Hz, 1H), 6.96 – 6.91 (m, 1H), 6.82 (d, *J* = 8.9 Hz, 1H), 5.72 (tq, *J* = 7.3, 1.4 Hz, 1H), 3.90 (s, 3H), 3.88 (s, 3H), 2.17 (m, 1H), 2.01 (d, *J* = 1.2 Hz, 3H), 1.48 (m, 2H), 0.96 (t, *J* = 7.3 Hz, 3H). ^{13}C{^{1}H} NMR (151 MHz, $CDCl_3$): δ = 147.5, 146.9, 136.1, 133.2, 126.3, 116.8, 109.9, 108.0, 54.9, 54.8, 28.7, 21.9, 14.9, 12.9. HRMS (ESI) m/z: $[M + H]^+$ Calcd for $C_{14}H_{20}O_2$ 221.1536; Found 221.1527. GC−MS *m*/z: 220 (62), 191 (100), 189 (24), 176 (8), 160 (49), 145 (14), 115 (16), 91 (17).

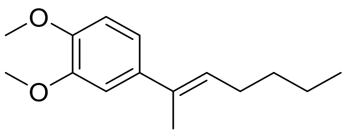

(E)-4-(hept-2-en-2-yl)-1,2-dimethoxybenzene ***(2k).*** Following the general procedures A and B sequentially to yield 23 mg (49%) of the title compound as a colorless oil. ^{1}H NMR (400 MHz, $CDCl_3$): δ = 6.95 (d, *J* = 2.4 Hz, 1H), 6.83 (dd, *J* = 8.5, 2.4 Hz, 1H), 6.82 (d, *J* = 8.5 Hz, 1H), 5.72 (td, *J* = 7.2, 1.2 Hz, 1H), 3.89 (s, 3H), 3.88 (s, 3H), 2.15 – 2.22 (m, 2H), 2.01 (d, *J* = 0.9 Hz, 3H), 1.31 (t, *J* = 3.7 Hz, 4H), 0.93 (t, *J* = 7.1 Hz, 3H). ^{13}C{^{1}H} NMR (101 MHz, $CDCl_3$): δ = 149.0, 148.3, 137.6, 134.5, 128.0, 118.3, 111.3, 109.5, 56.4, 56.3, 32.4, 28.9, 22.9, 16.4, 14.5. HRMS (ESI) m/z: $[M-H]^-$ Calcd for $C_{15}H_{22}O_2$ 233.1547; Found 233.1537. GC−MS *m*/z: ; 234 (53), 191 (100), 160 (69), 115 (32), 91 (41), 77 (27), 55 (24).

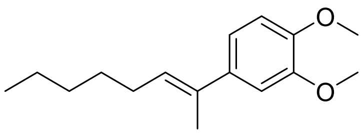

(E)-1,2-dimethoxy-4-(oct-2-en-2-yl)benzene ***(2l).*** Following the general procedures A and B sequentially to yield 25 mg (51%) of the title compound as a colorless oil. ^{1}H NMR (600 MHz, $CDCl_3$): δ = 6.93 (d, *J* = 2.4 Hz, 1H), 6.92-6.93 (m, 1H), 6.82 (d, *J* = 8.9 Hz, 1H), 5.72 (tq, *J* = 7.2, 1.5 Hz, 1H), 3.90 (s, 3H), 3.88 (s, 3H), 2.15 – 2.20 (m, 2H), 2.01 (d, *J* = 1.2 Hz, 3H), 1.45 (m, 2H), 1.29 – 1.39 (m, 6H), 0.89 (t, *J* = 7.2 Hz, 3H). ^{13}C{^{1}H} NMR (151 MHz, $CDCl_3$): δ = 148.7, 148.0, 137.3, 134.2, 127.7, 117.9, 111.0, 109.2, 56.1, 56.0, 31.8, 29.5, 28.9, 22.8, 16.1, 14.2. HRMS (ESI) m/z: $[M + H]^+$ Calcd for $C_{16}H_{24}O_2$ 249.1849; Found 249.1846. GC−MS *m*/z: 248 (55), 217 (15), 191 (100), 178 (26), 161 (22), 160 (44), 151 (7), 115 (14), 91 (15).

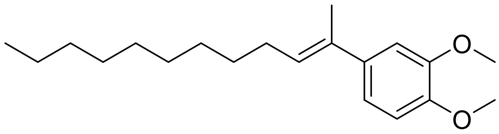

(E)-4-(dodec-2-en-2-yl)-1,2-dimethoxybenzene ***(2m).*** Following the general procedures A and B sequentially to yield 29 mg (47%) of the title compound as a colorless oil. ^{1}H NMR (600

MHz, $CDCl_3$): δ = 6.92 (d, *J* = 2.5 Hz, 1H), 6.82 (d, *J* = 8.9 Hz, 1H), 6.76 – 6.70 (m, 1H), 5.72 (ddt, *J* = 7.3, 6.0, 1.4 Hz, 1H), 3.90 (s, 3H), 3.88 (s, 3H), 2.17 (q, *J* = 7.7 Hz, 2H), 2.01 (d, *J* = 1.2 Hz, 3H), 1.26 – 1.34 (m, 14H), 0.88 (t, *J* = 7.2 Hz, 3H). $^{13}C\{^1H\}$ NMR (151 MHz, $CDCl_3$): δ = 148.5, 147.9, 137.2, 134.0, 127.6, 117.8, 110.9, 109.0, 55.9, 55.8, 31.9, 29.7, 29.6, 29.6, 29.5, 29.4, 28.8, 22.7, 15.9, 14.1. HRMS (ESI) m/z: $[M + H]^+$ Calcd for $C_{20}H_{32}O_2$ 305.2475; Found 305.2468. GC−MS *m*/z: 304 (40), 237 (10), 191 (100), 178 (43), 165 (44), 161 (20), 147 (5), 115 (9), 91 (10).

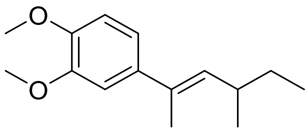

(E)-1,2-dimethoxy-4-(4-methylhex-2-en-2-yl)benzene ***(2n).*** Following the general procedures A and B sequentially to yield 20 mg (43%) of the title compound as a colorless oil. ^1H NMR (400 MHz, $CDCl_3$): δ = 6.95 (d, *J* = 2.0 Hz, 1H), 6.93 (d, *J* = 8.6 Hz, 1H), 6.82 (dd, *J* = 8.6, 2.0 Hz, 1H), 5.48 (dq, *J* = 9.5, 1H), 3.91 (s, 3H), 3.88 (s, 3H), 2.34 – 2.50 (m, 1H), 2.02 (d, *J* = 1.3 Hz, 3H), 1.02 (d, *J* = 6.7 Hz, 3H), 0.90 (t, *J* = 7.4 Hz, 9H). $^{13}C\{^1H\}$ NMR (101 MHz, $CDCl_3$): δ = 148.9, 148.4, 133.6, 132.5, 123.1, 110.7, 109.0, 56.3, 55.7, 34.7, 30.4, 20.5, 15.6, 11.9. HRMS (ESI) m/z: $[M-H]^-$ Calcd for $C_{15}H_{22}O_2$ 233.1547; Found 233.1550. GC−MS *m*/z: 234 (38), 205 (100), 203 (24), 174 (54), 159 (26), 115 (19), 91 (30), 77 (18), 55 (10).

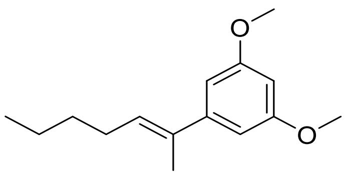

(E)-1-(hept-2-en-2-yl)-3,5-dimethoxybenzene ***(2o).*** Following the general procedures A and B sequentially to yield 29 mg (62%) of the title compound as a colorless oil. ^1H NMR (600 MHz, $CDCl_3$): δ = 6.53 (d, *J* = 2.3 Hz, 2H), 6.35 (t, *J* = 2.3 Hz, 1H), 5.79 (td, *J* = 7.2, 1.3 Hz, 1H), 3.80 (s, 6H), 2.18 (q, *J* = 7.6 Hz, 2H), 2.00 (s, 3H), 1.40 – 1.45 (m, 2H), 1.34 – 1.39 (m, 2H), 0.92 (d, *J* = 7.2 Hz, 3H). $^{13}C\{^1H\}$ NMR (151 MHz, $CDCl_3$): δ = 149.0, 148.3, 137.6, 134.5,

128.0, 118.3, 111.3, 109.5, 56.4, 56.3, 32.4, 29.0, 22.9, 16.4, 14.5. HRMS (ESI) m/z: [M-H]$^-$ Calcd for $C_{15}H_{22}O_2$ 233.1547; Found 233.1546. GC−MS *m*/z: 235 (11), 234 (75), 205 (40), 191 (100), 166 (15), 161 (25), 121 (26), 91 (28), 77 (18).

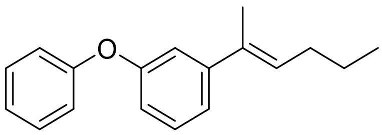

(E)-1-(hex-2-en-2-yl)-3-phenoxybenzene ***(2p).*** Following the general procedures A and B sequentially to yield 18 mg (36%) of the title compound as a colorless oil. ^{1}H NMR(400 MHz, $CDCl_3$): δ = 7.36 – 7.31 (m, 2H), 7.18 – 7.14 (m, 1H), 7.12 – 7.08 (m, 2H), 7.05 – 6.99 (m, 3H), 6.89 (ddd, *J* = 8.0, 2.4, 1.0 Hz, 1H), 5.80 (tq, *J* = 7.3, 1.5 Hz, 1H), 2.19 – 2.13 (m, 3H), 2.00 (s, 2H), 1.48 – 1.40 (m, 2H), 0.95 (t, *J* = 7.4 Hz, 3H). ^{13}C{^{1}H} NMR (101 MHz, $CDCl_3$): δ = 157.5, 157.0, 143.6, 135.7, 129.7, 129.7, 121.2, 118.7, 117.7, 117.6, 114.5, 112.5, 30.4, 22.4, 15.7, 13.9. HRMS (ESI) m/z: [M + H]$^+$ Calcd for $C_{18}H_{30}O$ 252.1464; Found 252.1457. GC−MS *m*/z: 252 (80), 223 (43), 181 (34), 159 (47), 129 (100), 115 (75), 91 (29), 77 (40).

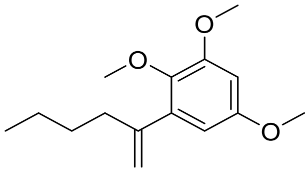 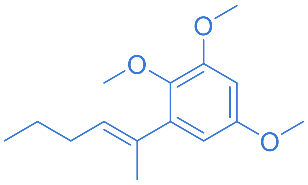

1-(hex-1-en-2-yl)-2,3,5-trimethoxybenzene (exo isomer) ***(3q)*** and *(E)-1-(hex-2-en-2-yl)-2,3,5-trimethoxybenzene (endo isomer)* ***(2q).*** Following the general procedure A to yield 14 mg (28%) of the title compounds in favor of exo isomer (ratio = 1:2.3) as a colorless oil (The isomerization reaction for this series of compound was not successful). ^{1}H NMR (600 MHz, $CDCl_3$): δ = 6.43 (d, *J* = 2.9 Hz, 1H), 6.40 (d, *J* = 3.0 Hz, $1H_{endo}$), 6.30 (d, *J* = 3.0 Hz, $1H_{endo}$), 6.25 (d, *J* = 2.9 Hz, 1H), 5.50 (td, *J* = 7.2, 1.3 Hz, 1H), 5.12 (q, *J* = 1.6 Hz, 1H), 5.03 (d, *J* = 2.0 Hz, 1H), 3.84 (s, 3H), 3.83 (s, $3H_{endo}$), 3.78 (s, 3H), 3.75 (s, $3H_{endo}$), 3.70 (s, 3H), 3.68 (s, $3H_{endo}$), 2.47 (t, *J* = 7.0 Hz, 2H), 2.01 – 2.03 (m, $2H_{endo}$), 1.99 (d, *J* = 1.3 Hz, $3H_{endo}$), 1.47 (q, *J* = 7.3 Hz, $2H_{endo}$),

1.31 – 1.38 (m, 4H), 0.96 (t, *J* = 7.4 Hz, $3H_{endo}$), 0.87 (t, *J* = 7.2 Hz, 3H). $^{13}C\{^{1}H\}$ NMR (151 MHz, $CDCl_3$): δ = 155.7, 153.2, 148.8, 140.2, 137.7, 130.2, 120.2, 114.2, 113.9, 105.2, 104.8, 98.9, 98.3, 78.5, 60.9, 60.7, 55.9, 55.8, 55.6, 55.5, 36.4, 31.3, 30.3, 29.7, 23.5, 22.4, 17.3, 14.0, 13.9. HRMS (ESI) m/z: $[M + H]^+$ Calcd for $C_{15}H_{22}O_3$ 251.1642; Found 251.1632. $GC{-}MS_{exo}$ *m/z*: 251 (15), 250 (100), 235 (40), 219 (12), 208 (45), 165 (17), 151 (73), 121 (14), 91 (17). $GC{-}MS_{endo}$ *m/z*: 250 (100), 235 (42), 219 (41), 193 (31), 191 (14), 151 (35), 91 (23), 69 (19).

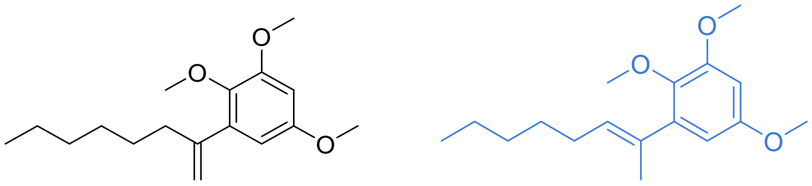

1,2,5-trimethoxy-3-(oct-1-en-2-yl)benzene (exo isomer) ***(3r)*** and *(E)-1,2,5-trimethoxy-3-(oct-2-en-2-yl)benzene (endo isomer)* ***(2r)*****.** Following the general procedure A to yield 23 mg (42%) of the title compounds in favor of exo isomer (ratio = 1:2.3) as a colorless oil (The isomerization reaction for this series of compound was not successful). ^1H NMR (600 MHz, $CDCl_3$): δ = 6.51 (d, *J* = 3.0 Hz, $1H_{endo}$), 6.43 (d, *J* = 2.9 Hz, 1H), 6.34 (d, *J* = 3.0 Hz, $1H_{endo}$), 6.25 (d, *J* = 2.9 Hz, 1H), 5.31 (tq, *J* = 7.8, 1.5 Hz, $1H_{endo}$), 5.13 – 5.11 (m, 1H), 5.02 (d, *J* = 2.2 Hz, 1H), 3.84 (s, 3H), 3.81 (s, $3H_{endo}$), 3.78 (s, 3H), 3.75 (s, $3H_{endo}$), 3.72 (s, $3H_{endo}$), 3.70 (s, 3H), 2.46 (t, *J* = 7.7 Hz, 2H), 2.23 (t, *J* = 7.8 Hz, $2H_{endo}$), 2.17 (s, $3H_{endo}$), 1.32 – 1.39 (m, $6H_{endo}$), 1.24 – 1.26 (m, 8H), 0.89 (t, *J* = 7.2 Hz, $3H_{endo}$), 0.86 (t, *J* = 7.2 Hz, 3H). $^{13}C\{^{1}H\}$ NMR (151 MHz, $CDCl_3$): δ = 157.9, 155.6, 153.2, 148.8, 140.1, 137.6, 135.4, 113.9, 113.8, 105.1, 98.8, 60.8, 60.3, 55.7, 55.5, 55.0, 50.9, 36.6, 31.6, 29.6, 28.9, 28.0, 27.7, 22.6, 21.0, 15.7, 14.1, 14.0. HRMS (ESI) m/z: $[M-H]^-$ Calcd for $C_{17}H_{26}O_3$ 277.1809; Found 277.1812. $GC{-}MS_{exo}$ *m/z*: 279 (18), 278 (100), 263 (29), 247 (9), 208 (73), 193 (58), 165 (20), 151 (64), 121 (11), 91 (13). $GC{-}MS_{endo}$ *m/z*: 278 (100), 263 (38), 247 (36), 221 (21), 191 (27), 165 (34), 150 (33), 115 (13), 91 (20).

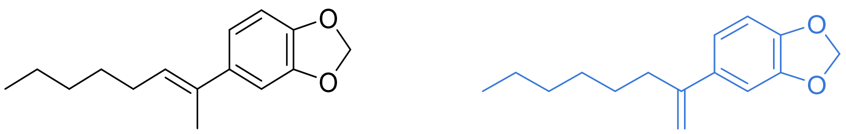

(E)-5-(oct-2-en-2-yl)benzo[d][1,3]dioxole (endo isomer) ***(2s)*** and *4-(oct-1-en-2-yl)benzo[d][1,3]dioxole (exo isomer)* ***(3s)*****.** Following the general procedure A to yield 30.5 mg (66%) of the title compounds in favor of endo isomer (ratio = 1:0.6) as a bright yellow oil (The isomerization reaction for this series of compound was not successful). ^{1}H NMR (600 MHz, $CDCl_3$): δ = 6.91 (d, *J* = 1.8 Hz, $1H_{exo}$), 6.88 (dd, *J* = 7.6, 1.9 Hz, 2H), 6.84 (dd, *J* = 8.1, 1.8 Hz, $1H_{endo}$), 6.76 (d, *J* = 8.1 Hz, $1H_{exo}$), 6.75 (d, *J* = 8.1 Hz, $1H_{endo}$), 5.95 (s, $1H_{exo}$), 5.93 (s, $2H_{endo}$), 5.68 (ddt, *J* = 7.2, 5.7, 1.3 Hz, $1H_{endo}$), 5.16 (d, *J* = 1.6 Hz, $1H_{exo}$), 4.96 (q, *J* = 1.3 Hz, $1H_{exo}$), 2.43 (td, *J* = 7.6, 1.3 Hz, $2H_{exo}$), 2.13 – 2.18 (m, $2H_{endo}$), 1.98 (d, *J* = 1.2 Hz, $3H_{endo}$), 1.38 – 1.46 (m, $8H_{exo}$), 1.30 – 1.35 (m, $6H_{endo}$), 0.89 (t, *J* = 7.2 Hz, $3H_{endo}$), 0.87 (t, *J* = 7.0 Hz, $3H_{exo}$). ^{13}C{^{1}H} NMR (151 MHz, $CDCl_3$): δ = 147.2, 146.6, 146.5, 145.8, 145.1, 137.6, 134.7, 132.9, 126.9, 118.5, 117.8, 110.1, 106.9, 106.8, 105.7, 105.3, 99.9, 99.8, 34.6, 30.6, 30.6, 28.7, 28.3, 28.0, 27.7, 27.2, 21.6, 15.0, 13.2, 13.1. HRMS (ESI) m/z: $[M + H]^+$ Calcd for $C_{15}H_{20}O_2$ 233.1536; Found 233.1540. GC−MS_{endo} *m/z*: 232 (29), 175 (32), 162 (31), 145 (100), 117 (51), 115 (36), 91 (21), 77 (9). GC−MS_{exo} *m/z*: 232 (8), 162 (100), 145 (10), 103 (10), 77 (7).

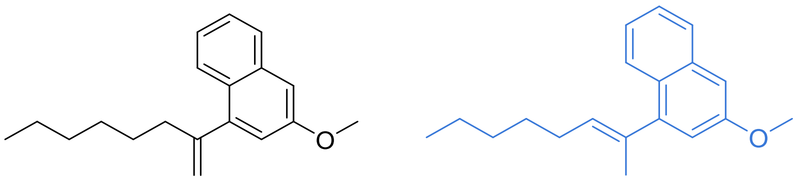

3-methoxy-1-(oct-1-en-2-yl)naphthalene (exo isomer) ***(3t)*** and *(E)-3-methoxy-1-(oct-2-en-2-yl)naphthalene (endo isomer)* ***(2t)*****.** Following the general procedure A to yield 14.5 mg (29%) of the title compounds in favor of exo isomer (ratio = 1:2.9) as a colorless oil (The isomerization reaction for this series of compound was not successful). ^{1}H NMR (400 MHz, $CDCl_3$): δ = 7.94 (d, *J* = 8.4 Hz, 1H), 7.88 (d, *J* = 8 Hz, $1H_{endo}$), 7.75 (d, *J* = 9.6 Hz, 1H), 7.40-7.44 (m, 1H), 7.29-7.33 (m, 1H), 7.06 (d, *J* = 2.4 Hz, 1H), 7.04 (d, *J* = 2.8 Hz, $1H_{endo}$), 6.95 (d, *J* = 2.4 Hz, 1H),

6.94 (d, J = 2.8 Hz, $1H_{endo}$), 5.49 –5.53 (td, J = 7.3, 1.4 Hz, $1H_{endo}$), 5.37 – 5.35 (m, 1H), 5.05 (d, J = 2.1 Hz, 1H), 3.93 (s, 3H), 3.92 (s, $3H_{endo}$), 2.48 (t, J = 8.1 Hz, 2H), 2.26 (q, J = 7.2 Hz, $2H_{endo}$), 2.06 (d, J = 1.5 Hz, $3H_{endo}$), 1.45 – 1.38 (m, $6H_{endo}$), 1.30 – 1.21 (m, 8H), 0.91 (t, J = 7.2 Hz, $3H_{endo}$), 0.83 (t, J = 6.8 Hz, 3H). $^{13}C\{^{1}H\}$ NMR (101 MHz, $CDCl_3$): δ = 157.5, 148.8, 148.1, 143.6, 141.6, 135.3, 135.0, 127.3, 126.3, 125.9, 123.5, 117.9, 117.8, 115.3, 115.2, 105.0, 104.9, 58.1, 55.4, 38.6, 31.8, 31.8, 29.4, 29.2, 28.2, 28.0, 23.0, 22.8, 17.0, 14.8, 14.2. HRMS (ESI) m/z: $[M + H]^+$ Calcd for $C_{19}H_{24}O$ 269.1900; Found 269.1895. GC−MS_{exo} *m/z*: 268 (37), 198 ,(25), 197 (100), 184 (61), 183 (36), 153 (20), 115 (10). GC−MS_{endo} *m/z*: 268 (45), 211 (100), 197 (41), 165 (38), 152 (21), 115 (10).

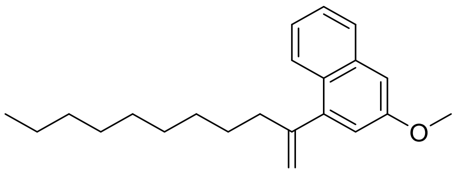

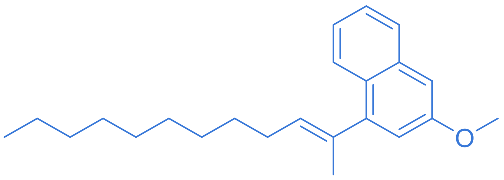

3-methoxy-1-(undec-1-en-2-yl)naphthalene (exo isomer) **(3u)** and *(E)-1-(dodec-2-en-2-yl)-3-methoxynaphthalene (endo isomer)* ***(2u)*****.** Following the general procedure A to yield 16 mg (26%) of the title compounds in favor of exo isomer (ratio = 1:3) as a colorless oil (The isomerization reaction for this series of compound was not successful). ^{1}H NMR (400 MHz, $CDCl_3$): δ = 7.86 (d, J = 8.5 Hz, $1H_{endo}$), 8.17 (d, J = 8.5 Hz, 1H), 7.80 (d, J = 8.5 Hz, $1H_{endo}$), 7.66 (d, J = 8.1 Hz, 1H), 7.35 (ddd, J = 8.1, 6.8, 1.3 Hz, 1H), 7.24 (ddd, J = 8.2, 6.7, 1.3 Hz, 1H), 6.99 (d, J = 2.5 Hz, 1H), 6.96 (d, J = 2.6 Hz, $1H_{endo}$), 6.88 (d, J = 2.6 Hz, $1H_{endo}$), 6.87 (td, J = 7.3, 1.5 Hz, $1H_{endo}$), 5.29 (q, J = 1.4 Hz, 1H), 4.98 (d, J = 2.0 Hz, 1H), 3.86 (s, 3H), 3.85 (s, $3H_{endo}$), 2.41 (t, J = 8.0 Hz, 2H), 2.19 (q, J = 7.2 Hz, $2H_{endo}$), 1.99 (d, J = 1.5 Hz, $3H_{endo}$), 1.30-1.38 (m, $12H_{endo}$), 1.16-1.18 (m, 14H), 0.79 (t, J = 7.1 Hz, 3H). $^{13}C\{^{1}H\}$ NMR (101 MHz, $CDCl_3$): δ = 156.7, 153.2, 136.1, 135.0, 133.8, 127.1, 126.6, 126.2, 125.8, 123.5, 117.8, 115.1, 104.8, 55.3, 38.4, 31.9, 29.7, 29.6, 29.6, 29.4, 29.3, 29.3, 28.1, 22.7, 20.1, 14.1. HRMS (ESI) m/z: $[M + H]^+$ Calcd for $C_{22}H_{30}O$ 311.2370; found 311.2365. GC−MS_{exo} *m/z*: 325 ,(8), 324

(32), 197 (100), 184 (65), 183 (34), 165 (20), 152 (9), 115 (5). GC−MS_{endo} *m/z*: 324 (35), 211 (100), 197 (37), 165 (23), 153 (11).

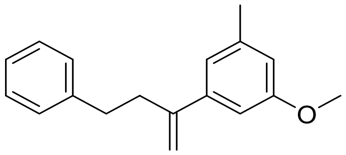

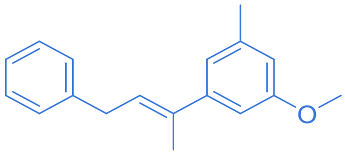

1-methoxy-3-methyl-5-(4-phenylbut-1-en-2-yl)benzene (exo isomer) ***(3v)*** and *(E)-1-methoxy-3-methyl-5-(4-phenylbut-2-en-2-yl)benzene (endo isomer)* ***(2v).*** Following the general procedure A to yield 25 mg (49%) of the title compounds in favor of exo isomer (ratio = 1:2.5) as a light yellow oil (The isomerization reaction for this series of compound was not successful). ^{1}H NMR (400 MHz, $CDCl_3$): δ = 7.34 – 7.37 (m, 1H), 7.29 (dd, *J* = 7.3, 1.3 Hz, 2H), 7.16 – 7.21 (m, 2H), 6.84 – 6.86 (dd, *J* = 1.6, 0.8 Hz, 1H), 6.82-6.84 (dd, *J* = 2.4, 1.6 Hz, $1H_{endo}$), 6.77 (dd, *J* = 1.6, 0.8 Hz, 1H), 6.75 (dd, *J* = 2.4, 1.6 Hz, $1H_{endo}$), 6.66 (dd, *J* = 1.6, 0.8 Hz, 1H), 6.63 (dd, *J* = 2.4, 1.6 Hz, $1H_{endo}$), 5.96 (td, *J* = 7.4, 1.4 Hz, $1H_{endo}$), 5.28 (d, *J* = 1.5 Hz, 1H), 5.04 (d, *J* = 1.5 Hz, 1H), 3.81 (s, 3H), 3.79 (s, $3H_{endo}$), 3.56 (d, *J* = 7.0 Hz, $2H_{endo}$), 2.35 (d, *J* = 0.7 Hz, 3H), 2.31 – 2.33 (m, 2H), 2.12 (d, *J* = 1.2 Hz, $3H_{endo}$). ^{13}C{^{1}H} NMR (101 MHz, $CDCl_3$): δ = 159.5, 147.8, 142.4, 141.9, 139.2, 135.1, 128.3, 128.2, 126.1, 125.8, 120.6, 119.5, 113.5, 112.6, 112.0, 110.2, 109.1, 55.1, 55.1, 42.6, 37.3, 34.7, 29.6, 21.6, 21.6. HRMS (ESI) m/z: $[M + H]^+$ Calcd for $C_{18}H_{20}O$ 253.1587; Found 253.1571. GC−MS_{exo} *m/z*: 252 (35), 237 (14), 148 (12), 130 (31), 115 (17), 91 (100). GC−MS_{endo} *m/z*:252 (81), 237 (78), 221 (25), 178 (17), 159 (33), 129 (54), 115 (100), 105 (20), 91 (66), 77 (19).

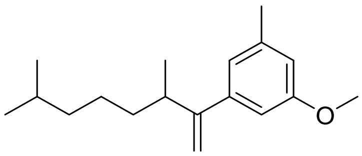

1-(3,7-dimethyloct-1-en-2-yl)-3-methoxy-5-methylbenzene ***(3w).*** Following the general procedure A to yield 18 mg (33%) of the title compounds as a colorless oil.^{1}H NMR (600 MHz,

$CDCl_3$): δ = 6.74 (s, 1H), 6.67 (s, 1H), 6.63 (s, 1H), 5.14 (d, *J* = 1.3 Hz, 1H), 4.98 (t, *J* = 1.2 Hz, 1H), 3.79 (s, 3H), 2.61 (m, 1H), 2.32 (s, 1H), 1.54 (s, 6H), 1.09 (d, *J* = 6.9 Hz, 4H), 0.83 (d, *J* = 6.6 Hz, 9H). $^{13}C\{^1H\}$ NMR (151 MHz, $CDCl_3$): δ = 159.5, 158.4, 154.1, 143.6, 137.9, 119.1, 112.1, 109.7, 108.7, 54.2, 37.0, 35.3, 28.7, 26.9, 24.0, 21.7, 20.6, 19.1. HRMS (ESI) m/z: $[M + H]^+$ Calcd for $C_{17}H_{26}O$ 247.2057; Found 247.2048. GC−MS *m*/*z*: 260 (11), 203 (14), 176 (100), 161 (76), 150 (63), 145 (49), 135 (30), 115 (22), 91 (22), 77 (10).

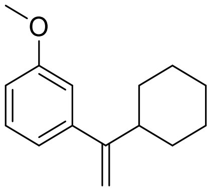

1-(1-cyclohexylvinyl)-3-methoxybenzene ***(3x).*** Following the general procedure A to yield 12 mg (27%) of the title compounds as a colorless oil. ^1H NMR (400 MHz, $CDCl_3$): δ = 7.23 (t, *J* = 7.9 Hz, 1H), 6.92 (ddd, *J* = 7.7, 1.7, 1.0 Hz, 1H), 6.87 (dd, *J* = 2.7, 1.5 Hz, 1H), 6.81 (ddd, *J* = 8.2, 2.6, 1.0 Hz, 1H), 5.13 (t, *J* = 1.4 Hz, 1H), 4.99 (t, *J* = 1.4 Hz, 1H), 3.82 (s, 3H), 2.39 (t, *J* = 11.7 Hz, 1H), 1.68 – 1.85 (m, 5H), 1.11 – 1.22 (m, 5H). $^{13}C\{^1H\}$ NMR (101 MHz, $CDCl_3$): δ = 159.2, 155.0, 142.8, 129.0, 119.2, 112.7, 112.1, 110.4, 55.2, 42.6, 32.7, 26.8, 26.4. HRMS (ESI) m/z: $[M + H]^+$ Calcd for $C_{15}H_{20}O$ 217.1587; Found 217.1604. GC−MS *m*/*z*: 217 (14), 216 (100), 201 (33), 173 (53), 159 (42), 148 (73), 134 (51), 121 (39), 108 (18), 91 (44), 77 (28), 55 (17).

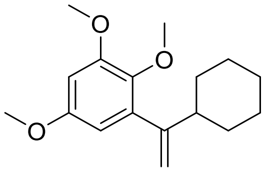

1-(1-cyclohexylvinyl)-2,3,5-trimethoxybenzene ***(3y).*** Following the general procedure A to yield 14 mg (25%) of the title compounds as a colorless oil. ^1H NMR (400 MHz, $CDCl_3$): δ = 6.35 (d, *J* = 2.9 Hz, 1H), 6.13 (d, *J* = 2.9 Hz, 1H), 5.03 (t, *J* = 1.5 Hz, 1H), 4.90 (d, *J* = 1.2 Hz, 1H), 3.77 (s, 3H), 3.71 (s, 3H), 3.62 (s, 3H), 1.88 –2.01 (m, 1H), 1.69 – 1.78 (m, 4H), 1.09 – 1.31

(m, 4H). $^{13}C\{^1H\}$ NMR (101 MHz, $CDCl_3$): δ = 153.7, 153.0, 147.2, 140.3, 115.9, 111.5, 103.8, 100.6, 60.1, 65.5, 55.9, 41.8, 32.6, 26.2, 26.0. GC−MS *m*/*z*: 276 (100), 245 (85), 179 (63), 164 (30), 91 (24), 55 (49).

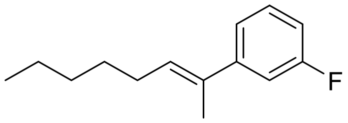

(E)-1-fluoro-3-(oct-2-en-2-yl)benzene ***(2z)***. Following the general procedures A and B sequentially to yield 41 mg (21%) of the title compound as a colorless oil. ^1H NMR (400 MHz, $CDCl_3$): δ = 7.25 – 7.22 (m, 1H), 7.16 – 7.13 (m, 1H), 7.09 – 7.05 (m, 1H), 6.90 (tdd, *J* = 8.3, 2.6, 1.1 Hz, 1H), 5.82 (tq, *J* = 7.3, 1.4 Hz, 1H), 2.19 (q, *J* = 7.3 Hz, 1H), 2.01 (d, *J* = 1.3 Hz, 3H), 1.49 – 1.41 (m, 4H), 1.38 – 1.31 (m, 2H), 0.93 – 0.89 (t, *J* = 7.2 Hz, 3H). $^{13}C\{^1H\}$ NMR (101 MHz, $CDCl_3$): δ = 164.3 (d, *J* = 246.4 Hz), 146.6 (d, *J* = 7.1 Hz), 133.6 (d, *J* = 2.0 Hz), 130.0, 129.5 (d, *J* = 8.1 Hz), 121.3 (d, *J* = 3.0 Hz), 113.1 (d, *J* = 21.2 Hz),112.5 (d, *J* = 22.2 Hz), 31.8, 29.3, 28.9, 22.7, 15.8, 14.2. ^{19}F NMR (376 MHz, $CDCl_3$): δ = -114.07. GC−MS *m*/*z*: 206 (24), 149 (87), 136 (100), 135 (25), 133 (12), 109 (46), 90 (7).

NMR Spectra

^{1}H-NMR (E)-1-(hex-2-en-2-yl)-3-methoxybenzene (2a)

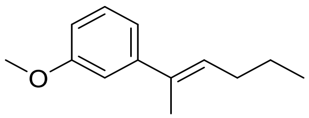

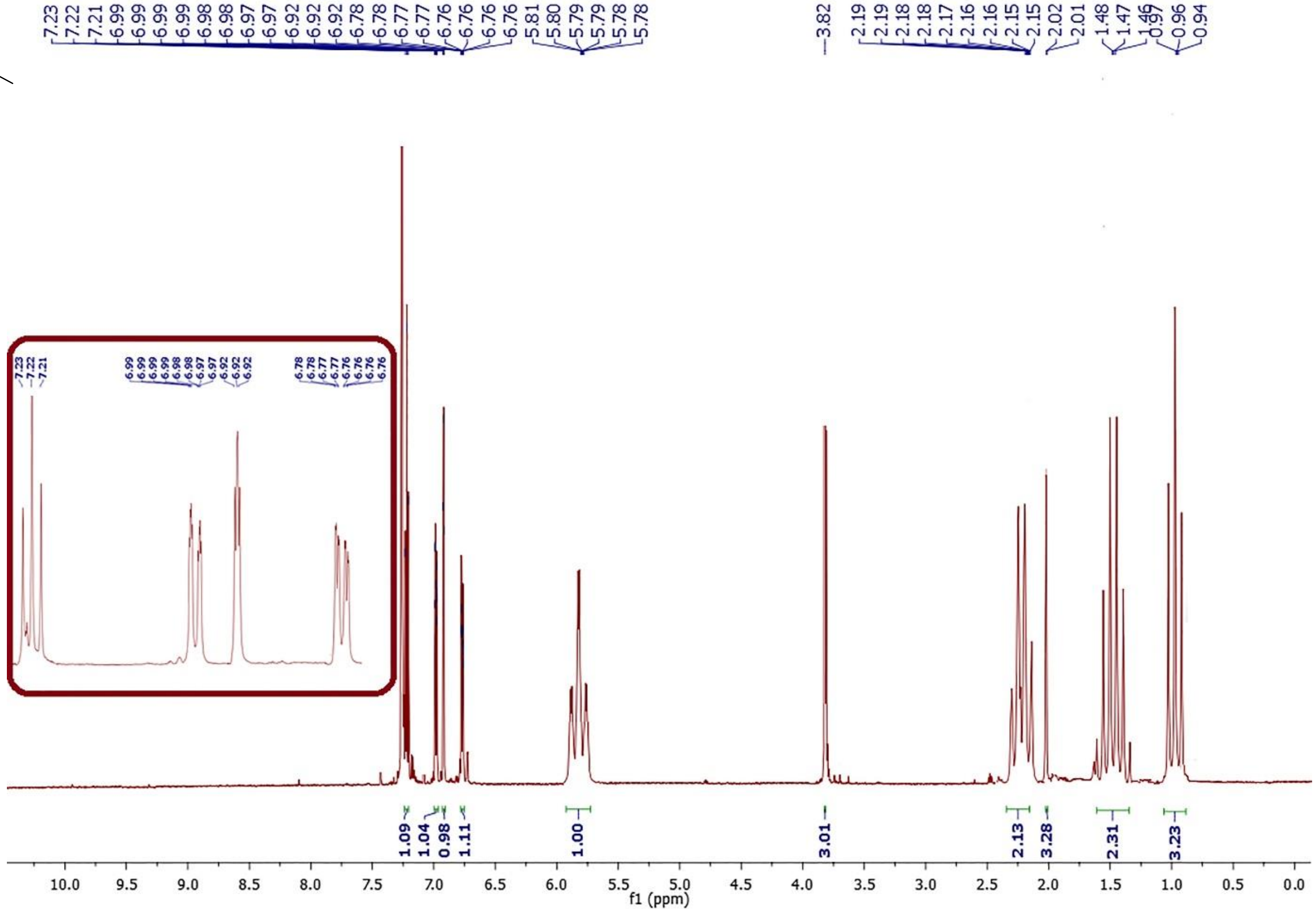

^{13}**C-APT (E)-1-(hex-2-en-2-yl)-3-methoxybenzene (2a)**

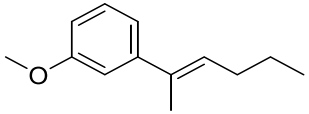

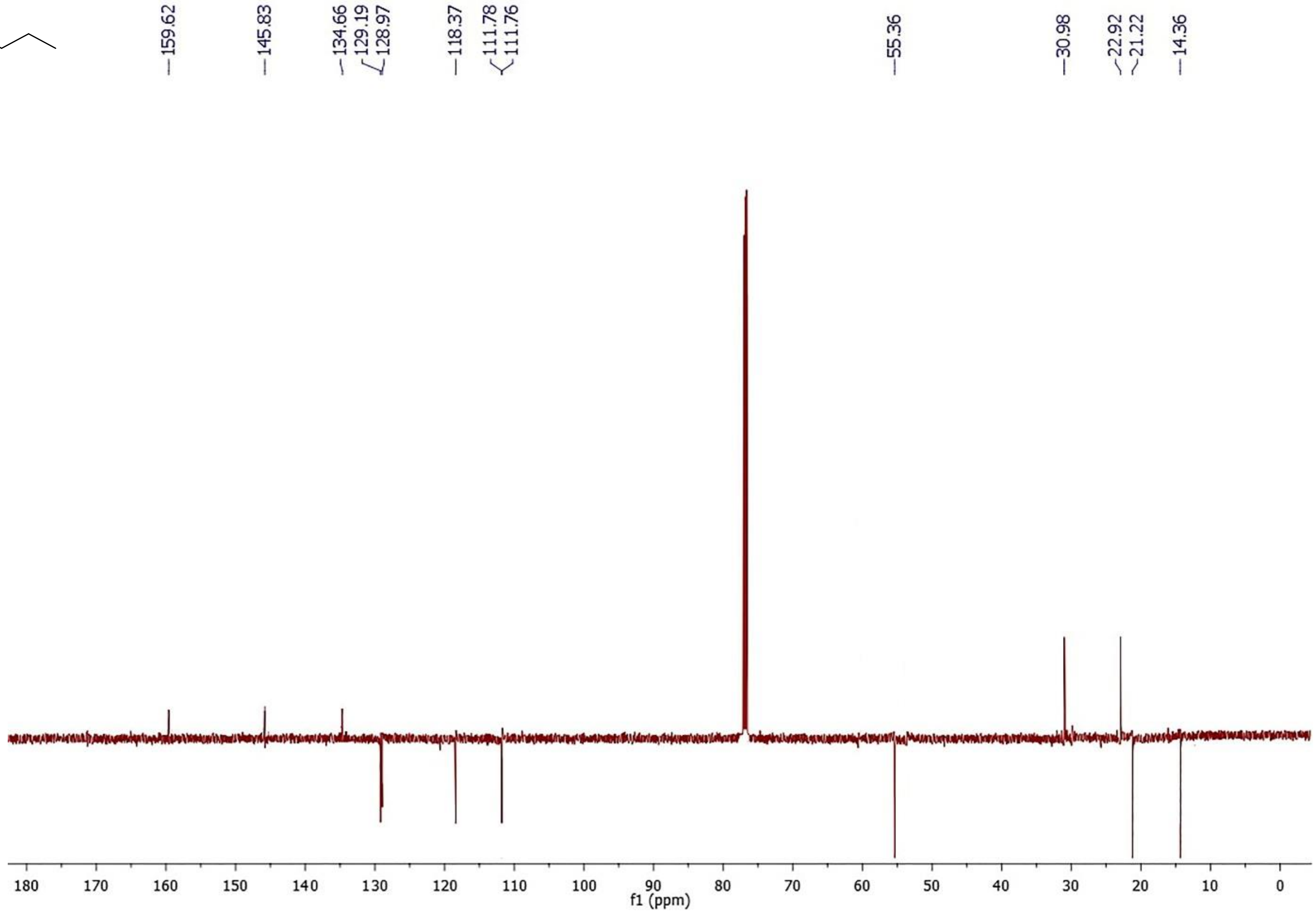

^{1}H-NMR (E)-1-methoxy-3-(oct-2-en-2-yl)benzene (2b)

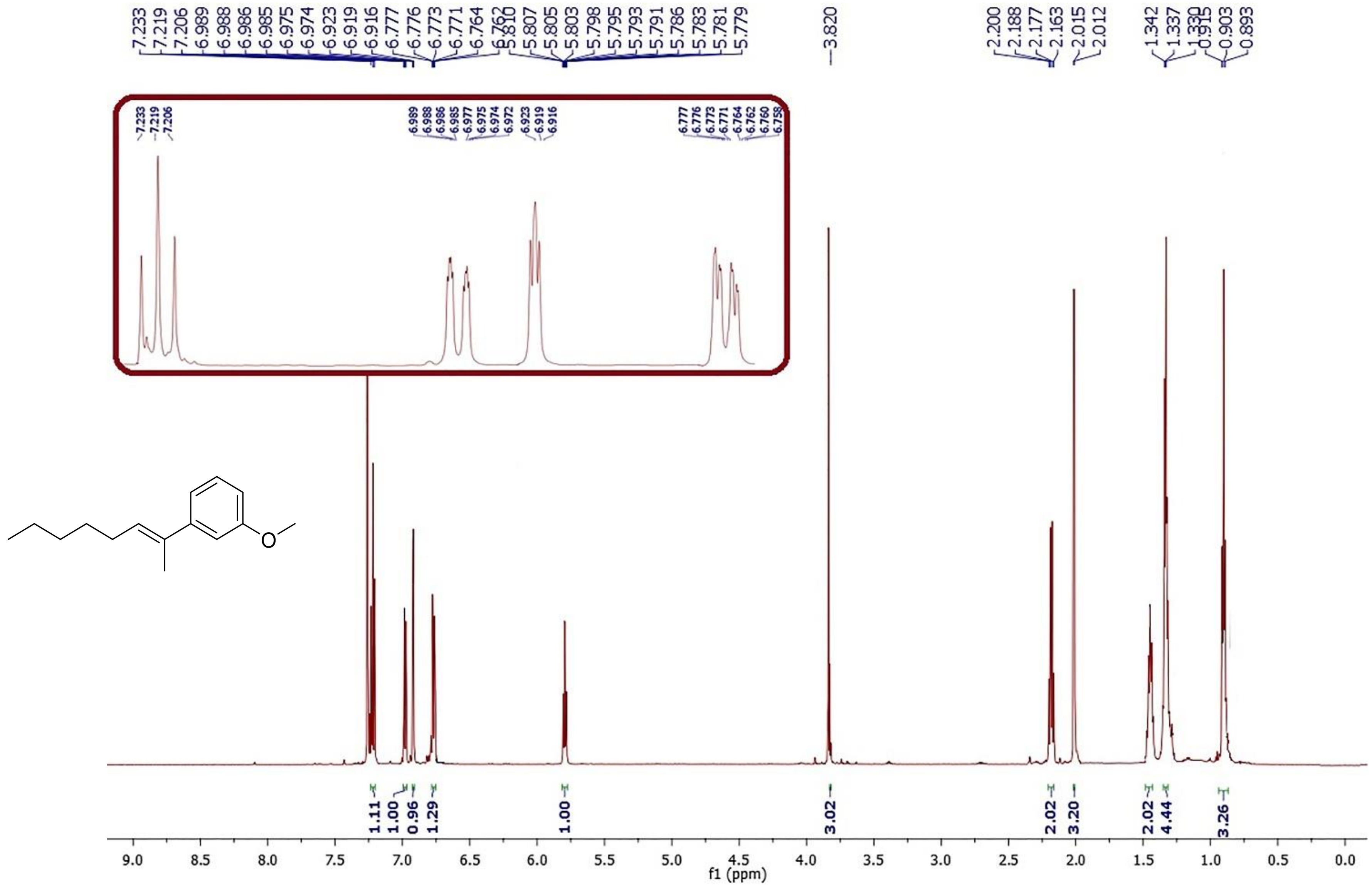

^{13}C-APT (E)-1-methoxy-3-(oct-2-en-2-yl)benzene (2b)

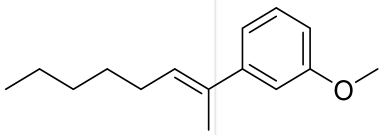

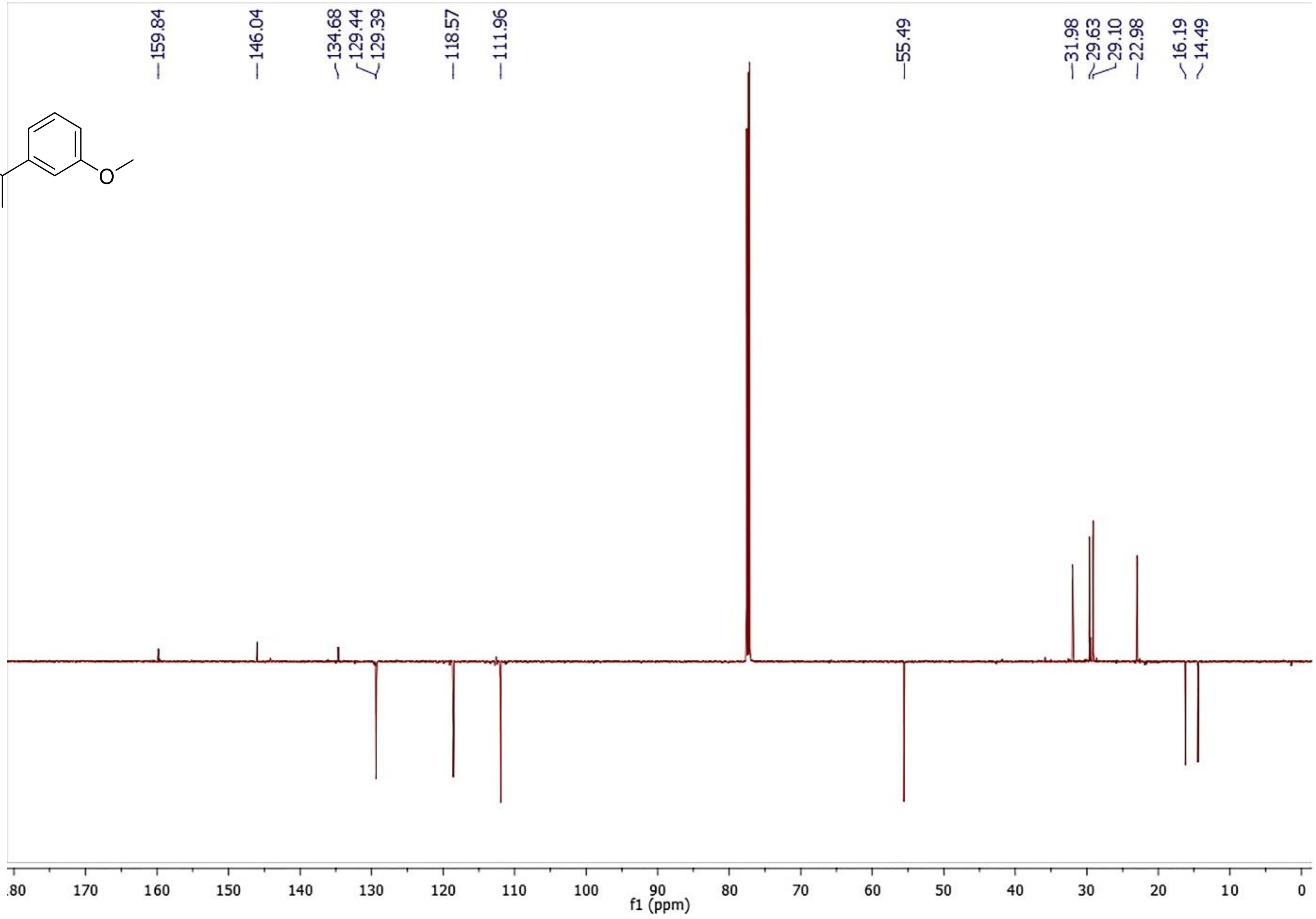

^{1}H-NMR (E)-1-(dodec-2-en-2-yl)-3-methoxybenzene (2c)

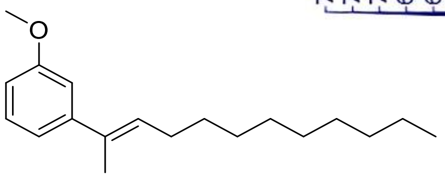

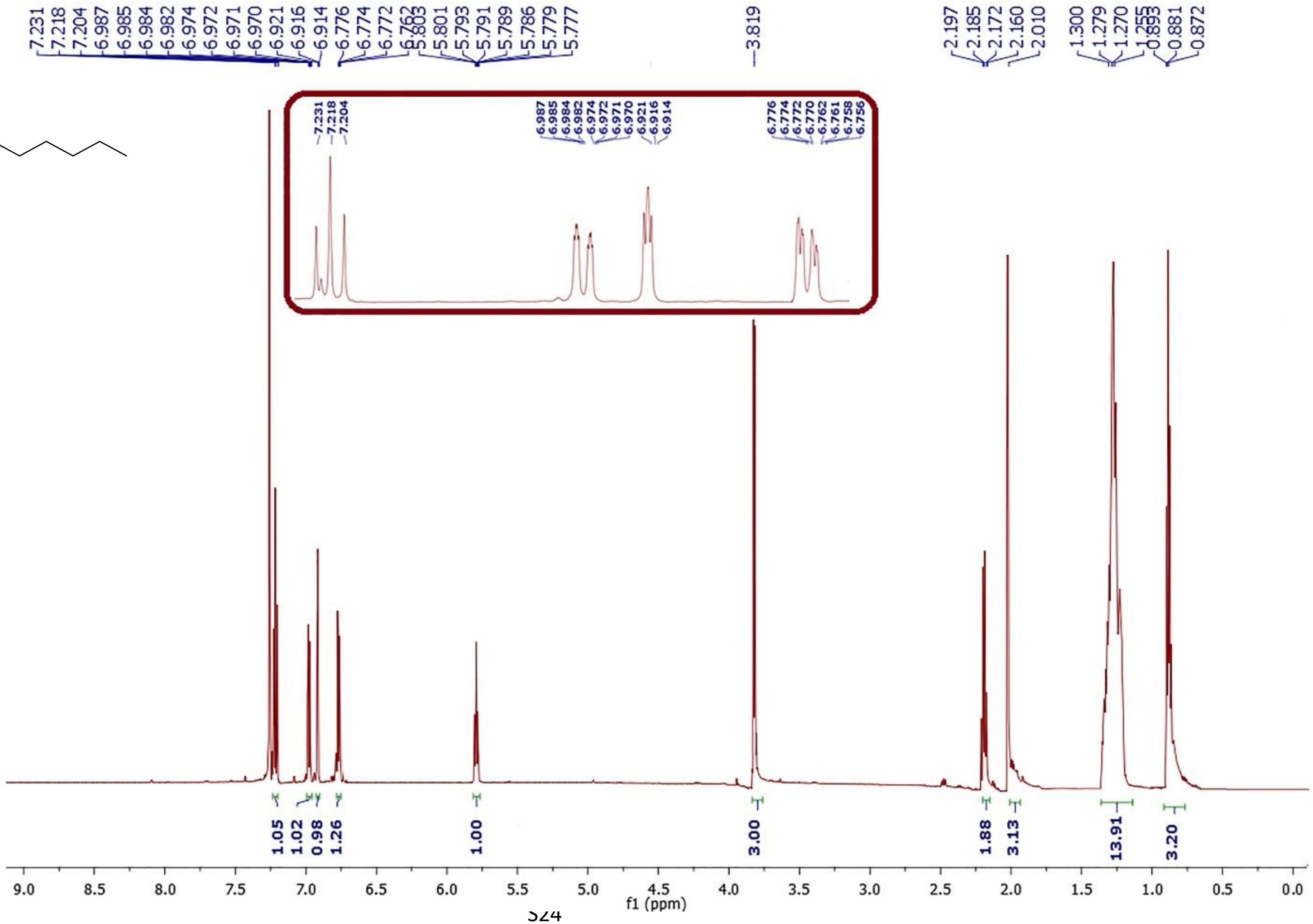

^{13}C-NMR (E)-1-(dodec-2-en-2-yl)-3-methoxybenzene (2c)

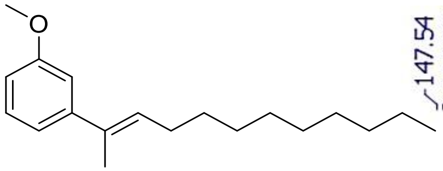

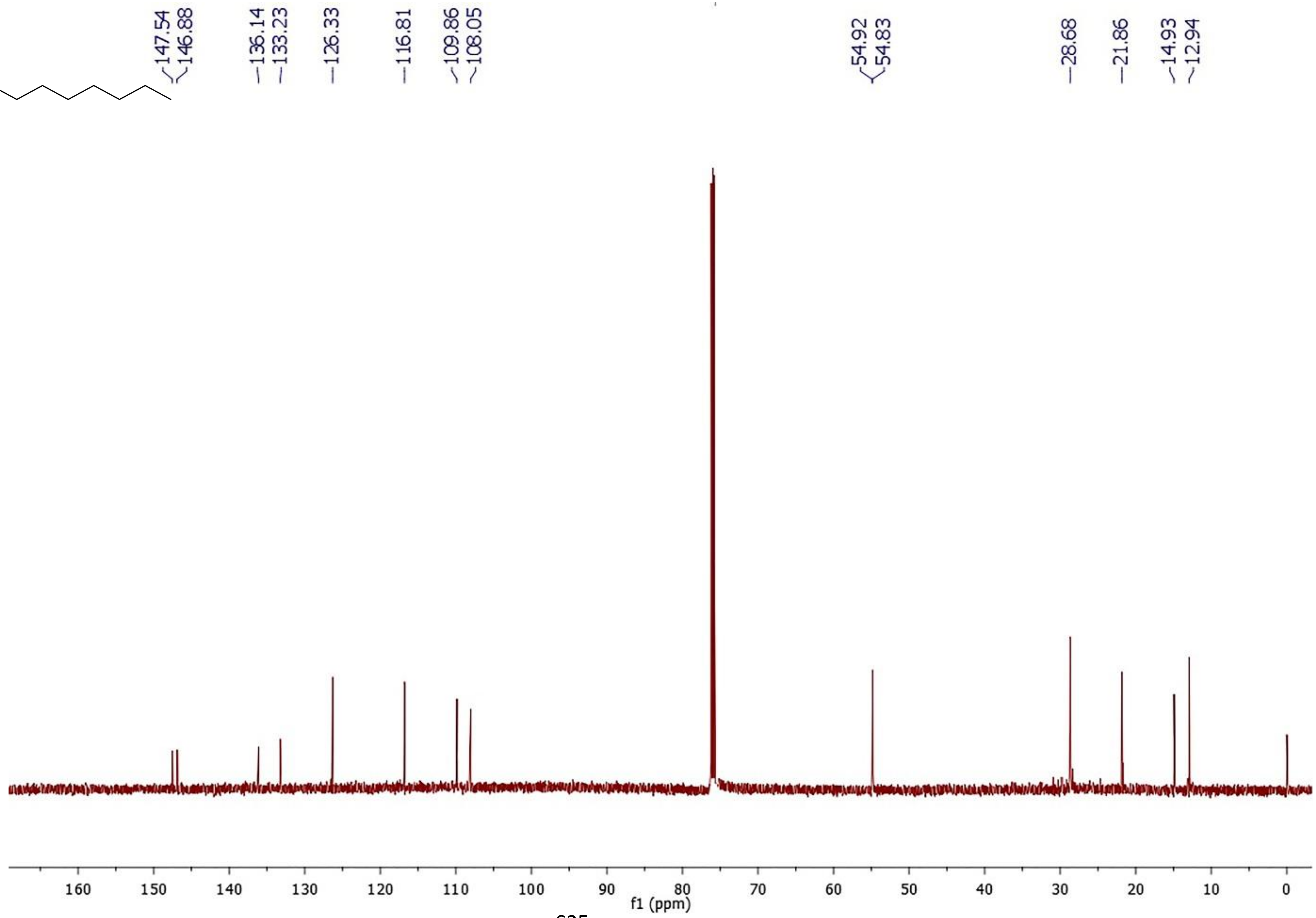

^{1}H-NMR (E)-1-ethoxy-3-(hex-2-en-2-yl)benzene (2d)

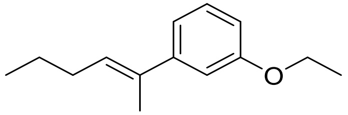

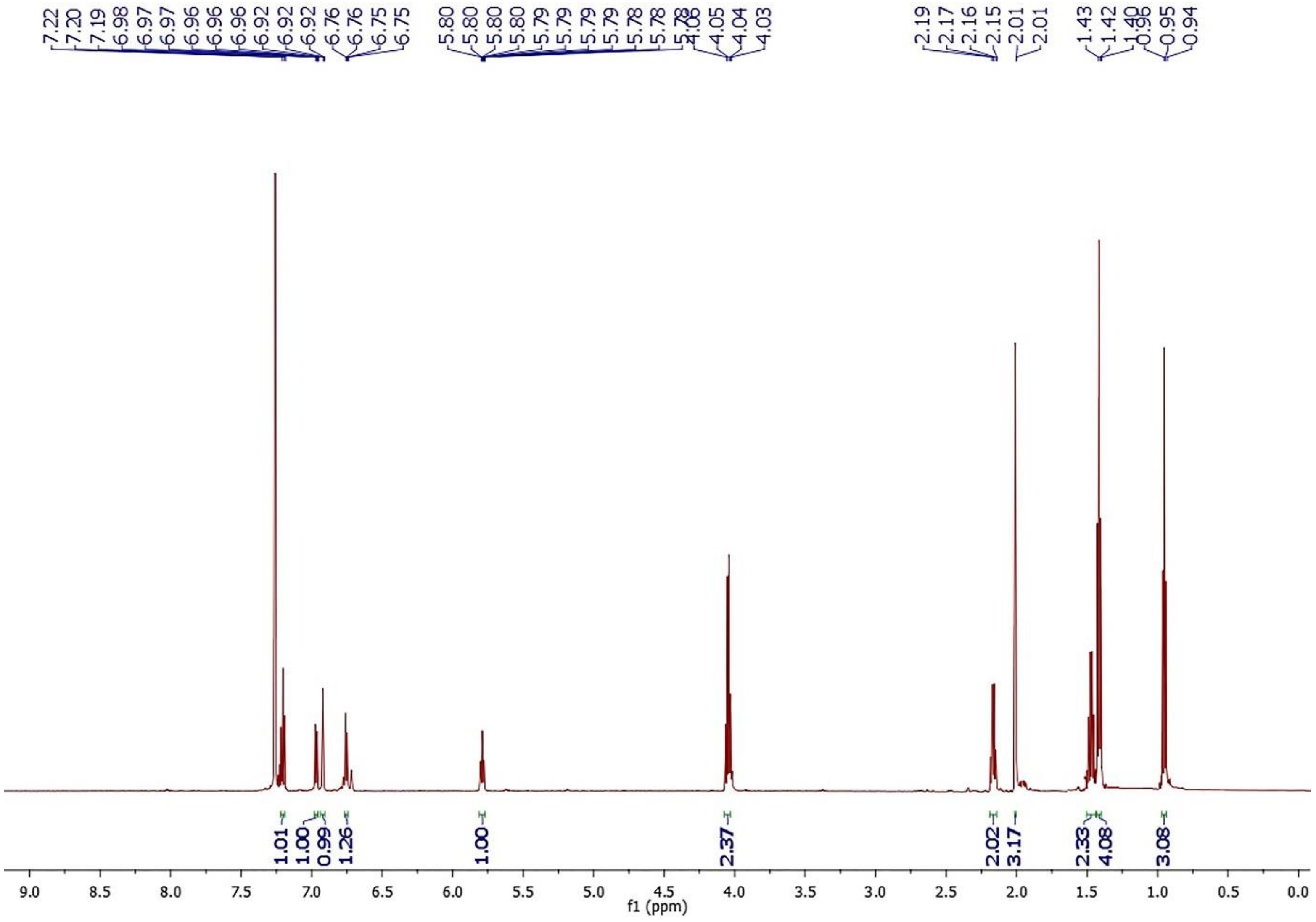

[13]C-APT (E)-1-ethoxy-3-(hex-2-en-2-yl)benzene (2d)

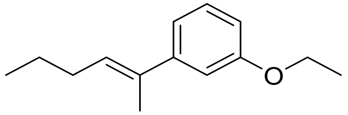

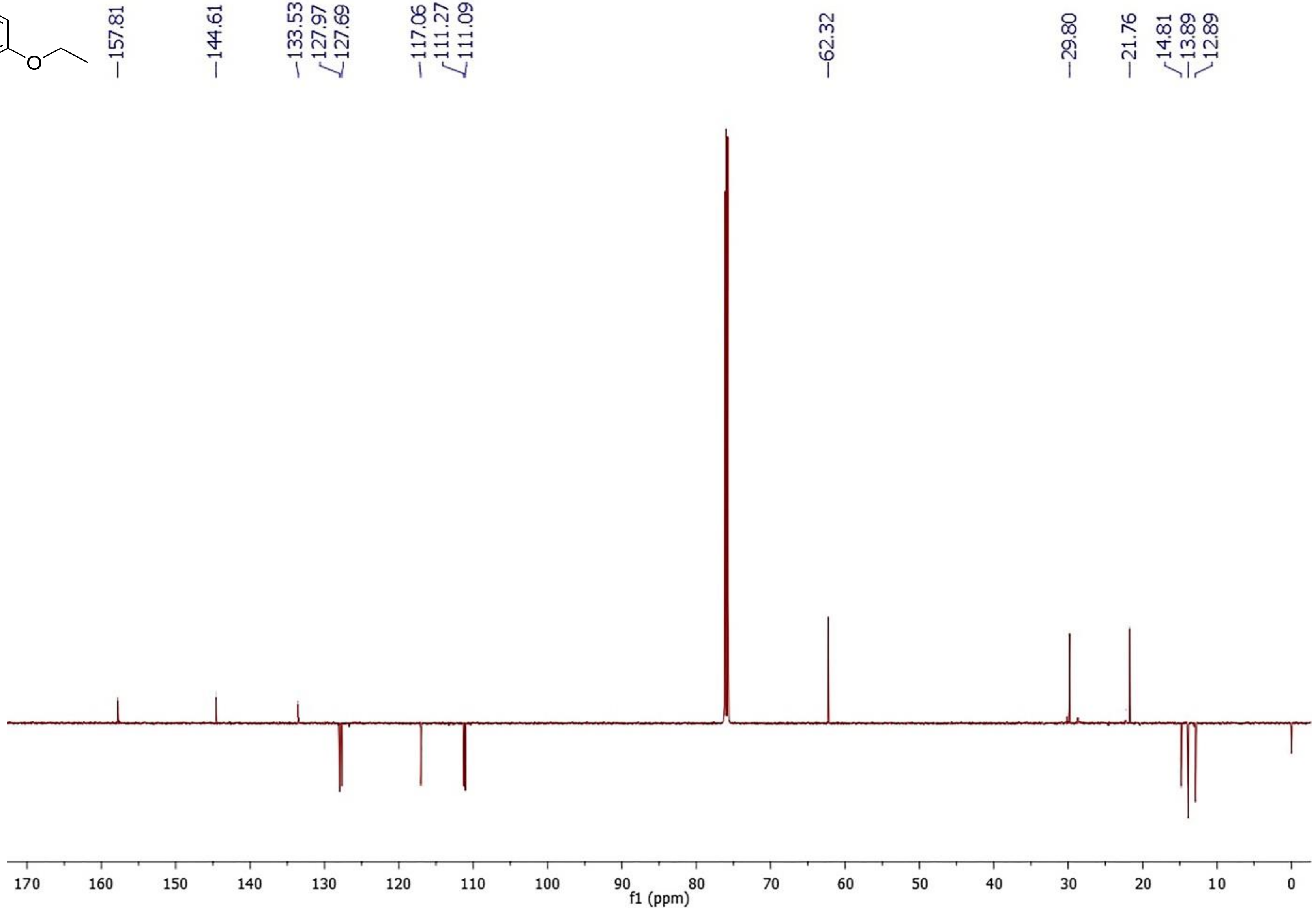

^{1}H-NMR (E)-1-ethoxy-3-(oct-2-en-2-yl)benzene (2e)

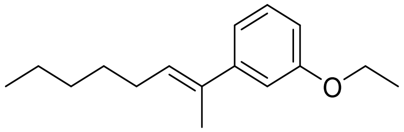

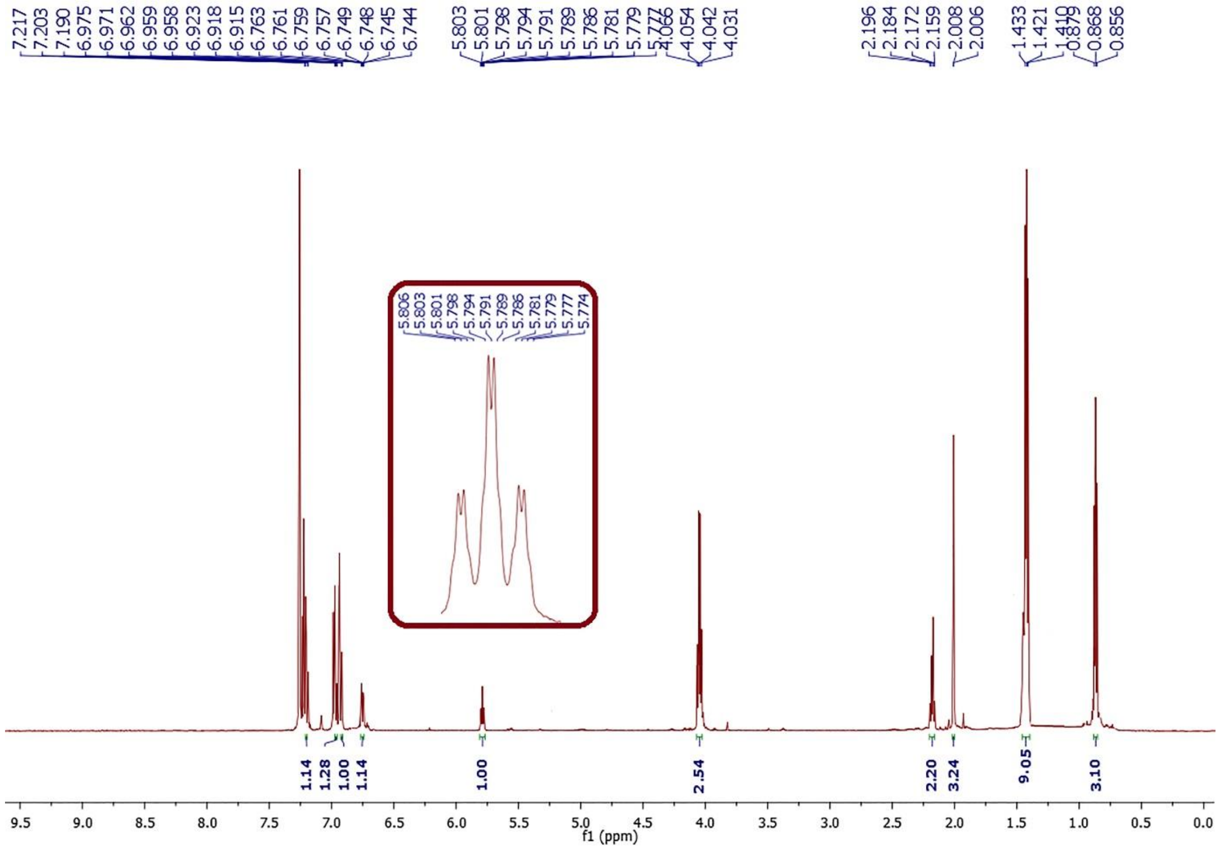

^{13}C-NMR (E)-1-ethoxy-3-(oct-2-en-2-yl)benzene (2e)

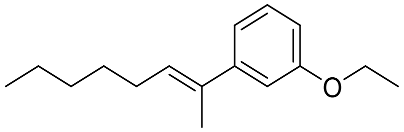

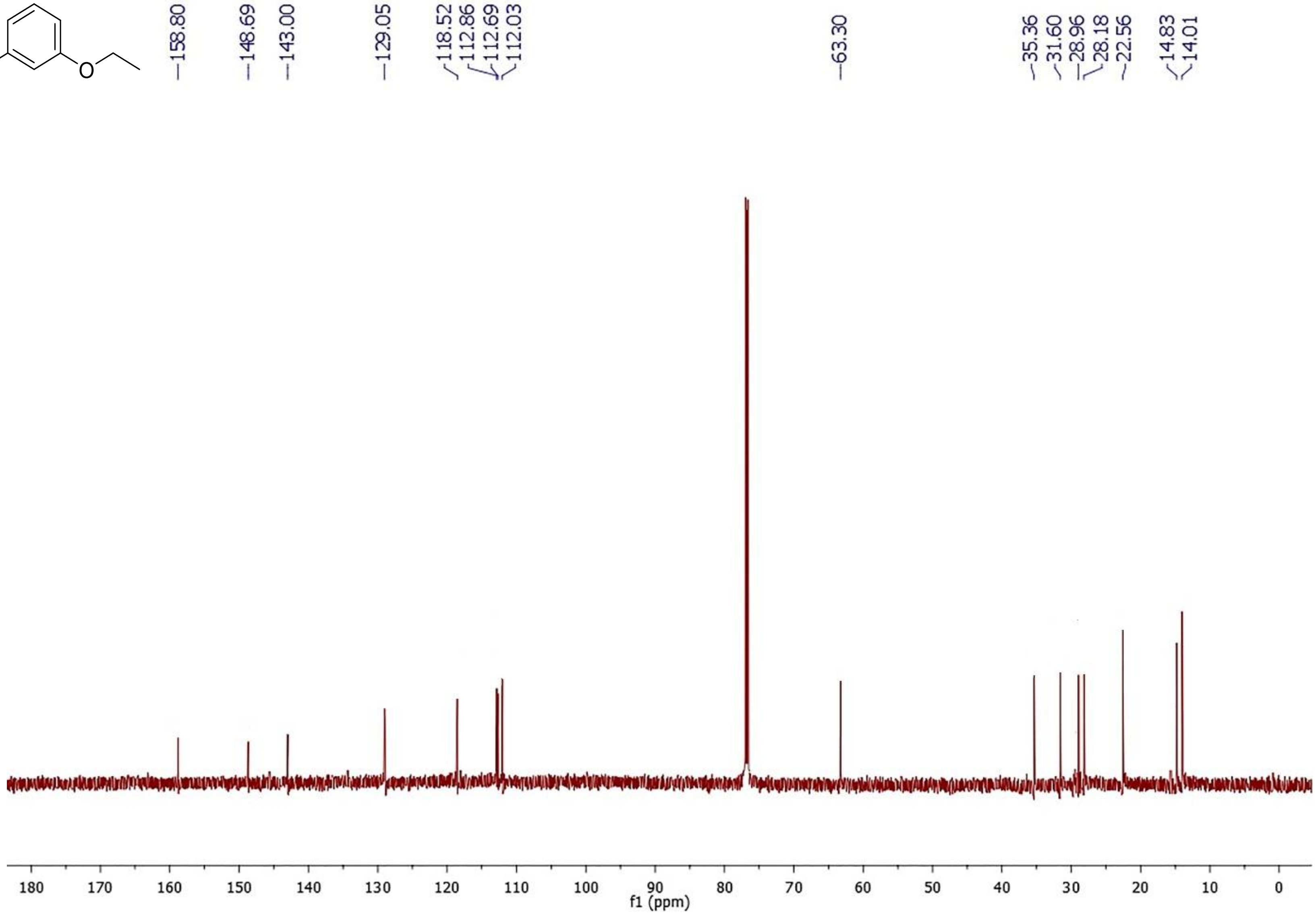

^{1}H-NMR (E)-1-(hex-2-en-2-yl)-3-methoxy-5-methylbenzene (2f)

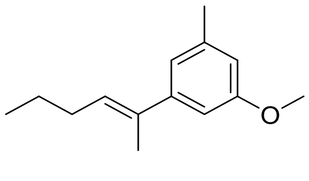

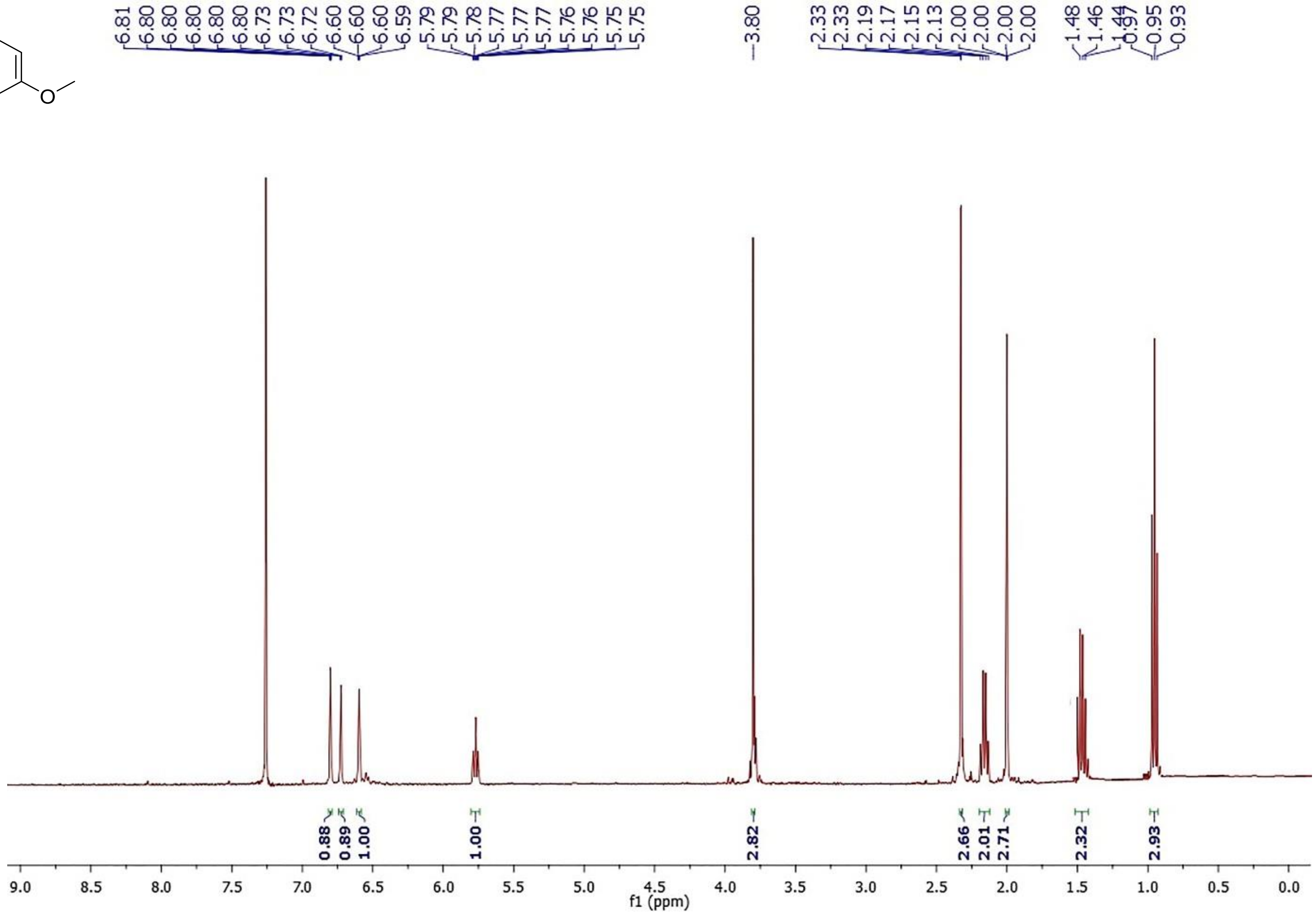

^{13}C-NMR (E)-1-(hex-2-en-2-yl)-3-methoxy-5-methylbenzene (2f)

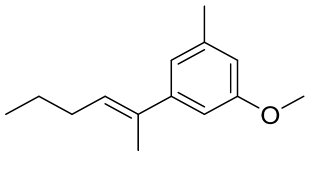

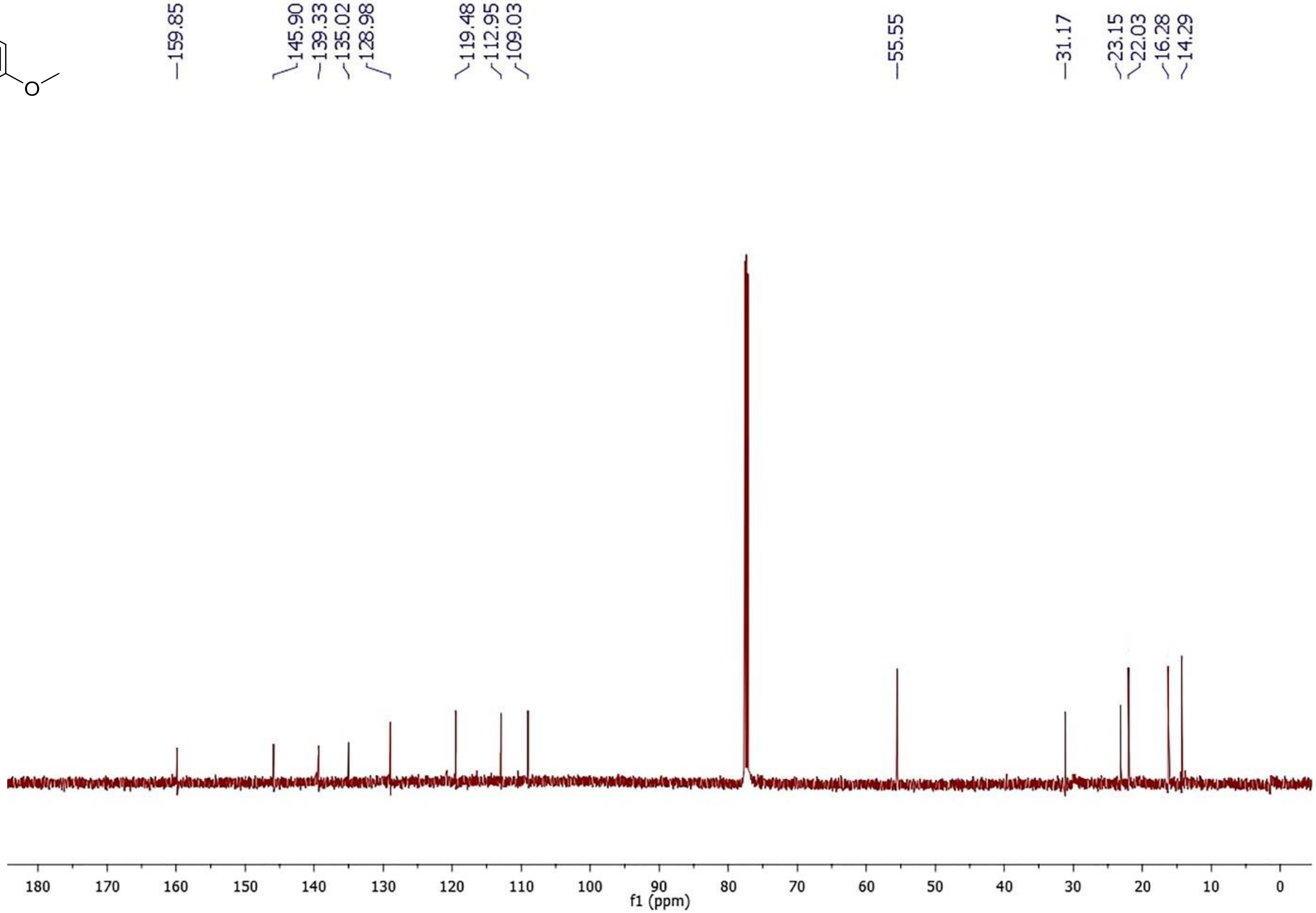

^{1}H-NMR (E)-1-(hept-2-en-2-yl)-3-methoxy-5-methylbenzene (2g)

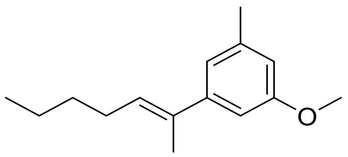

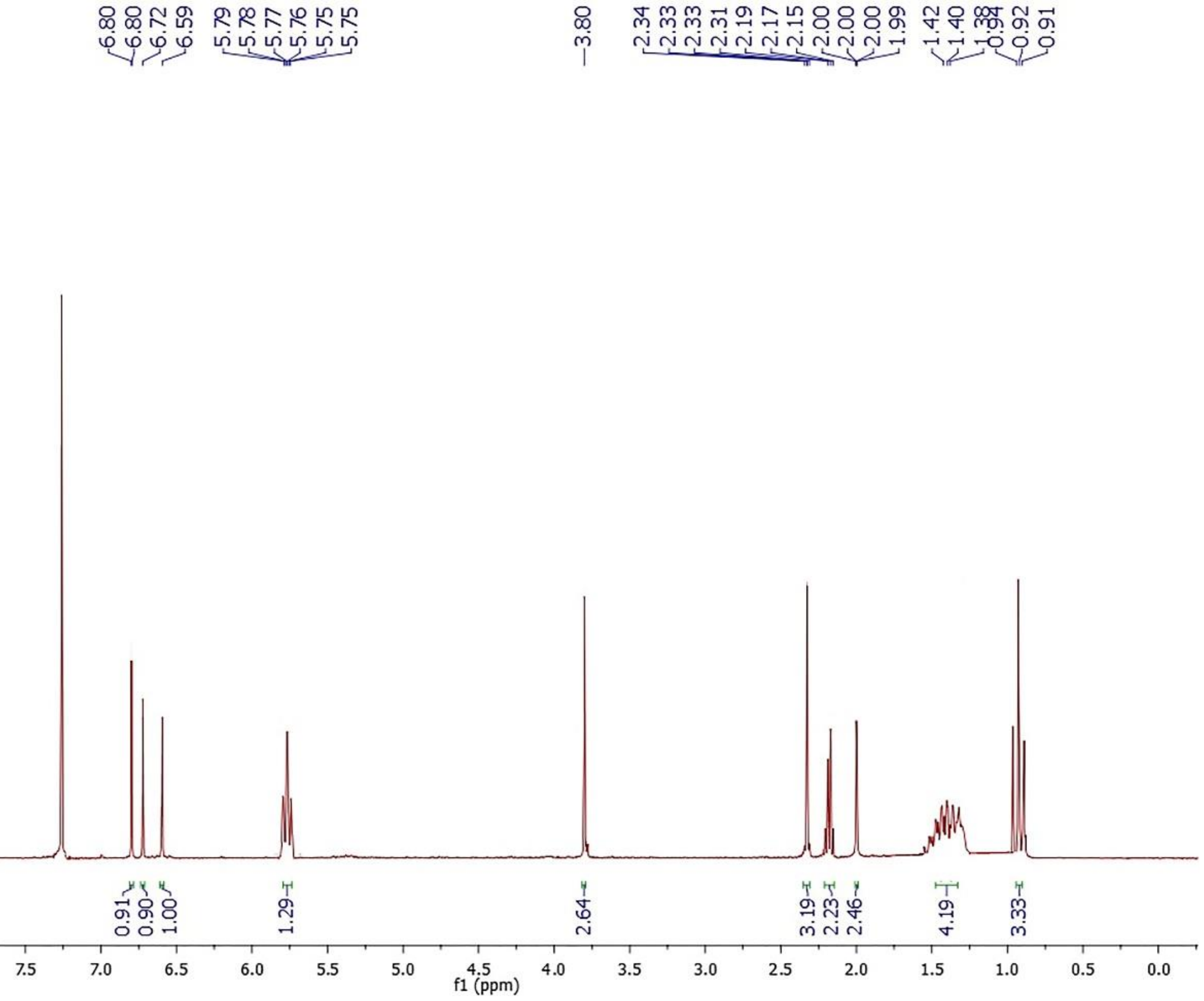

^{13}C-NMR (E)-1-(hept-2-en-2-yl)-3-methoxy-5-methylbenzene (2g)

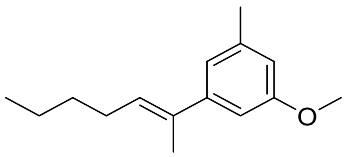

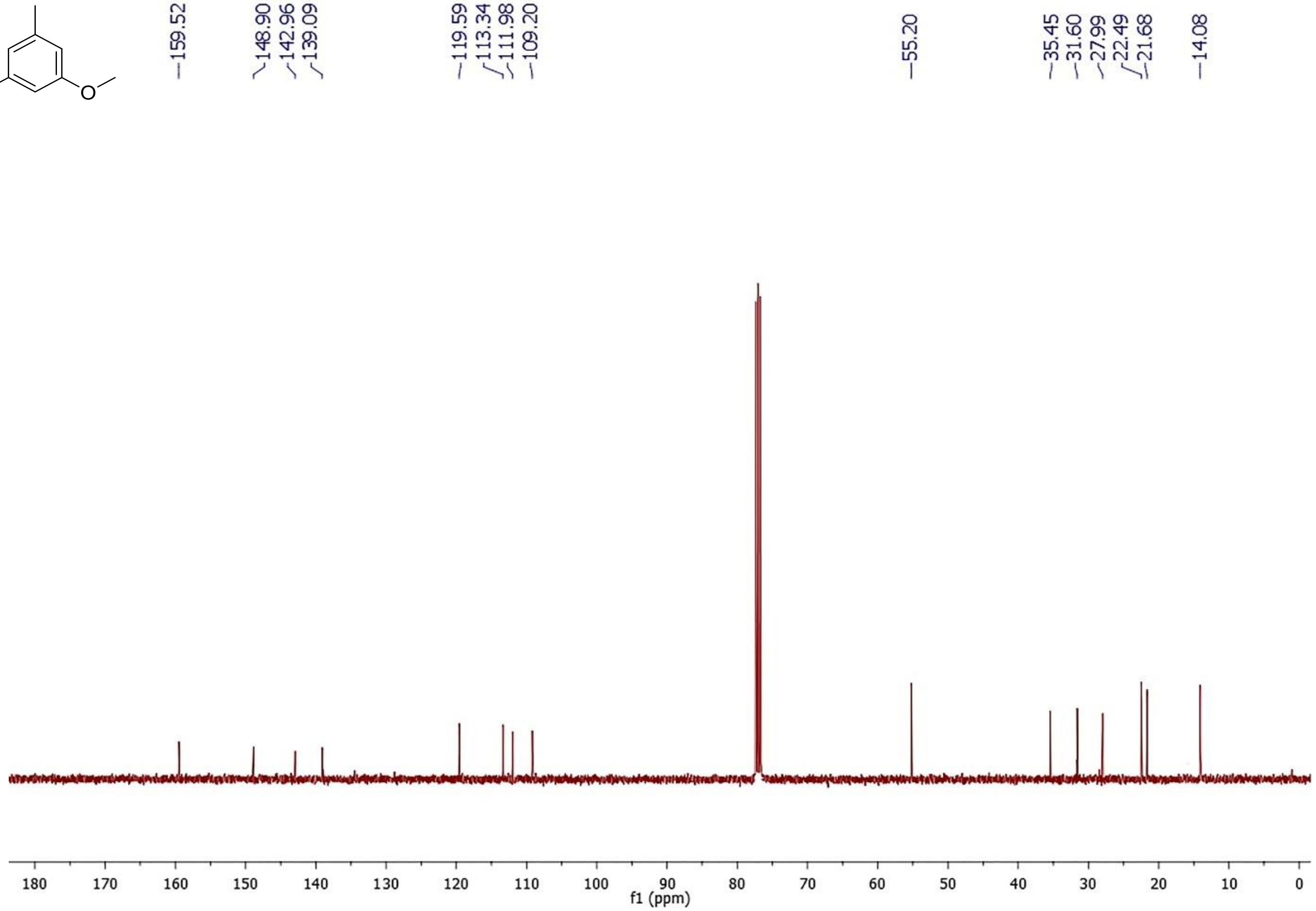

^{1}H-NMR (E)-1-methoxy-3-methyl-5-(oct-2-en-2-yl)benzene (2h)

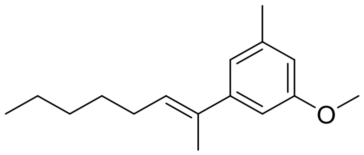

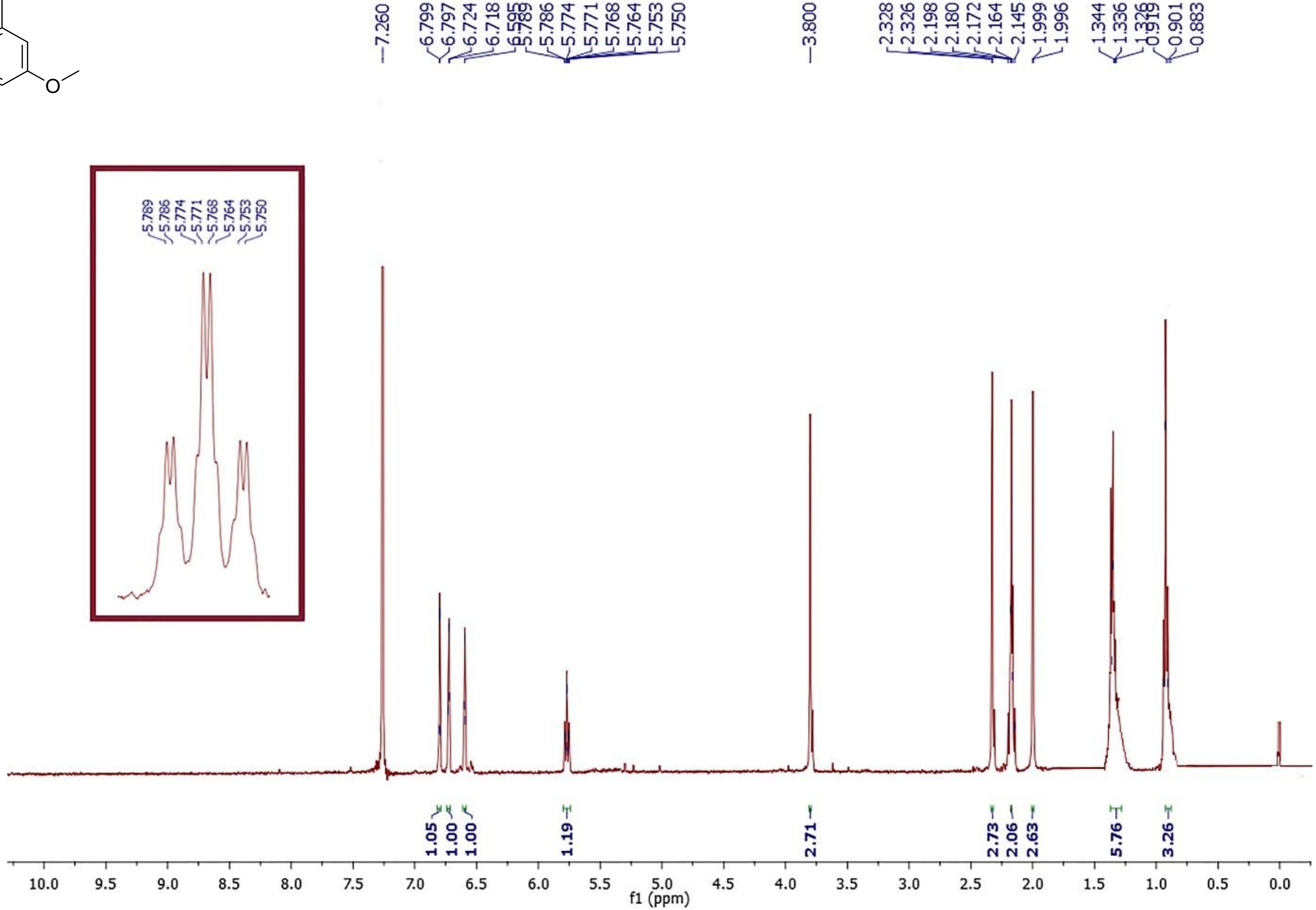

^{13}C-NMR (E)-1-methoxy-3-methyl-5-(oct-2-en-2-yl)benzene (2h)

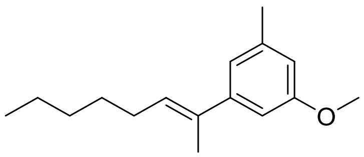

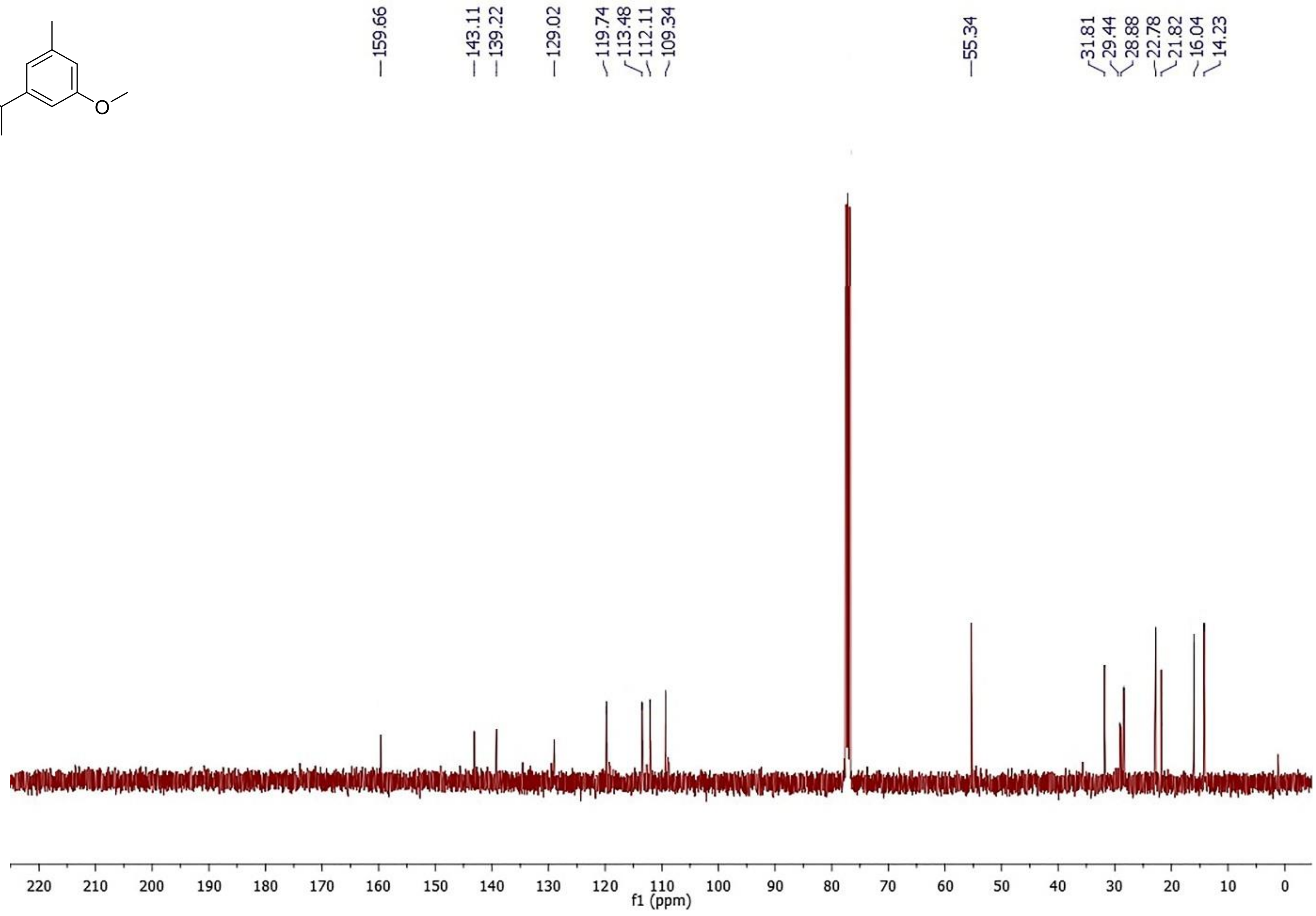

^{1}H-NMR (E)-1-methoxy-3-methyl-5-(4-methylhex-2-en-2-yl)benzene (2i)

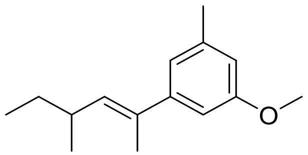

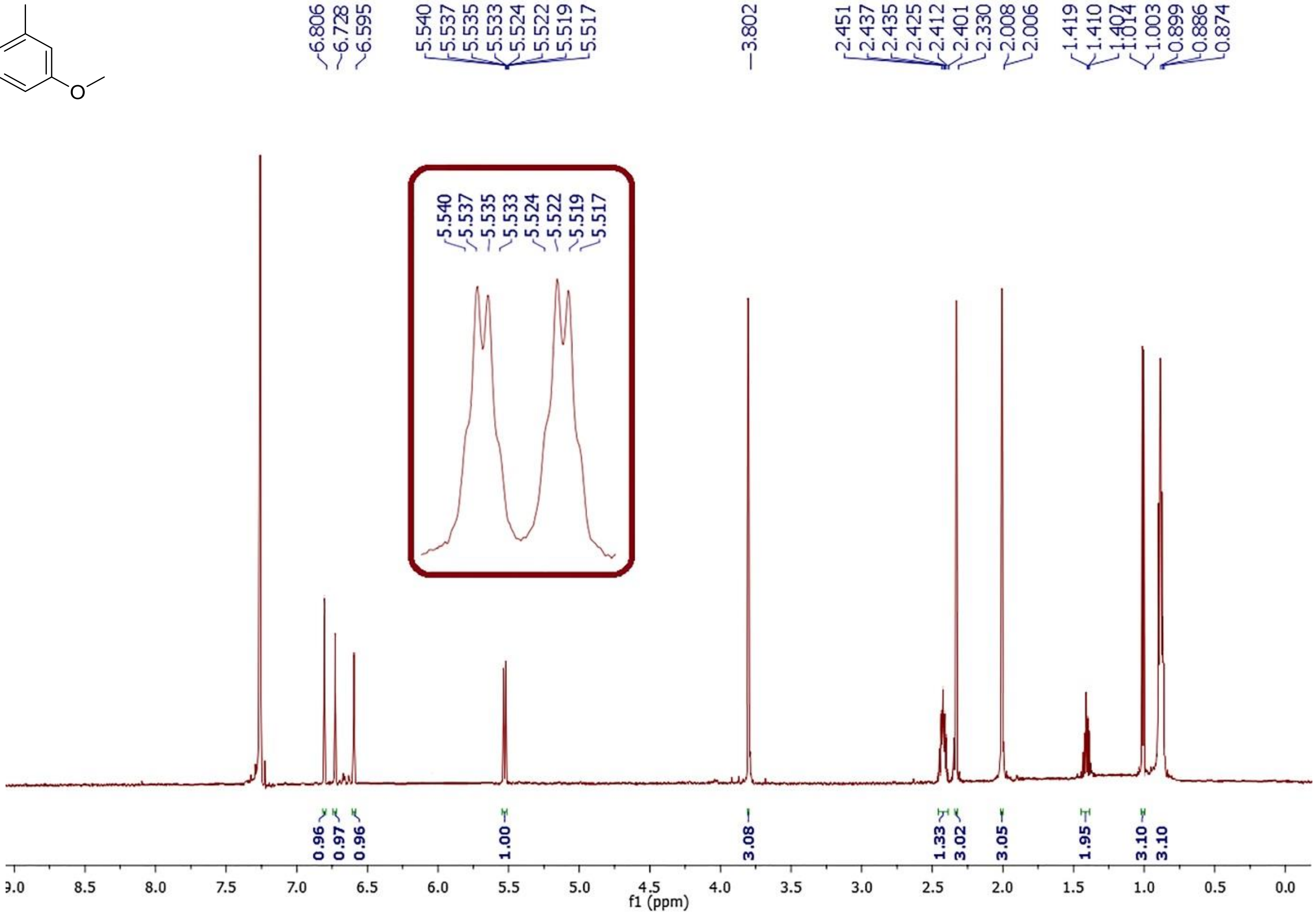

[13]C-APT (E)-1-methoxy-3-methyl-5-(4-methylhex-2-en-2-yl)benzene (2i)

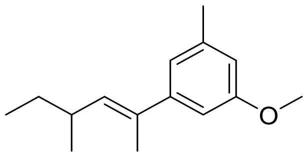

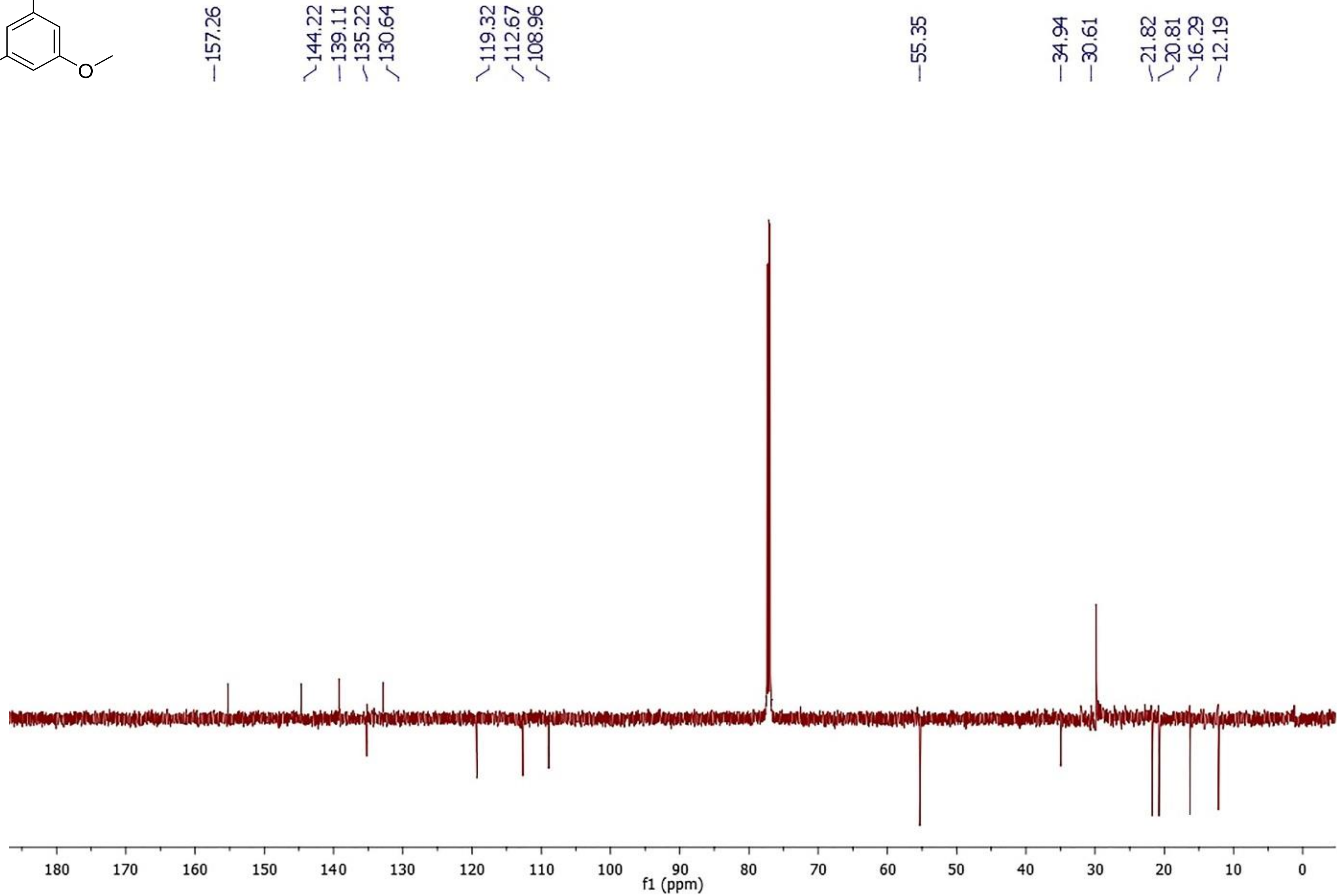

^{1}H-NMR (E)-4-(hex-2-en-2-yl)-1,2-dimethoxybenzene (2j)

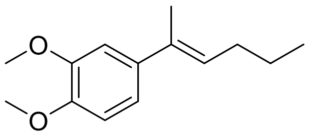

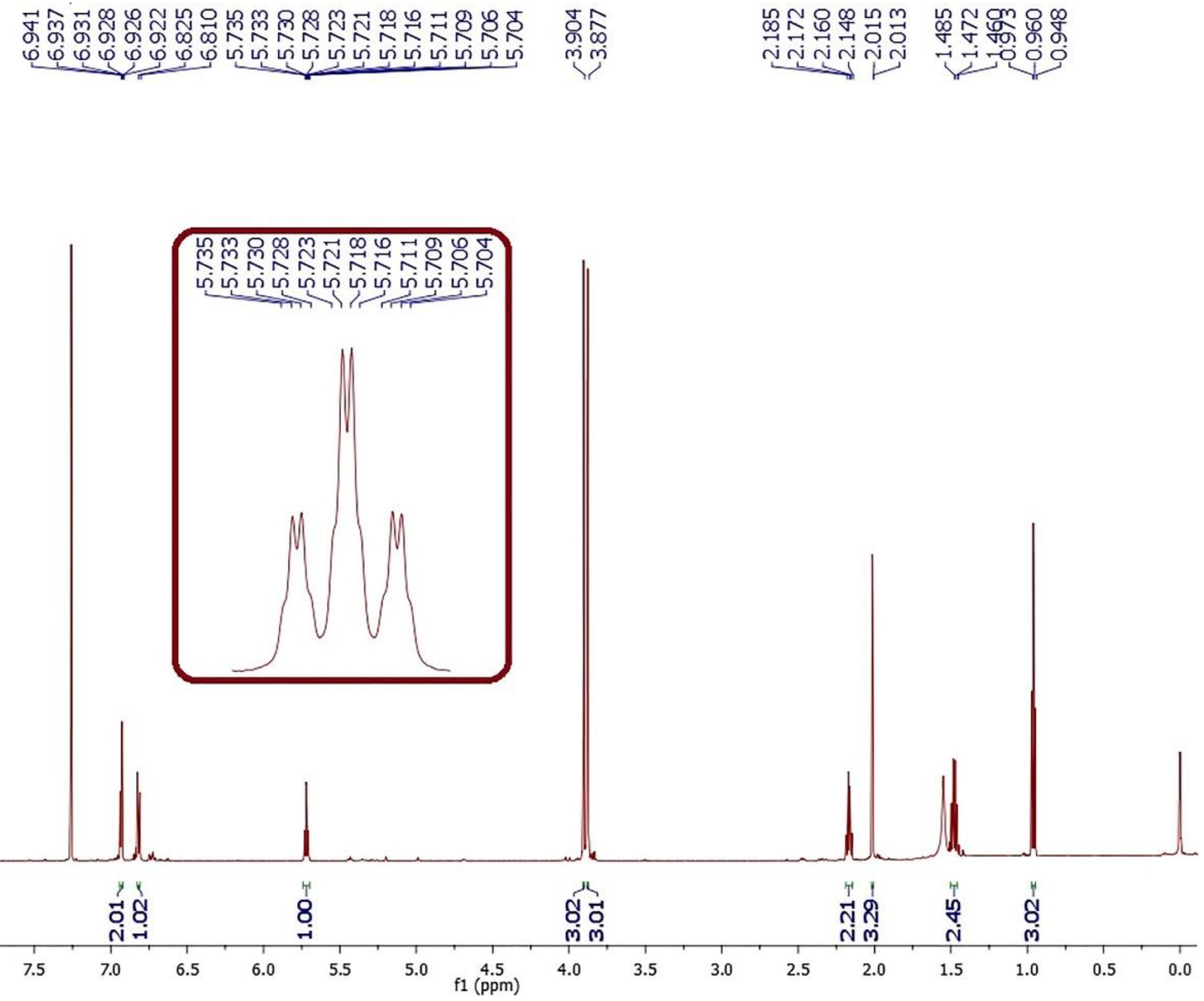

^{13}C-NMR (E)-4-(hex-2-en-2-yl)-1,2-dimethoxybenzene (2j)

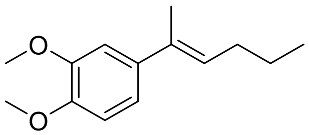

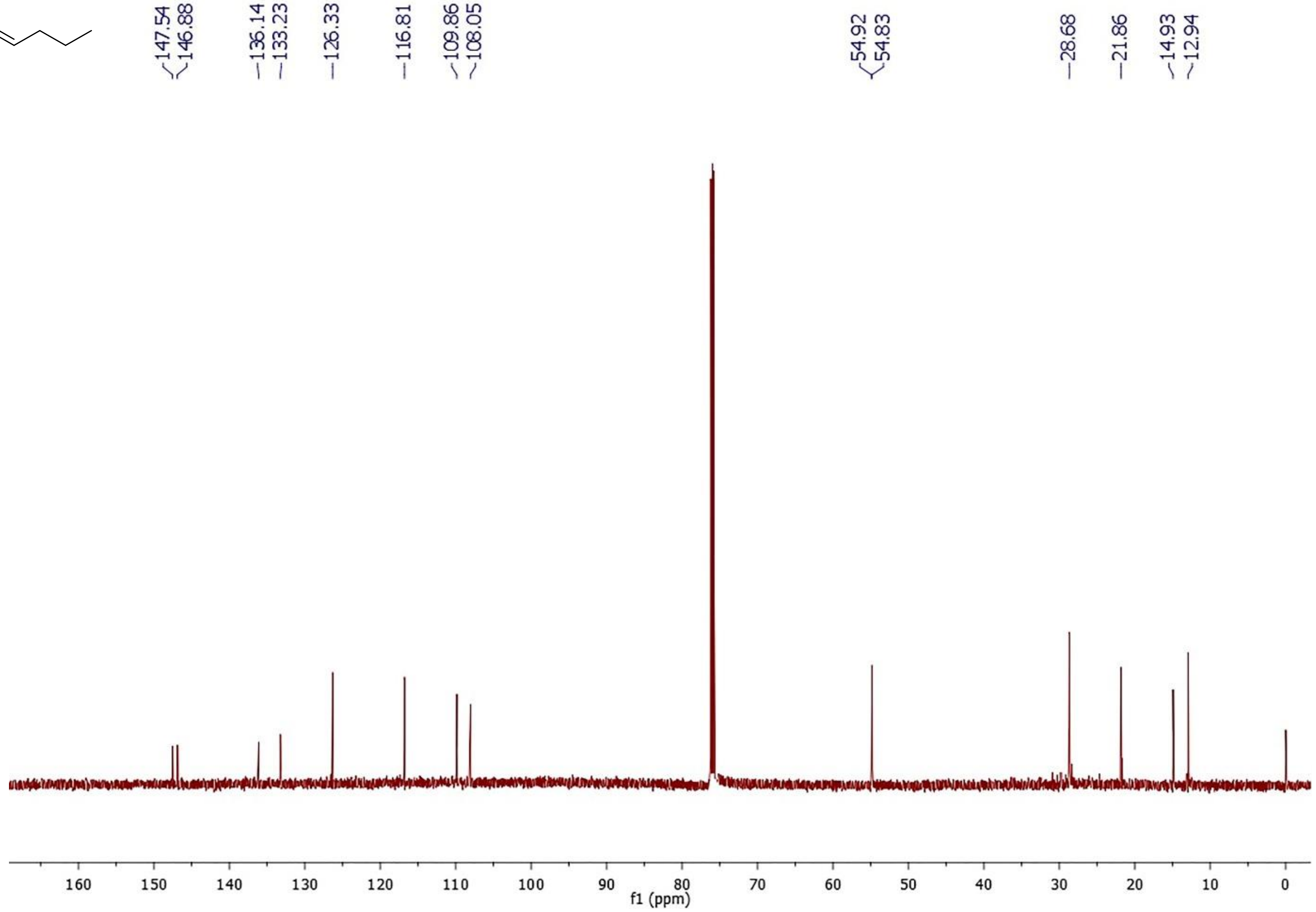

^{1}H-NMR (E)-4-(hept-2-en-2-yl)-1,2-dimethoxybenzene (2k)

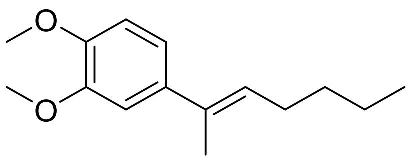

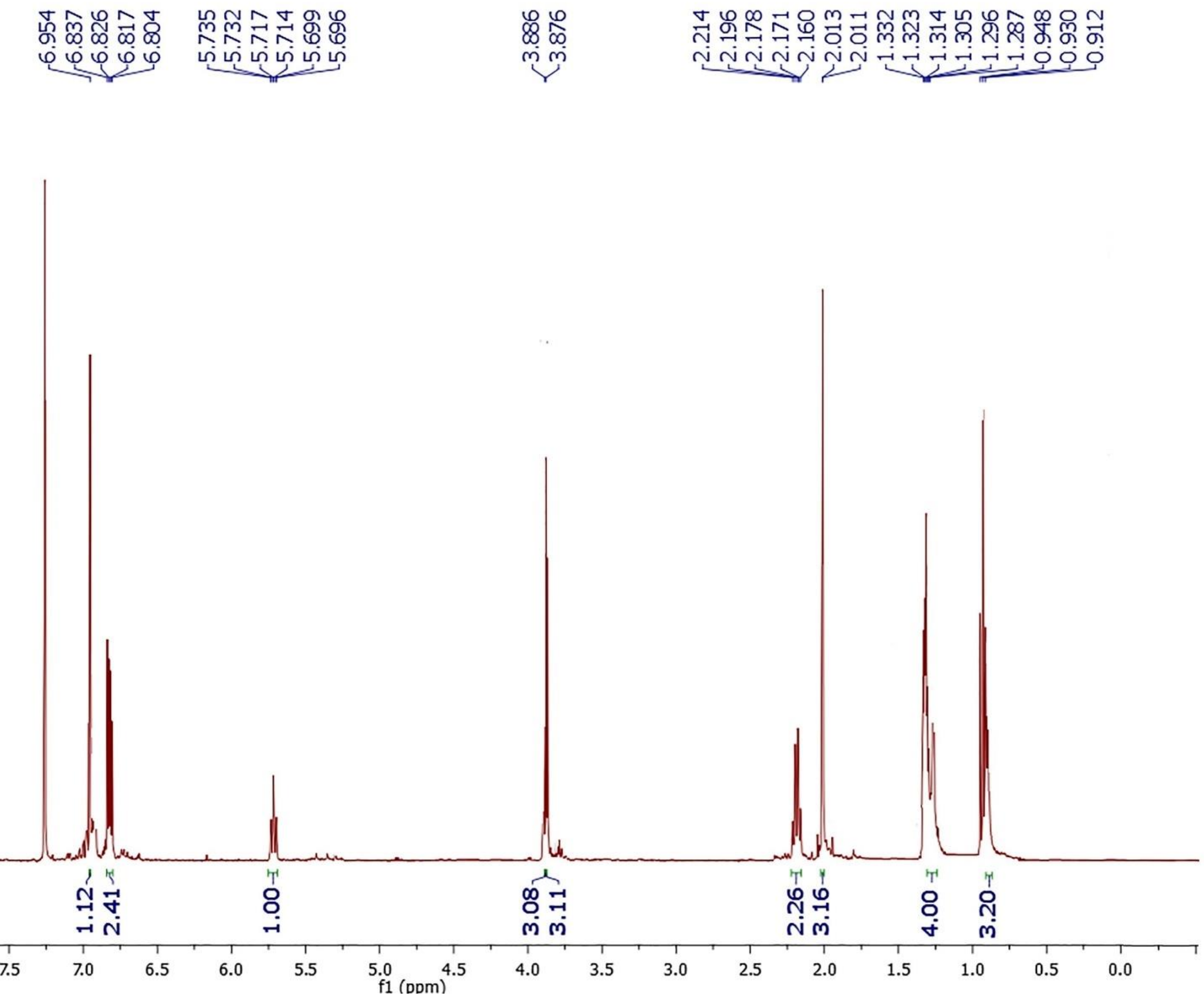

^{13}C-NMR (E)-4-(hept-2-en-2-yl)-1,2-dimethoxybenzene (2k)

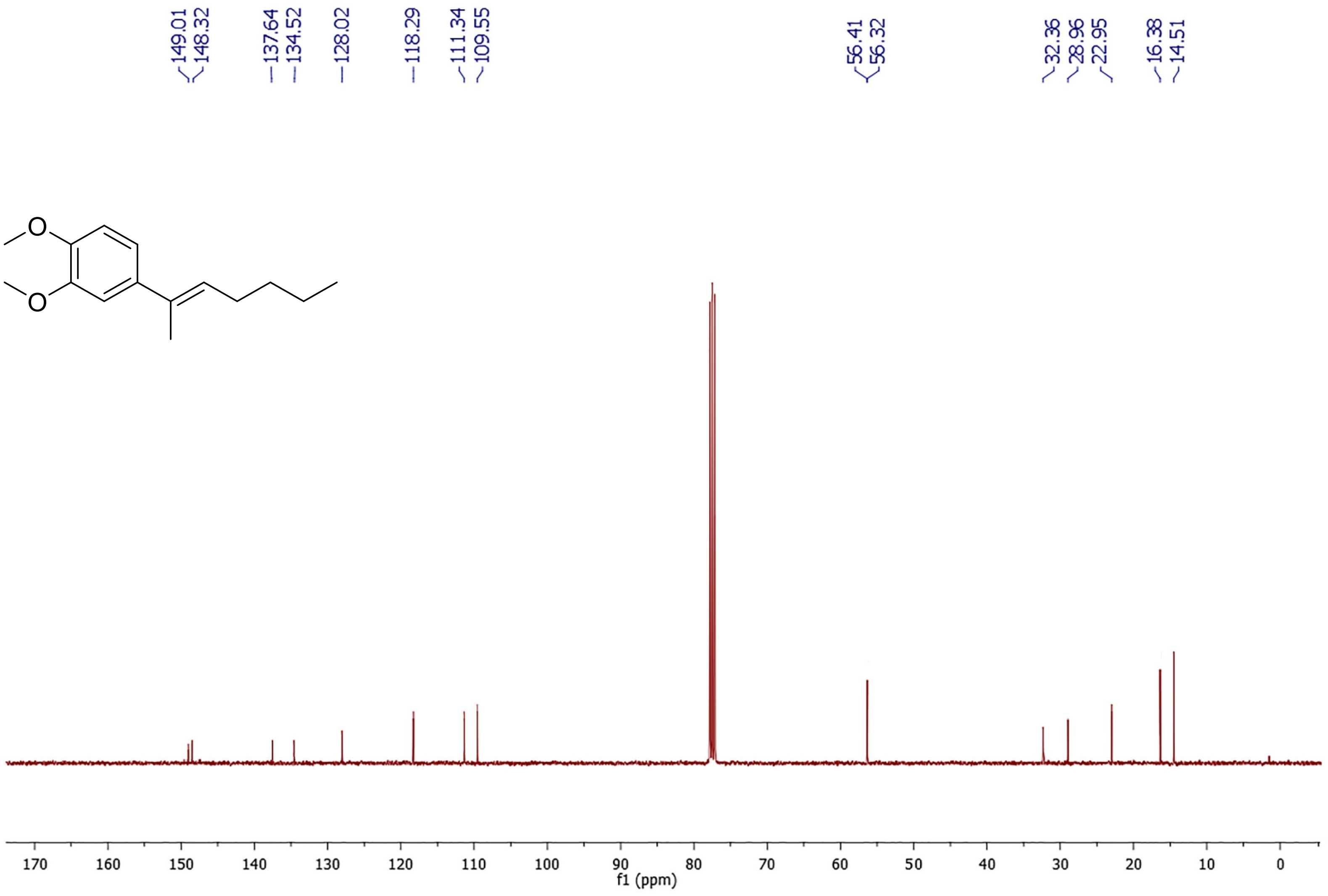

^{1}H-NMR (E)-1,2-dimethoxy-4-(oct-2-en-2-yl)benzene (2l)

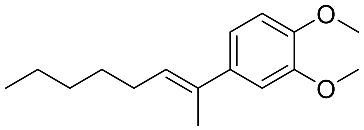

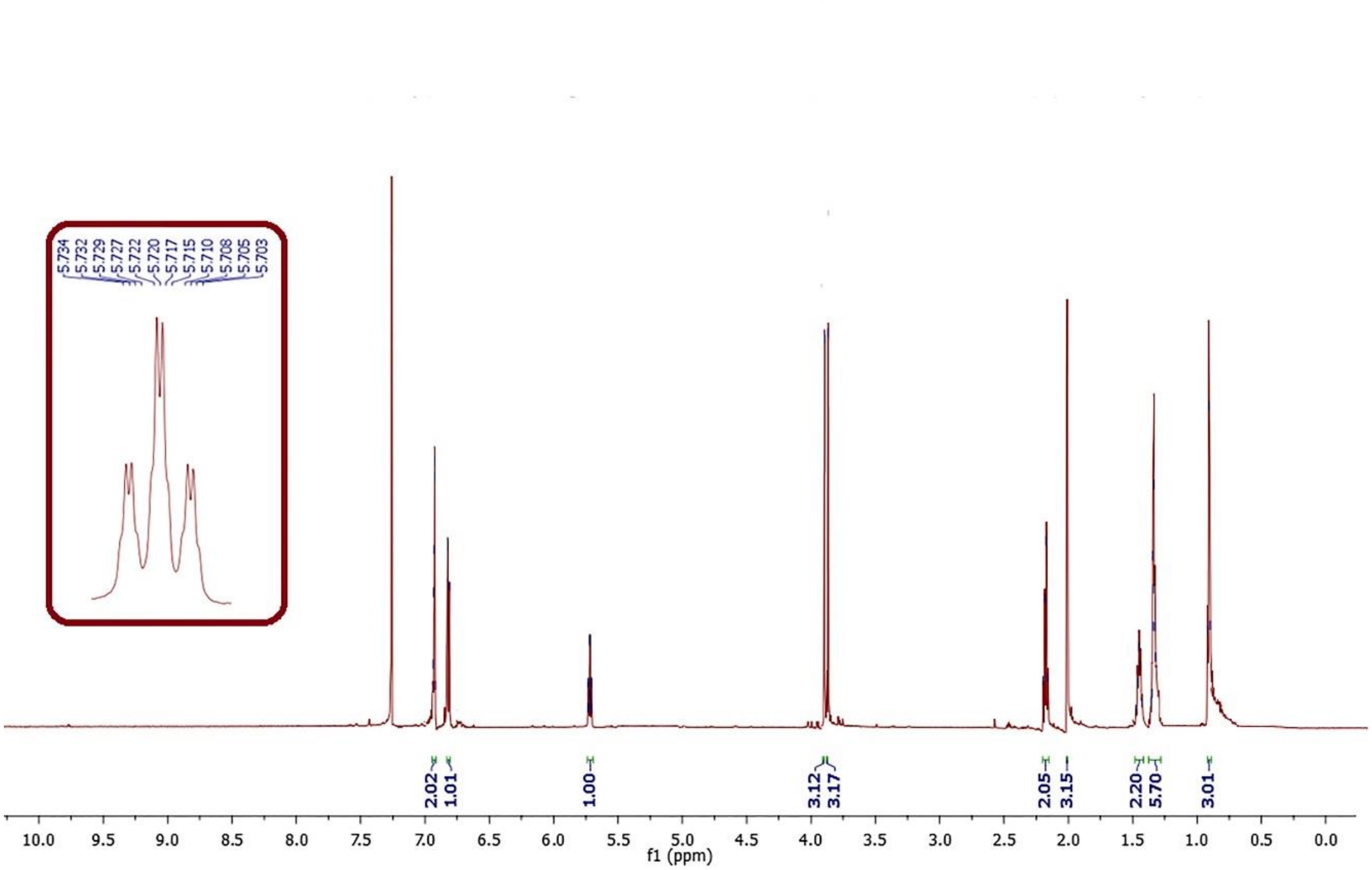

^{13}C-NMR (E)-1,2-dimethoxy-4-(oct-2-en-2-yl)benzene (2l)

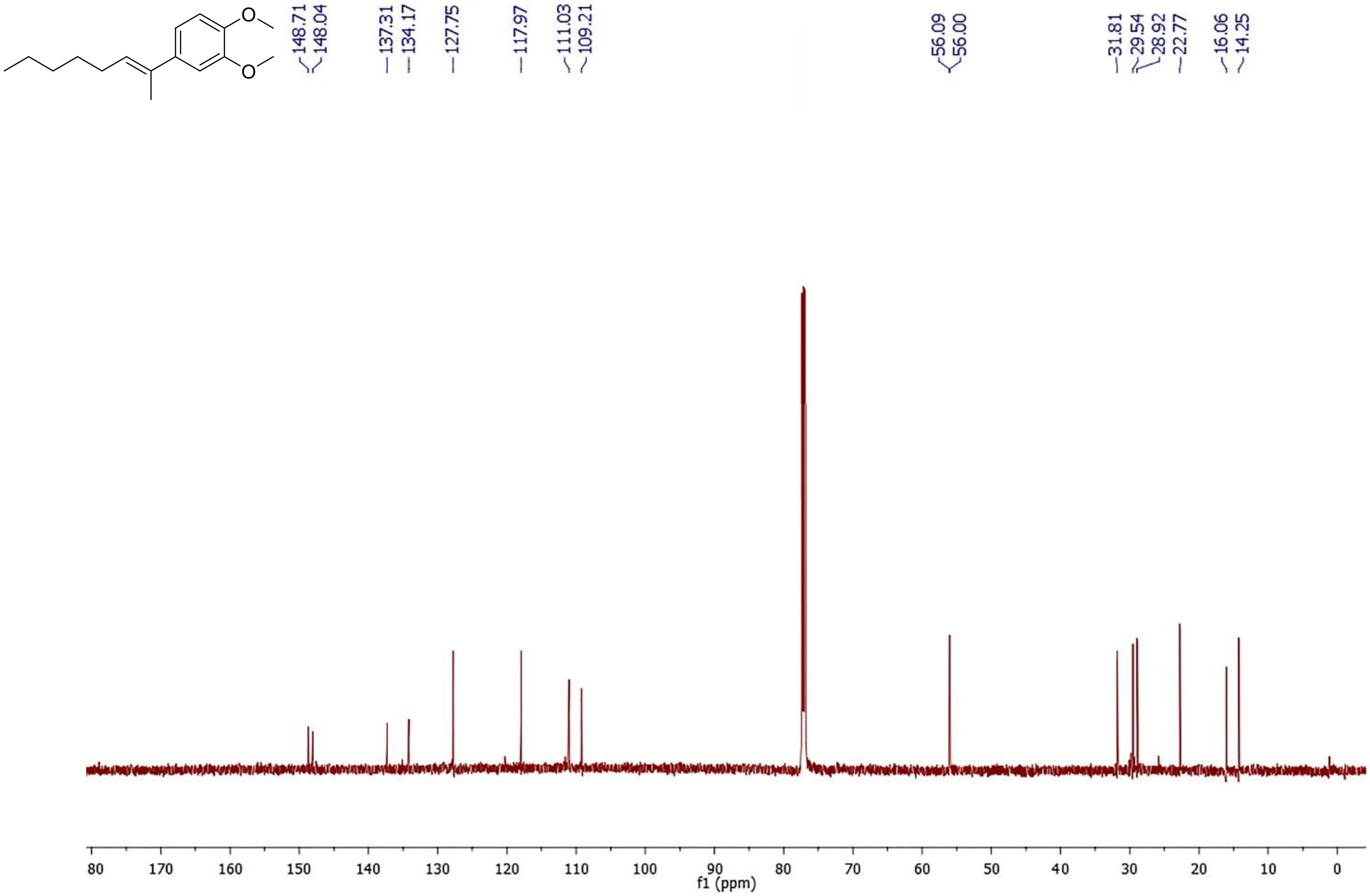

^{1}H-NMR (E)-4-(dodec-2-en-2-yl)-1,2-dimethoxybenzene (2m)

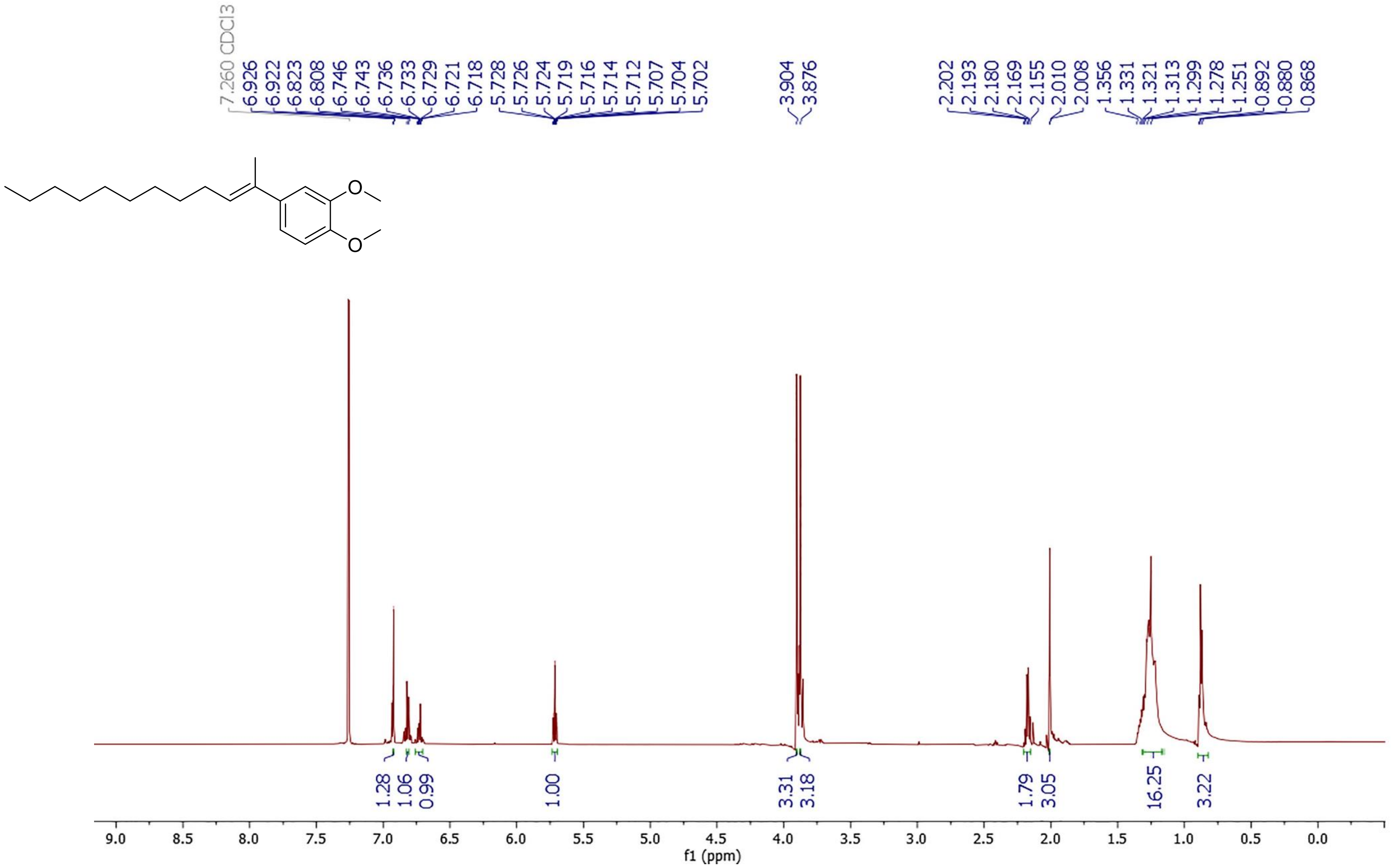

^{13}C-NMR (E)-4-(dodec-2-en-2-yl)-1,2-dimethoxybenzene (2m)

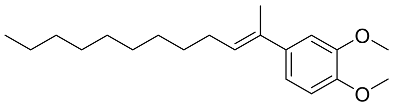

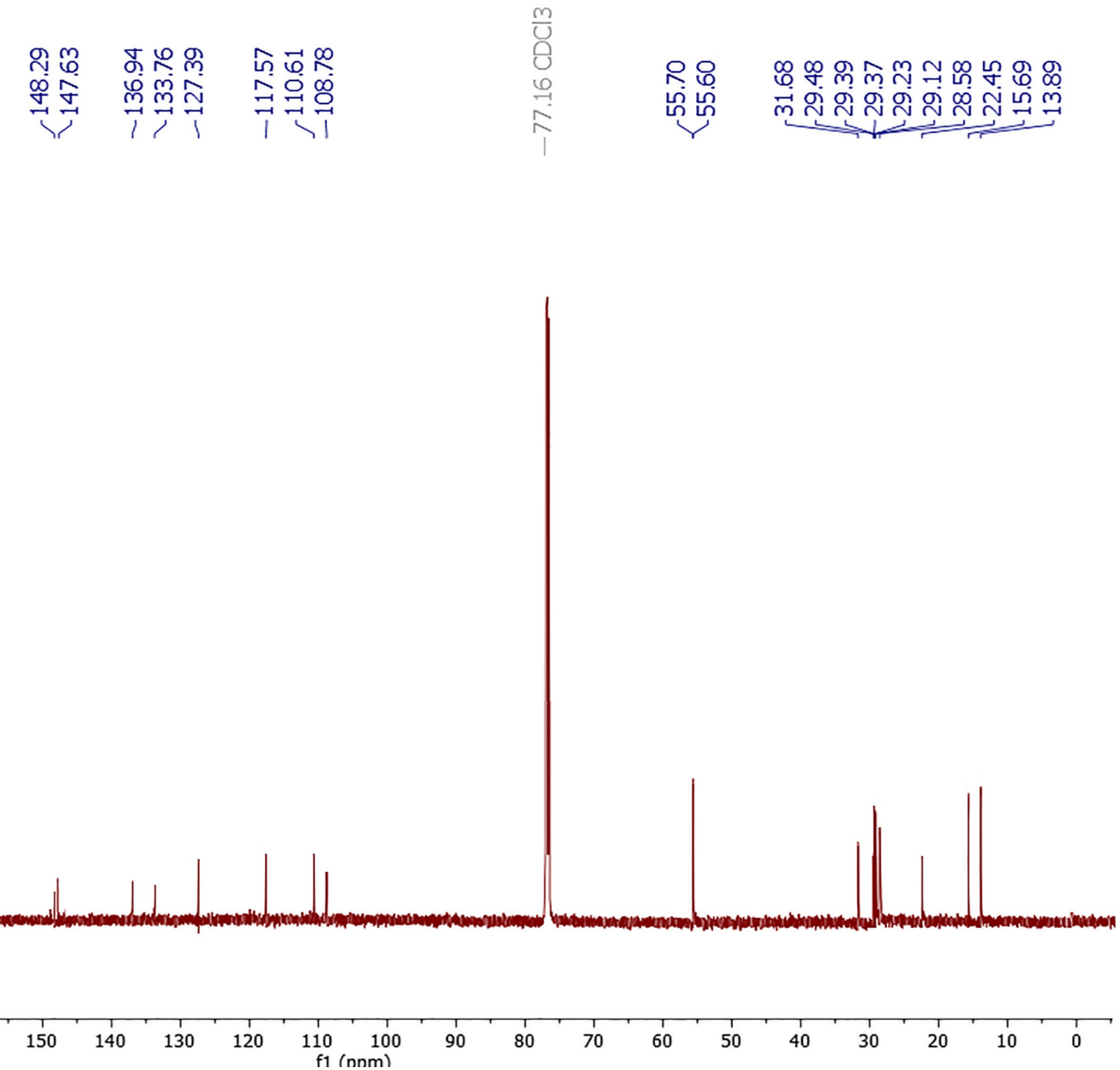

^{1}H-NMR (E)-1,2-dimethoxy-4-(4-methylhex-2-en-2-yl)benzene (2n)

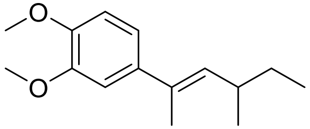

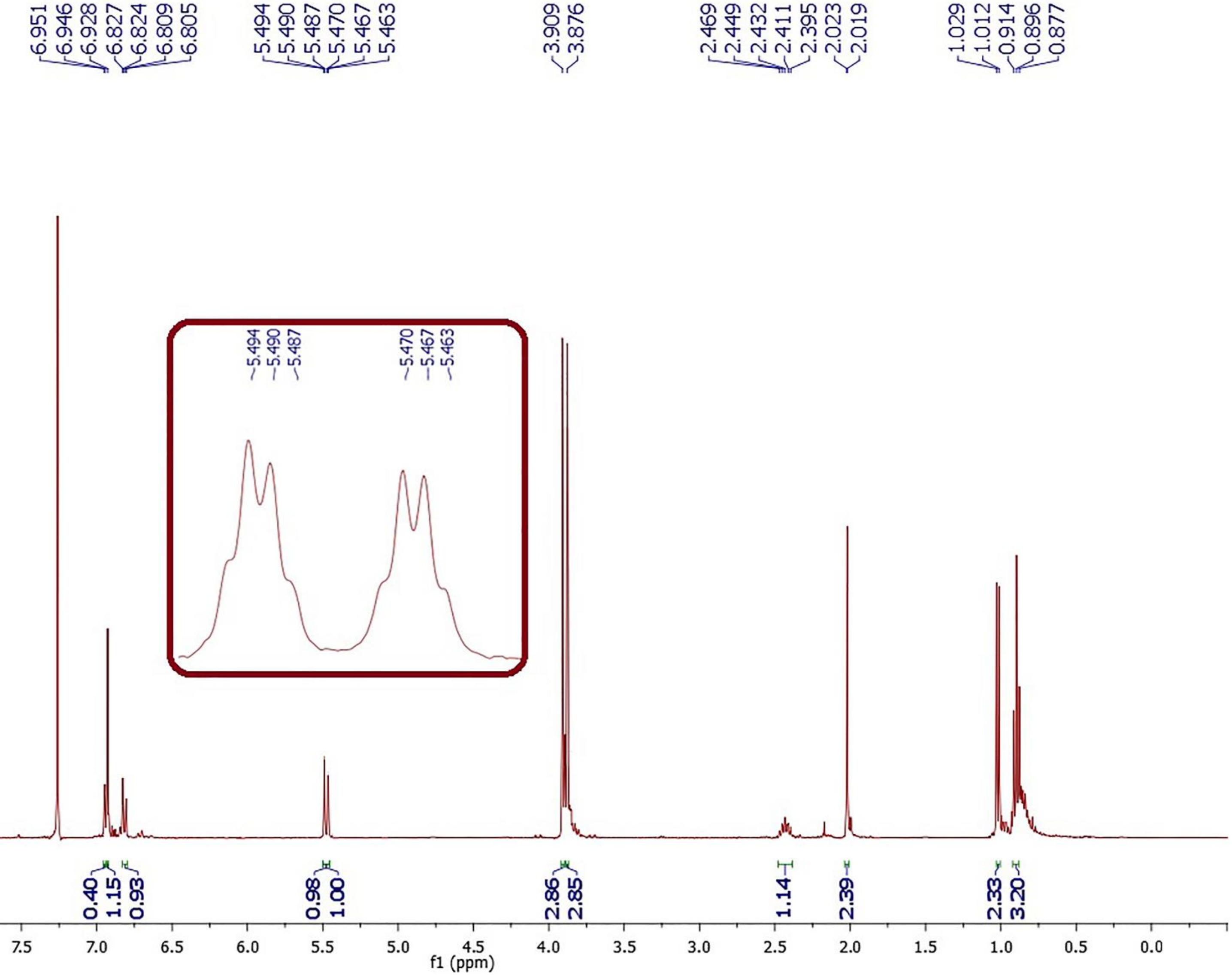

^{13}C-NMR (E)-1,2-dimethoxy-4-(4-methylhex-2-en-2-yl)benzene (2n)

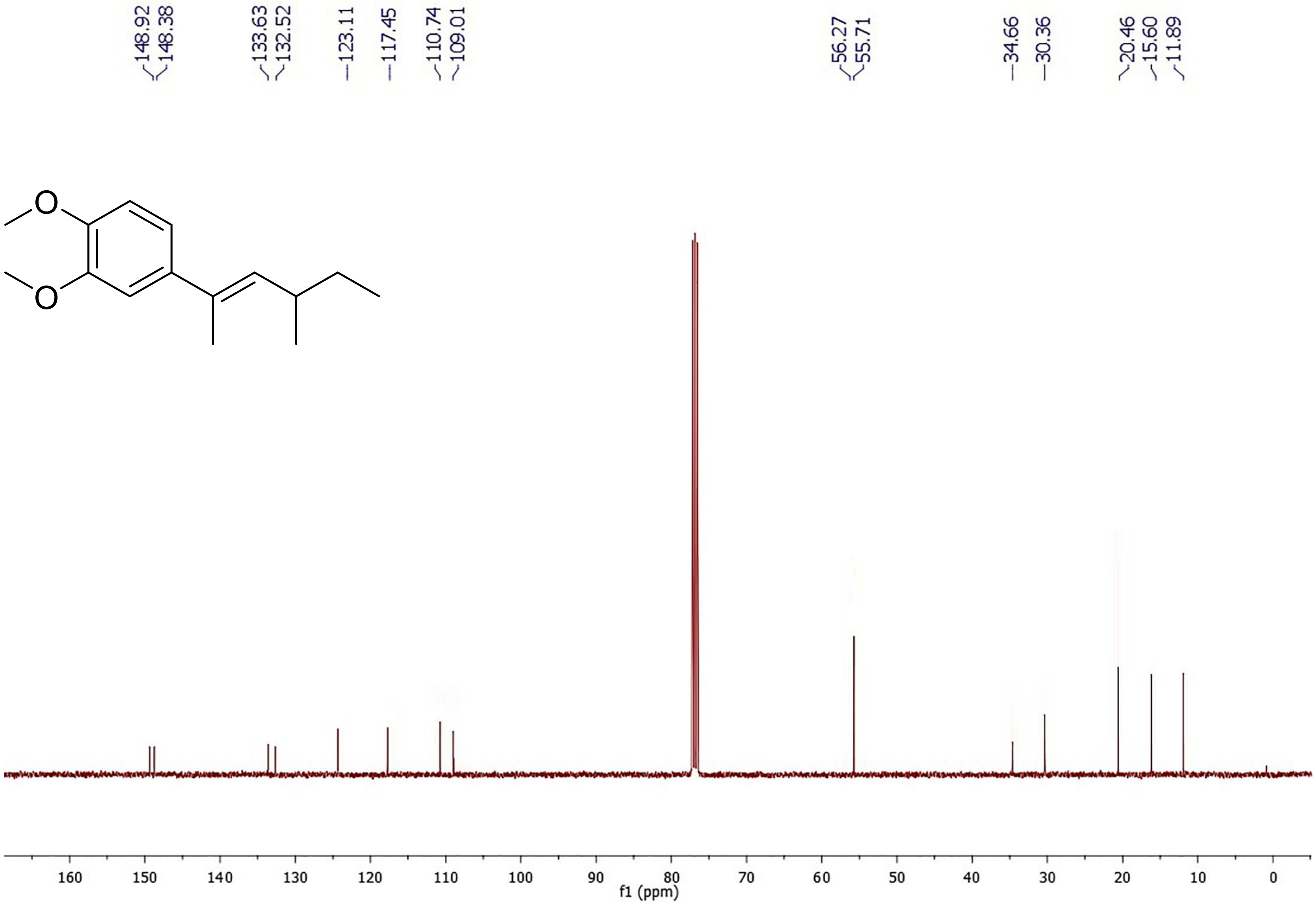

^{1}H-NMR (E)-1-(hept-2-en-2-yl)-3,5-dimethoxybenzene (2o)

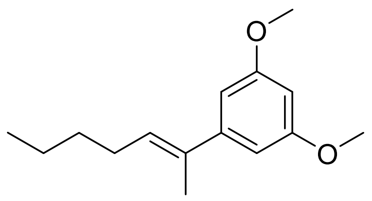

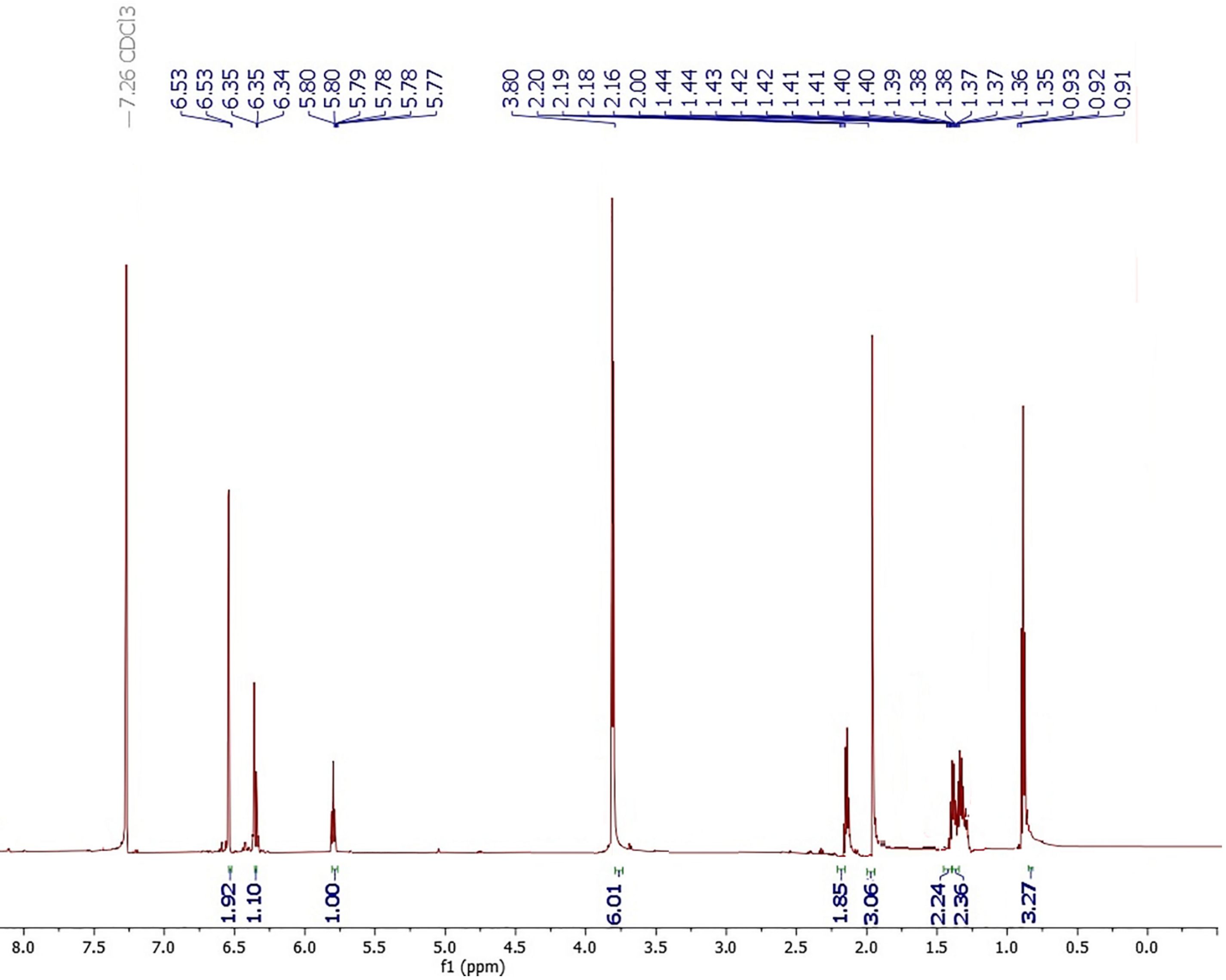

^{13}C-NMR (E)-1-(hept-2-en-2-yl)-3,5-dimethoxybenzene (2o)

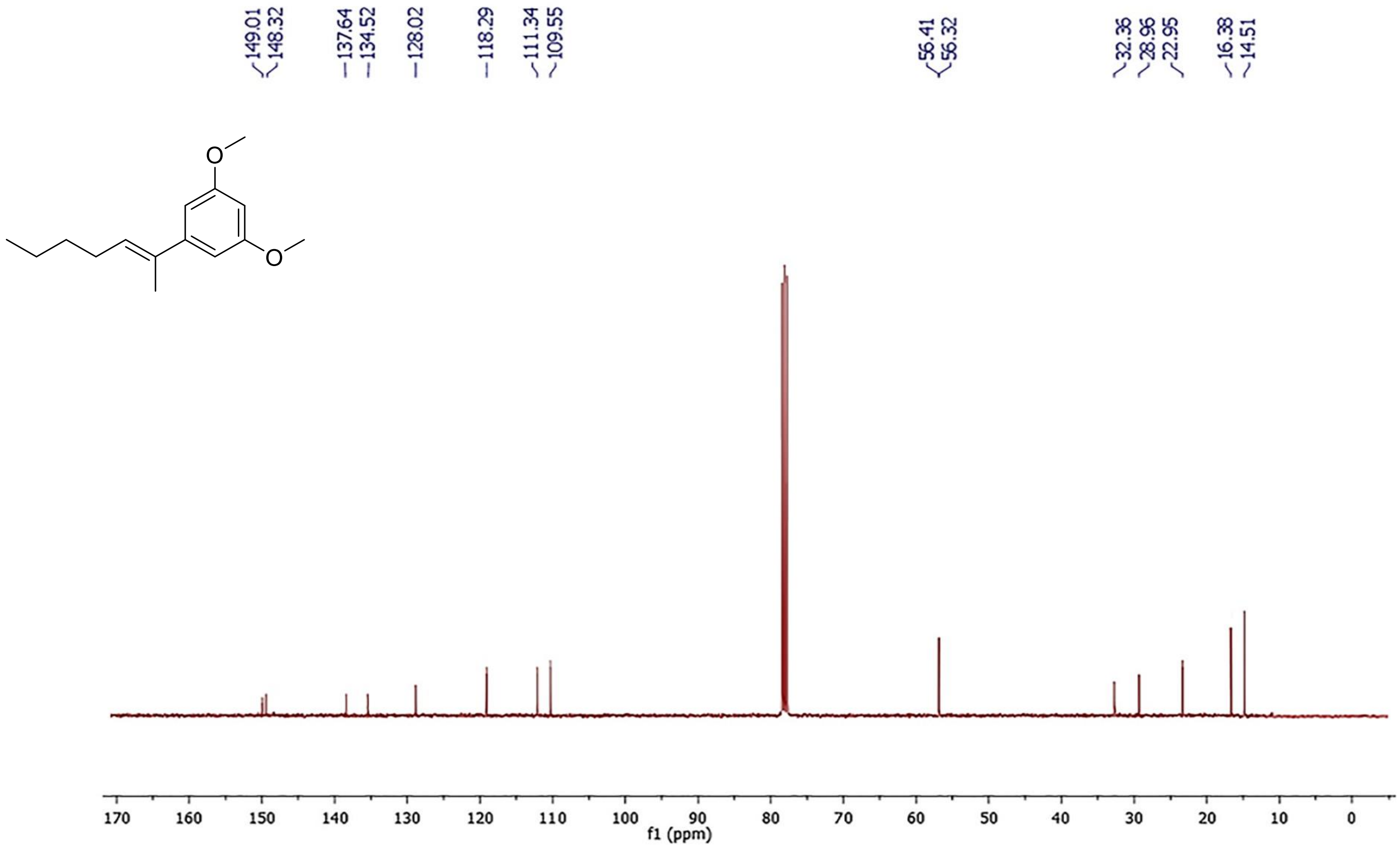

^{1}H-NMR (E)-1-(hex-2-en-2-yl)-3-phenoxybenzene (2p)

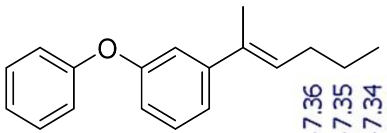

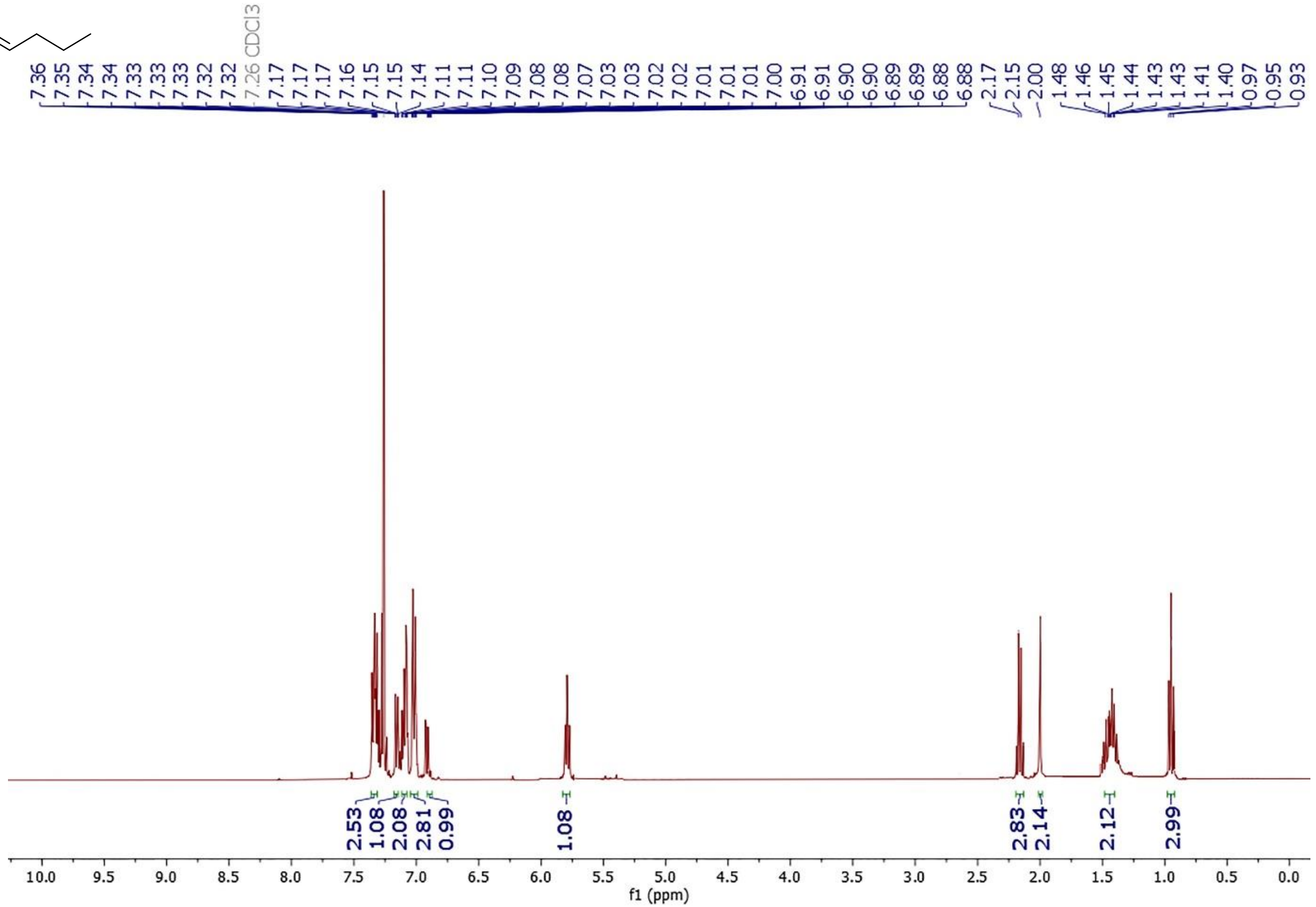

^{13}C-NMR (E)-1-(hex-2-en-2-yl)-3-phenoxybenzene (2p)

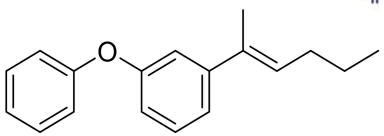

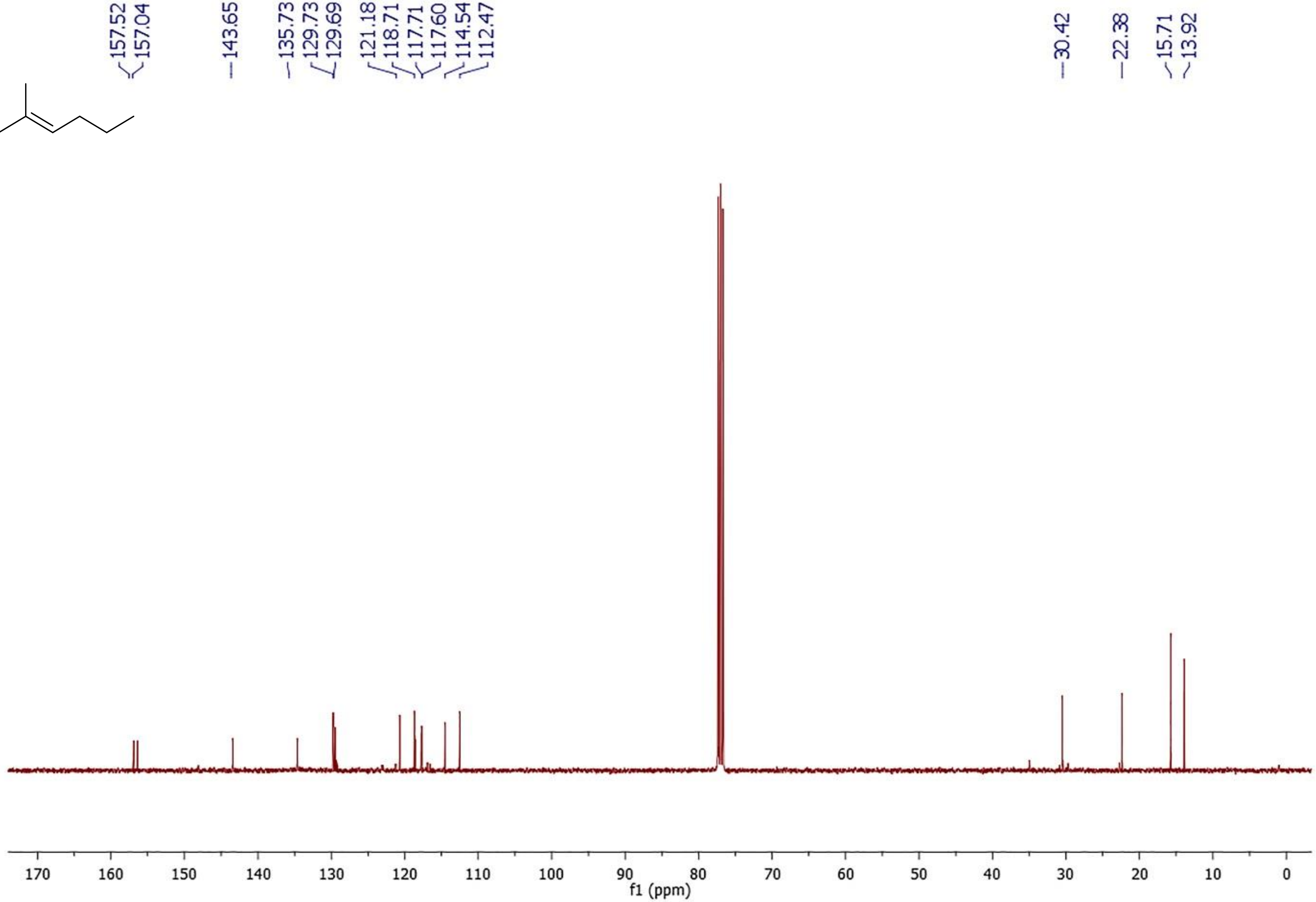

^{1}H-NMR 1-(hex-1-en-2-yl)-2,3,5-trimethoxybenzene (3q) *(major isomer)* & (E)-1-(hex-2-en-2-yl)-2,3,5-trimethoxybenzene (2q)

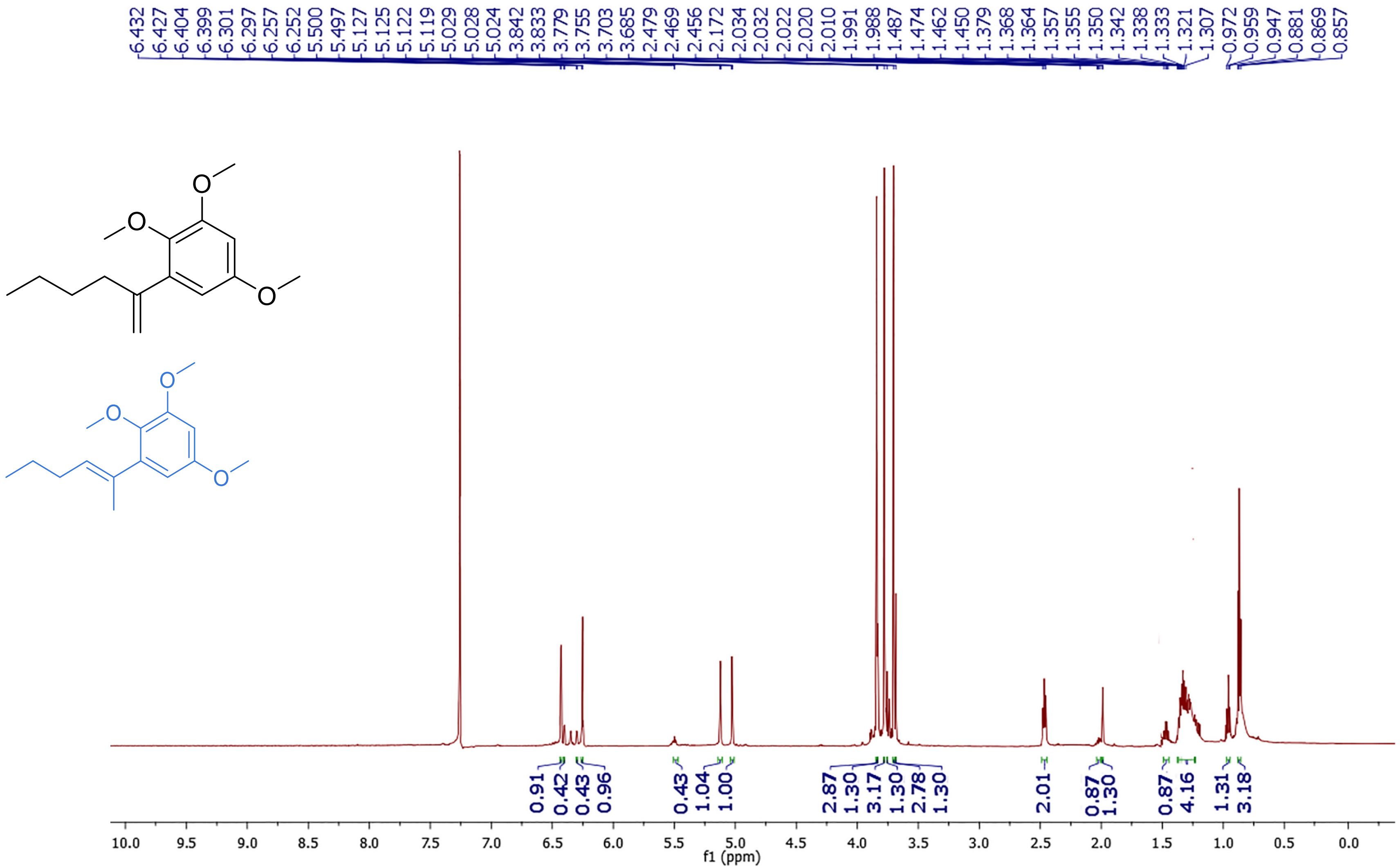

^{13}C-NMR 1-(hex-1-en-2-yl)-2,3,5-trimethoxybenzene (3q) ***(major isomer) &*** **(E)-1-(hex-2-en-2-yl)-2,3,5-trimethoxybenzene (2q)**

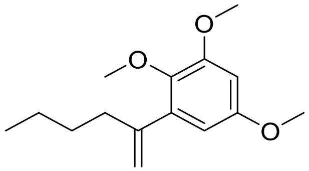

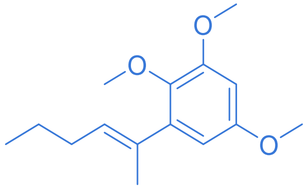

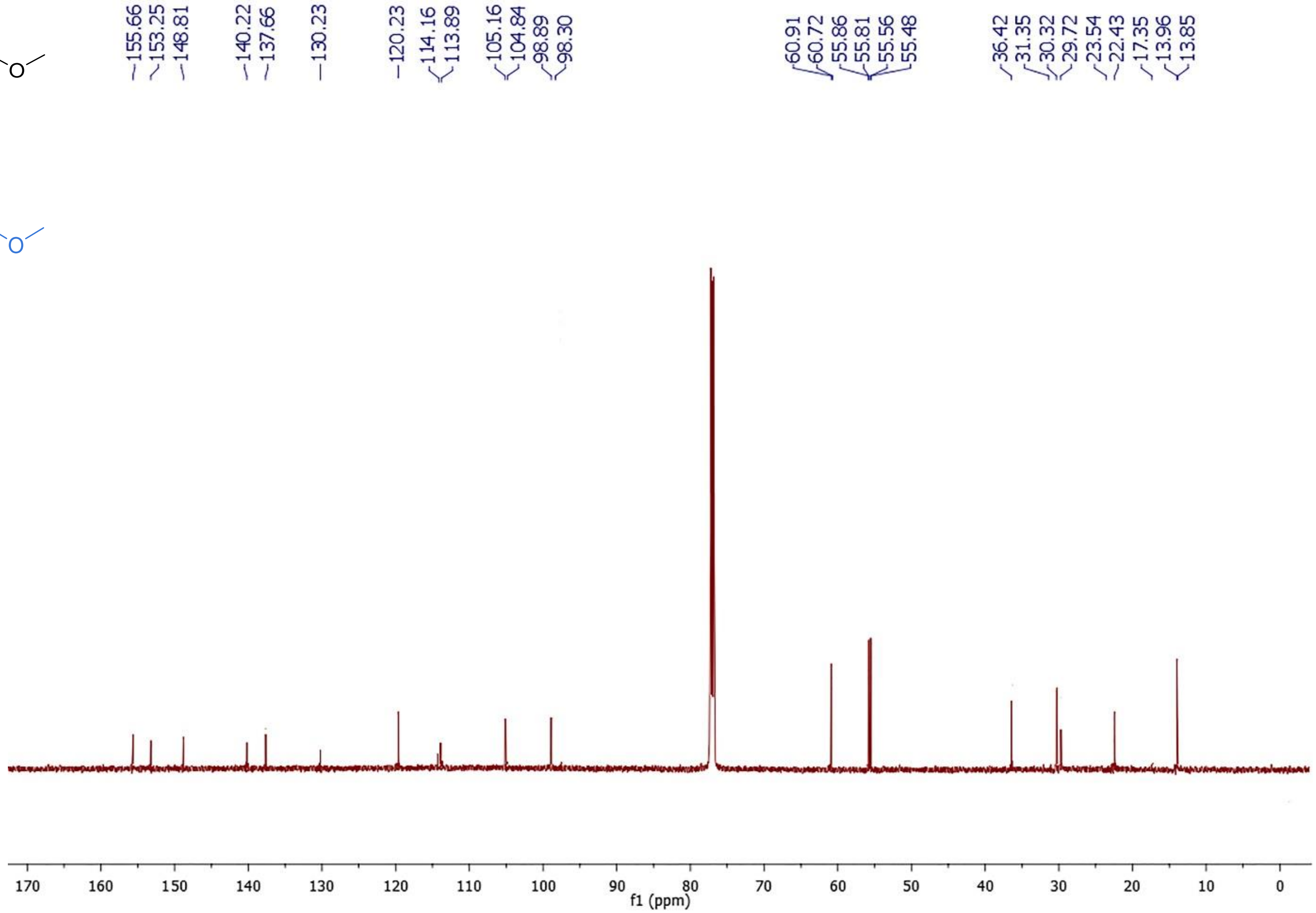

^{1}H-NMR 1,2,5-trimethoxy-3-(oct-1-en-2-yl)benzene (3r) *(major isomer)*& (E)-1,2,5-trimethoxy-3-(oct-2-en-2-yl)benzene (2r)

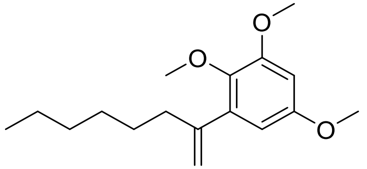

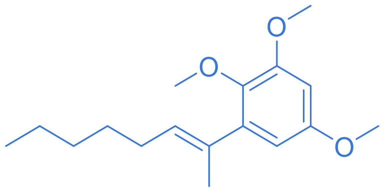

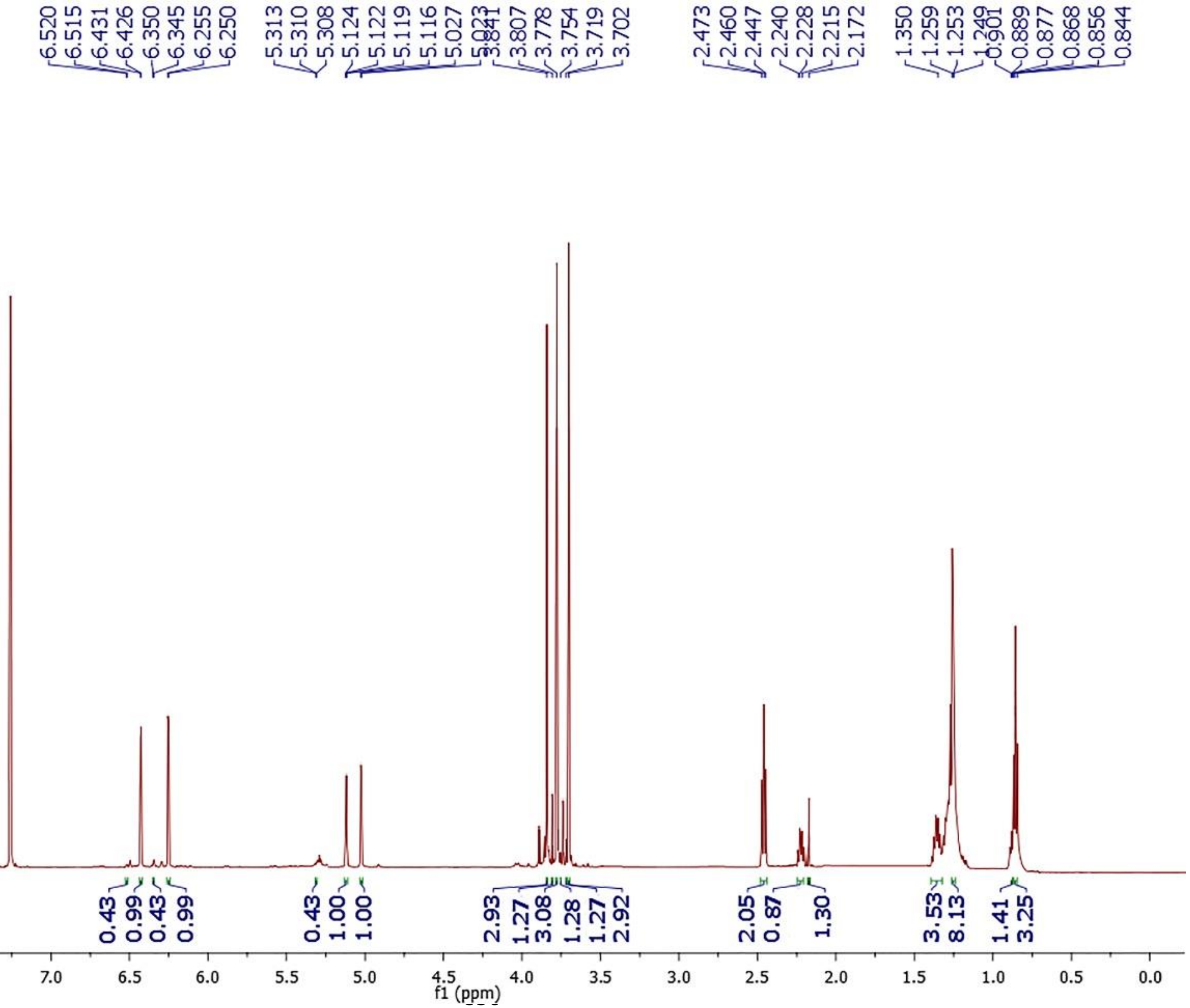

^{13}C-NMR 1,2,5-trimethoxy-3-(oct-1-en-2-yl)benzene (3r) *(major isomer)&* (E)-1,2,5-trimethoxy-3-(oct-2-en-2-yl)benzene (2r)

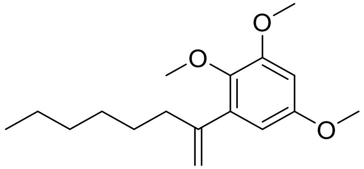

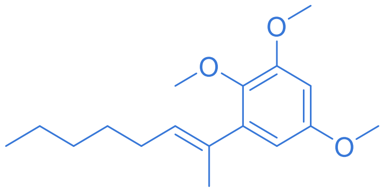

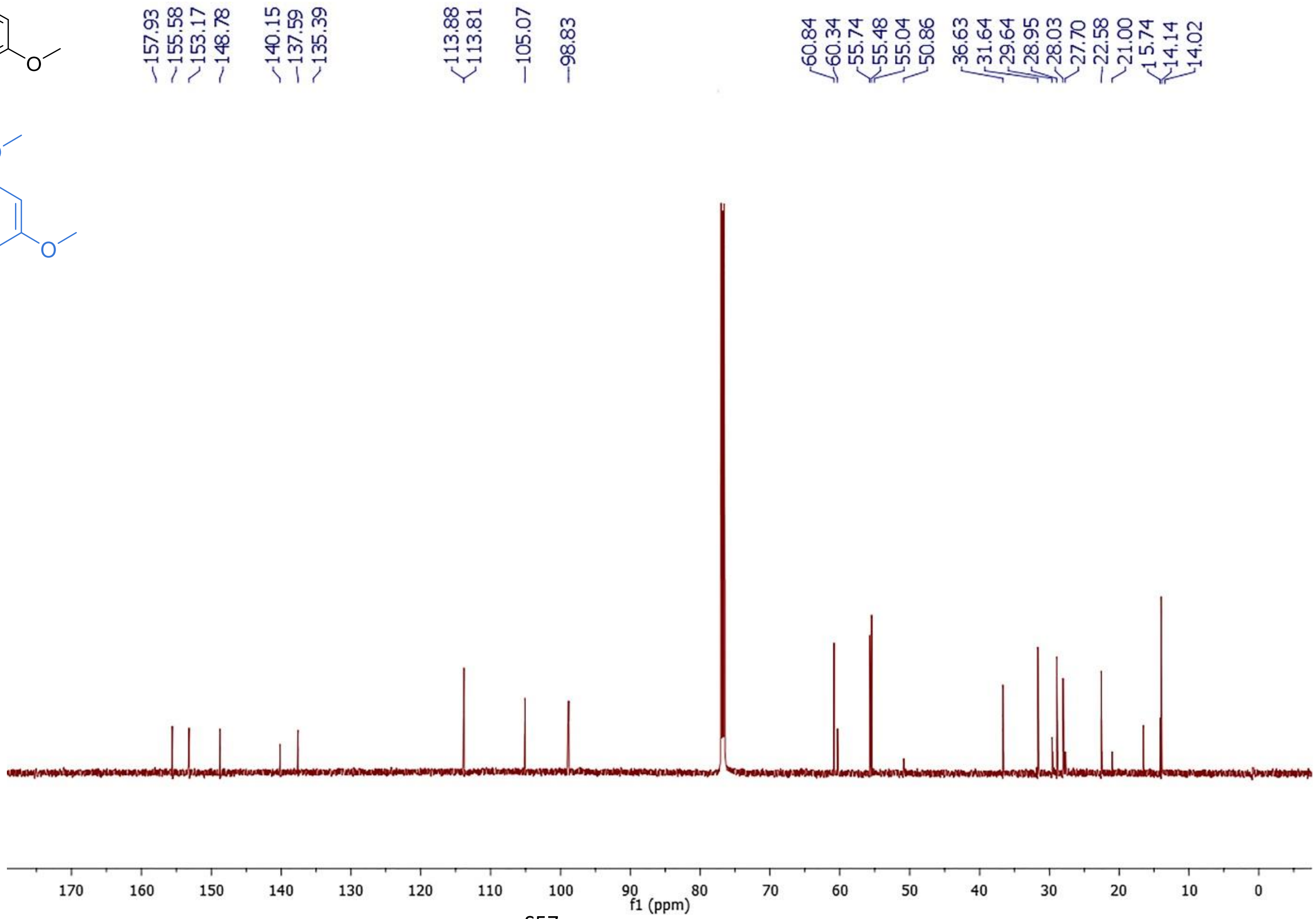

^{1}H-NMR (E)-5-(oct-2-en-2-yl)benzo[d][1,3]dioxole (2s) ***(major isomer) &*** **4-(oct-1-en-2-yl)benzo[d][1,3]dioxole (3s)**

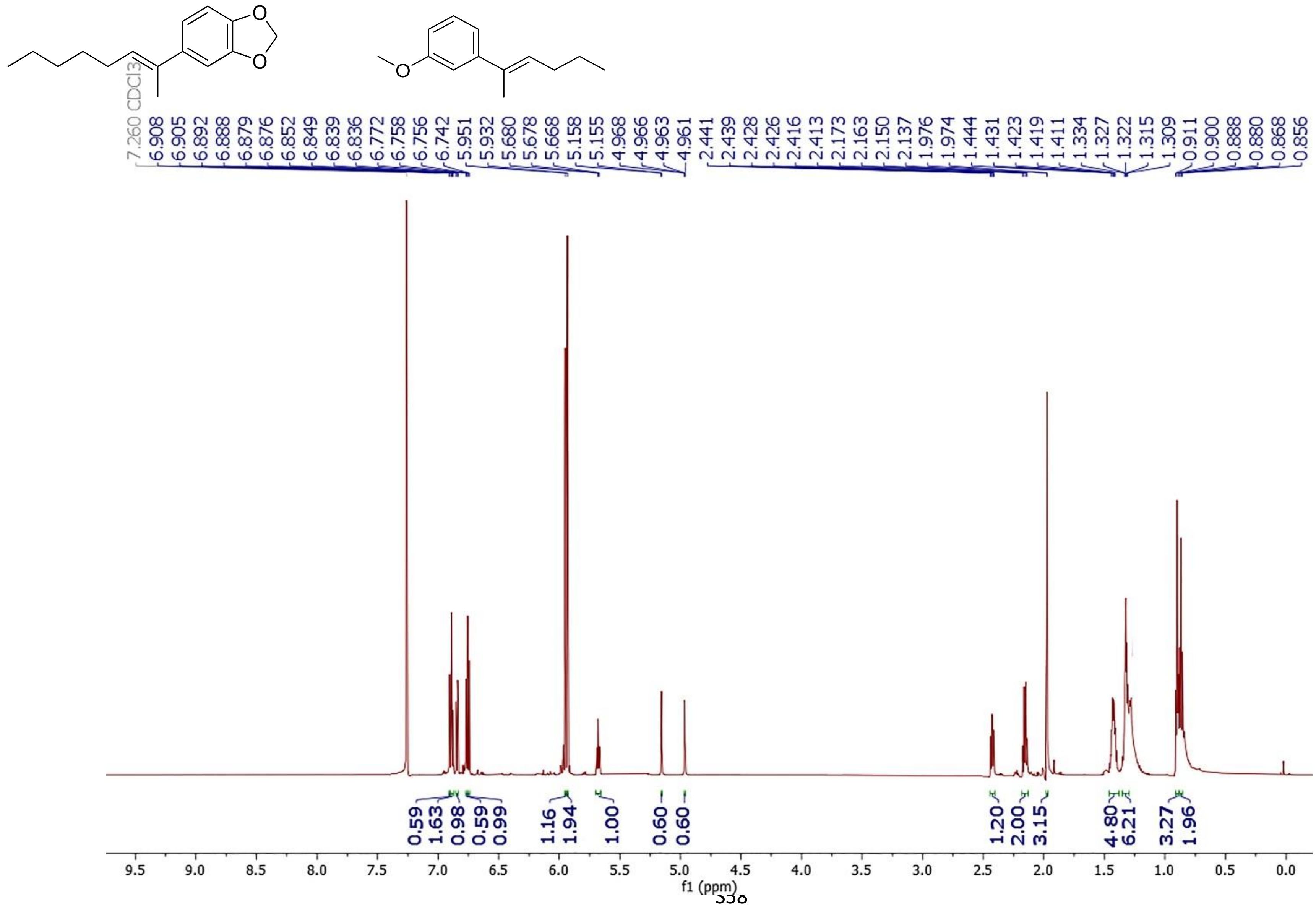

^{13}C-NMR (E)-5-(oct-2-en-2-yl)benzo[d][1,3]dioxole (2s) ***(major isomer) &*** **4-(oct-1-en-2-yl)benzo[d][1,3]dioxole (3s)**

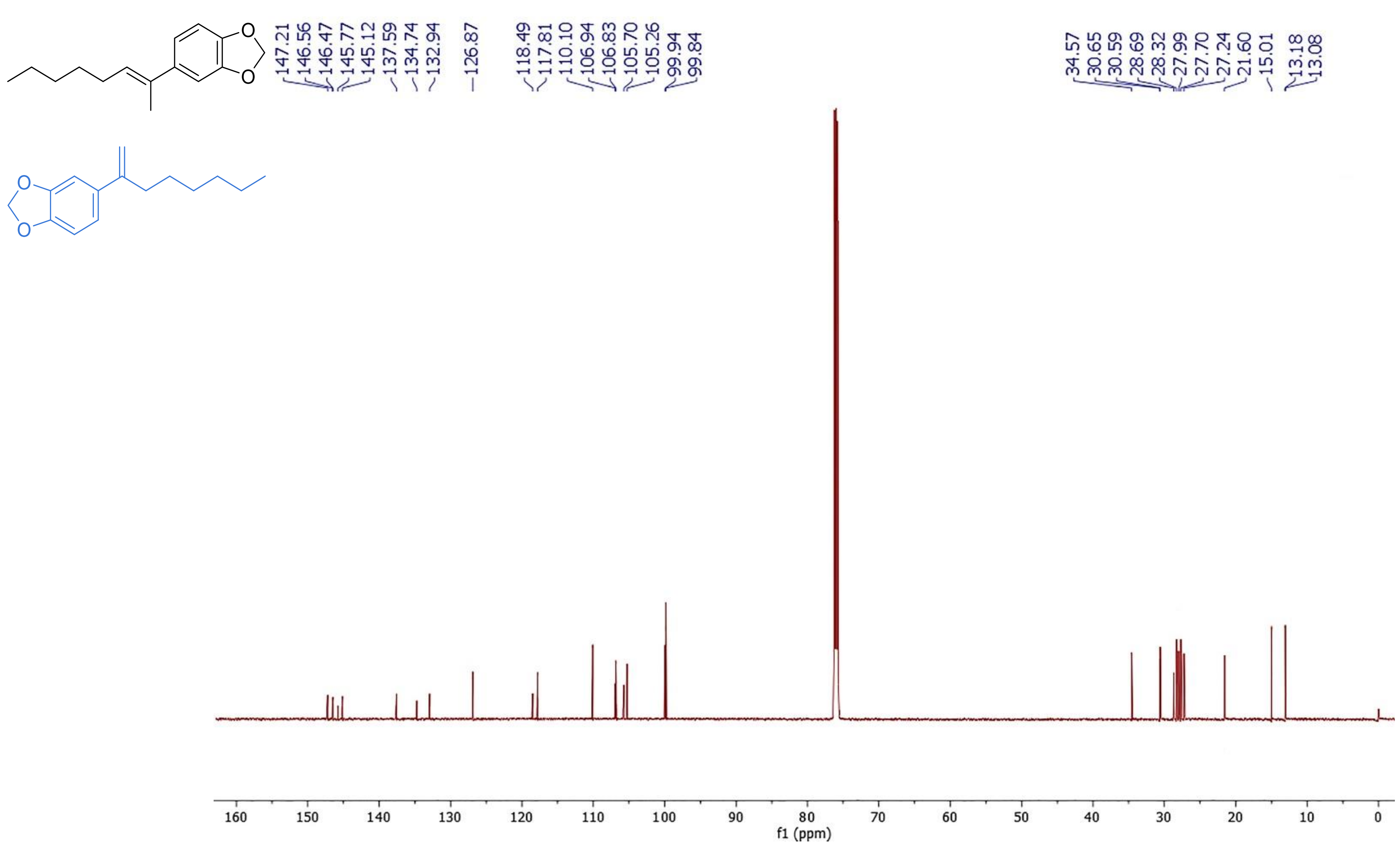

^{1}H-NMR 3-methoxy-1-(oct-1-en-2-yl)naphthalene (3t) ***(major isomer)*** **& (E)-3-methoxy-1-(oct-2-en-2-yl)naphthalene (2t)**

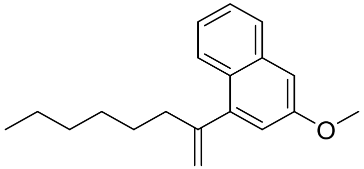

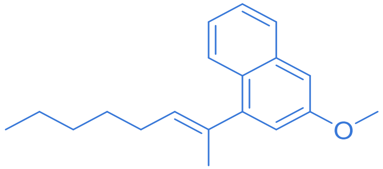

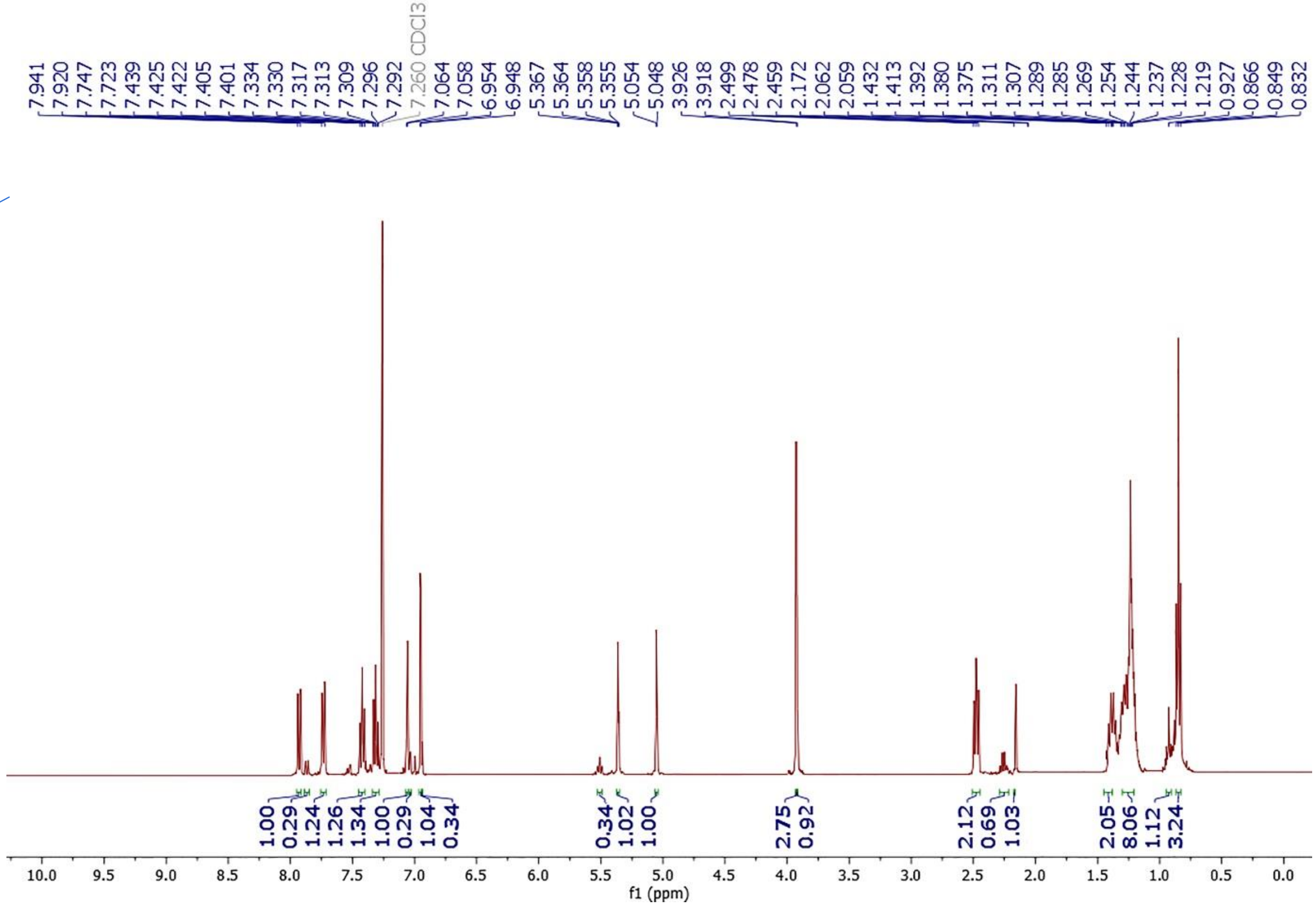

^{13}C-NMR 3-methoxy-1-(oct-1-en-2-yl)naphthalene (3t) ***(major isomer) &*** **(E)-3-methoxy-1-(oct-2-en-2-yl)naphthalene (2t)**

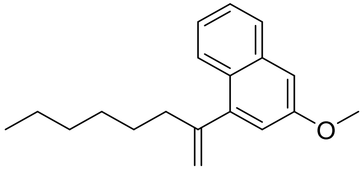

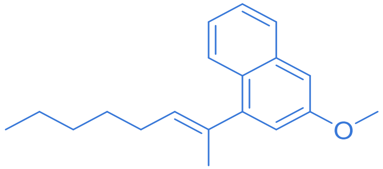

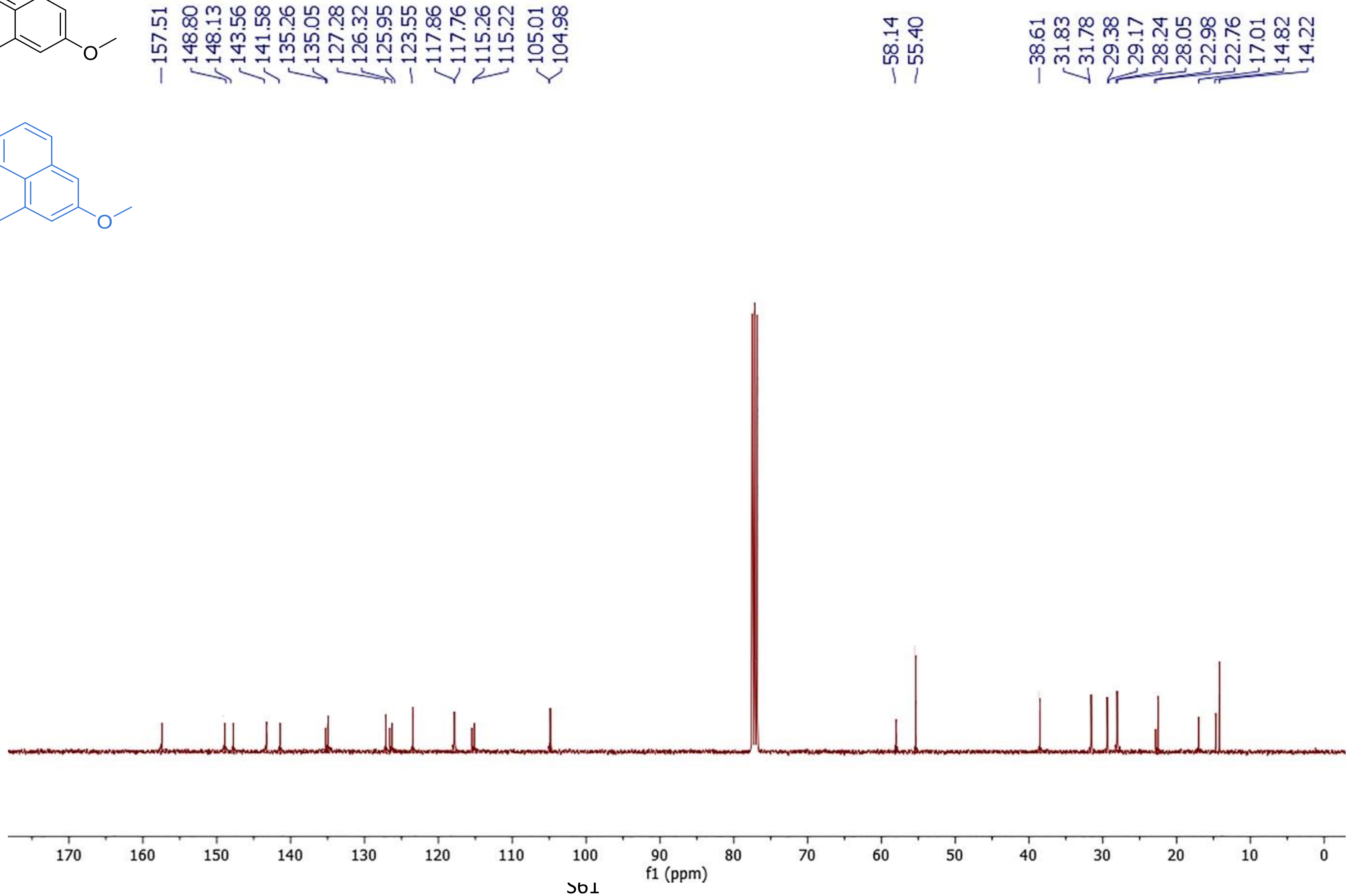

^{1}H-NMR 3-methoxy-1-(undec-1-en-2-yl)naphthalene (3u) ***(major isomer) &*** **(E)-1-(dodec-2-en-2-yl)-3-methoxynaphthalene (2u)**

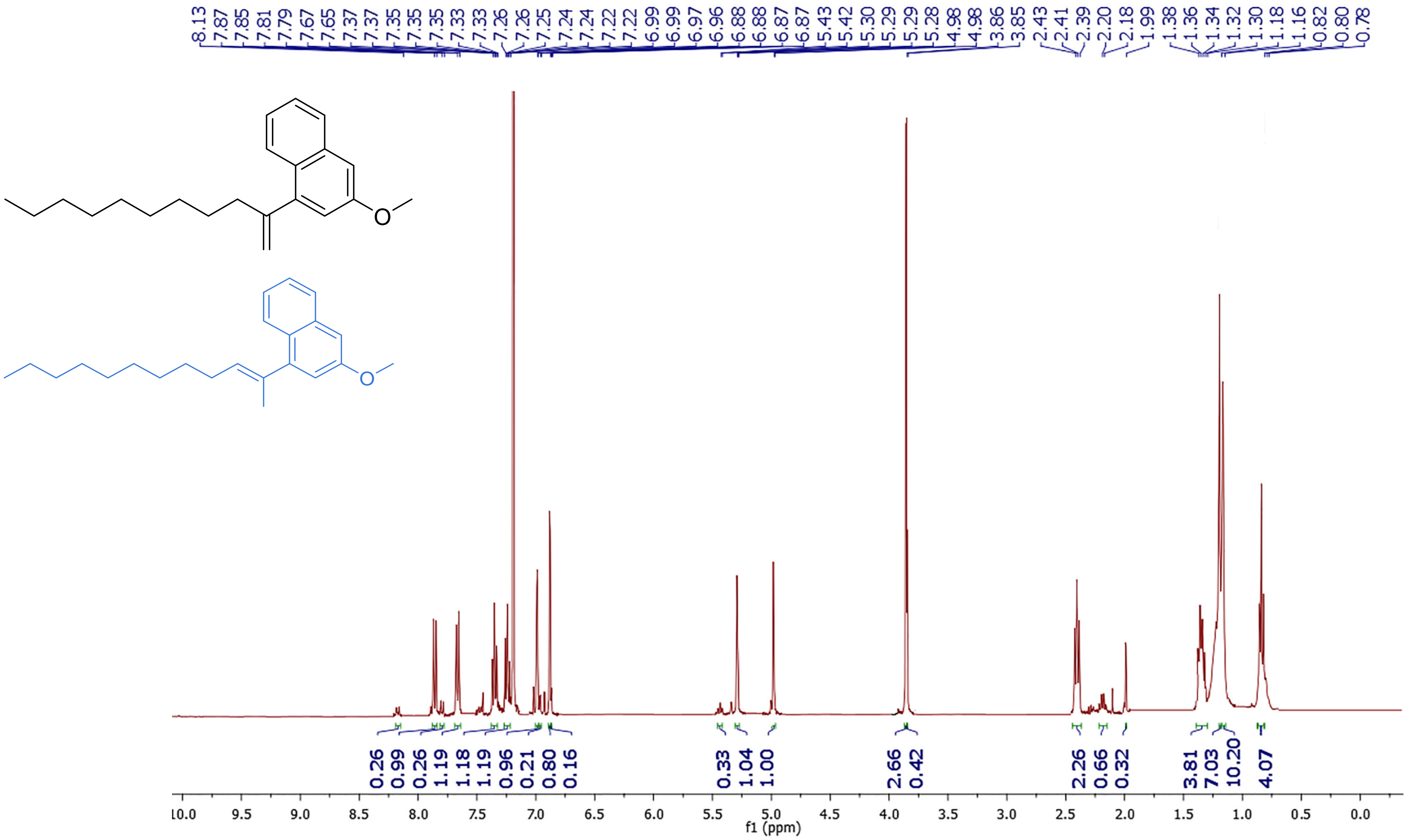

^{13}C-NMR 3-methoxy-1-(undec-1-en-2-yl)naphthalene (3u) *(major isomer)* & (E)-1-(dodec-2-en-2-yl)-3-methoxynaphthalene (2u)

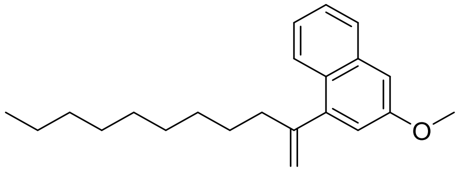

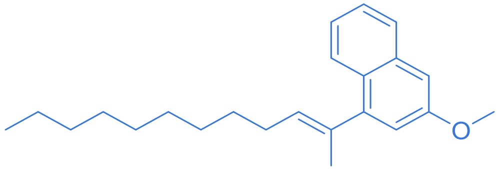

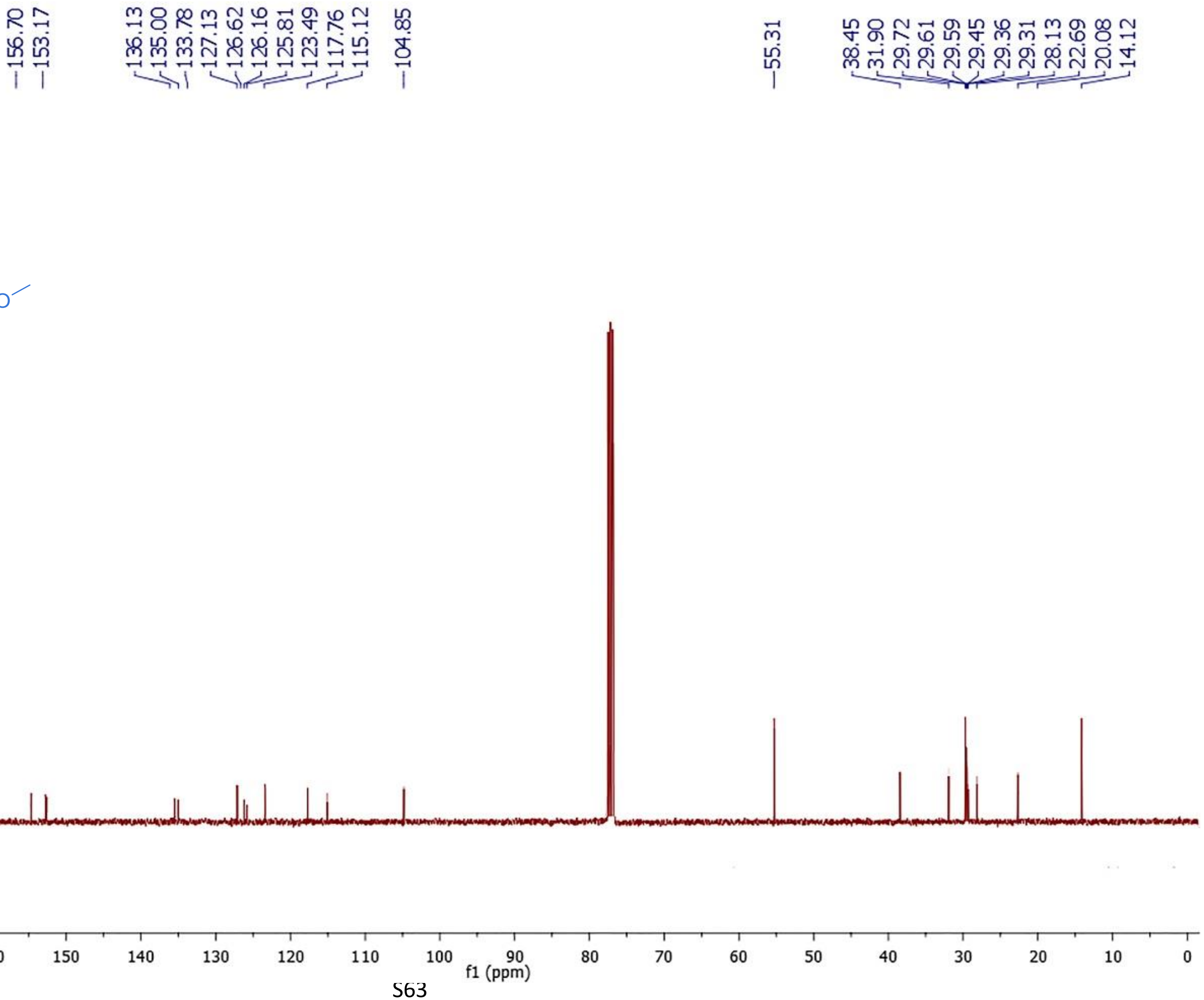

^{1}H-NMR 1-methoxy-3-methyl-5-(4-phenylbut-1-en-2-yl)benzene (3v) ***(major isomer) &*** **(E)-1-methoxy-3-methyl-5-(4-phenylbut-2-en-2-yl)benzene(2v)**

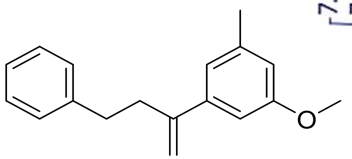

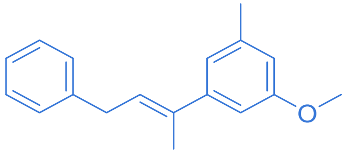

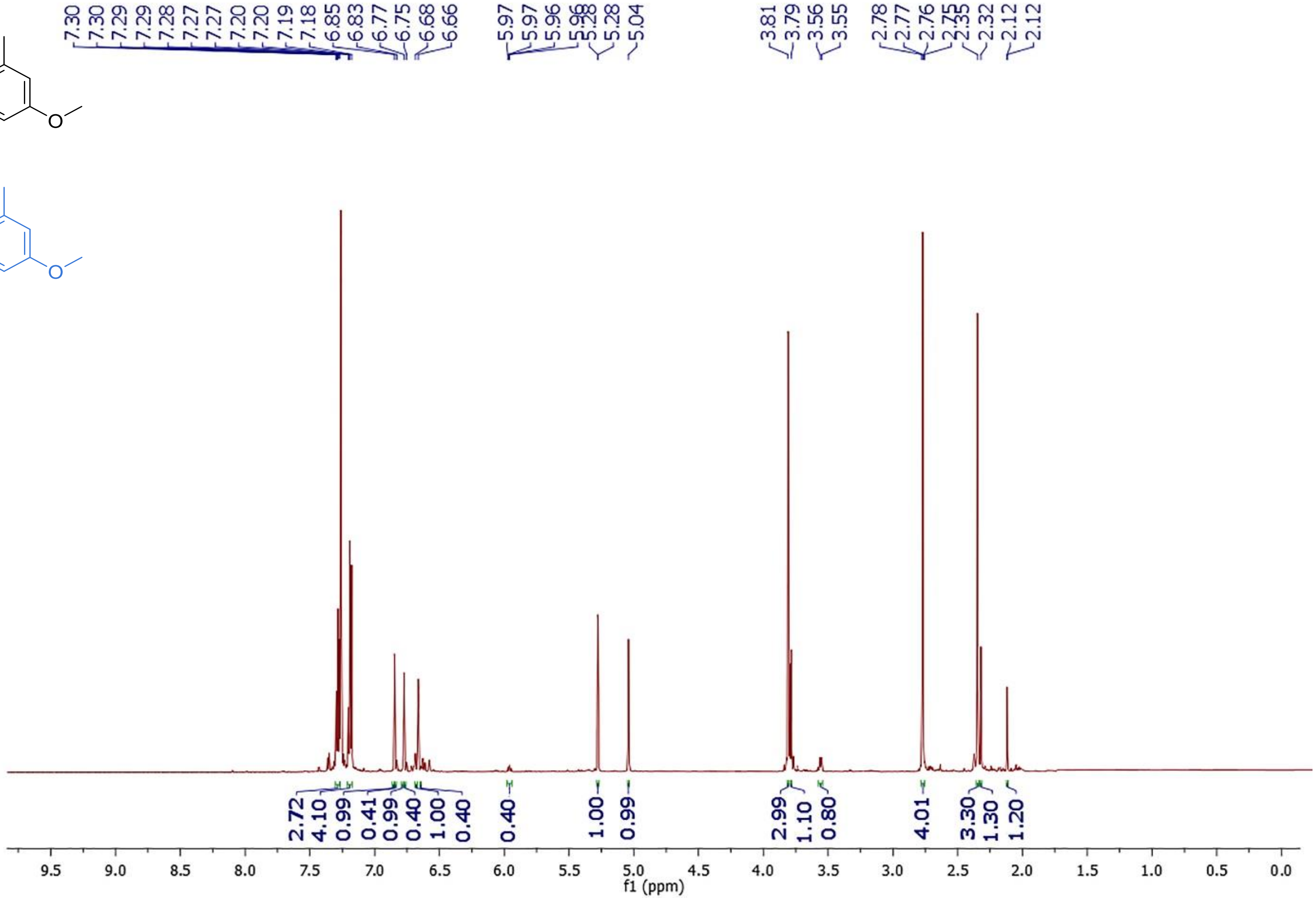

^{13}C-NMR 1-methoxy-3-methyl-5-(4-phenylbut-1-en-2-yl)benzene (3v) *(major isomer)* & (E)-1-methoxy-3-methyl-5-(4-phenylbut-2-en-2-yl)benzene(2v)

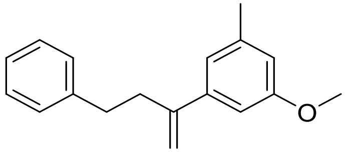

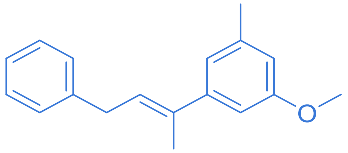

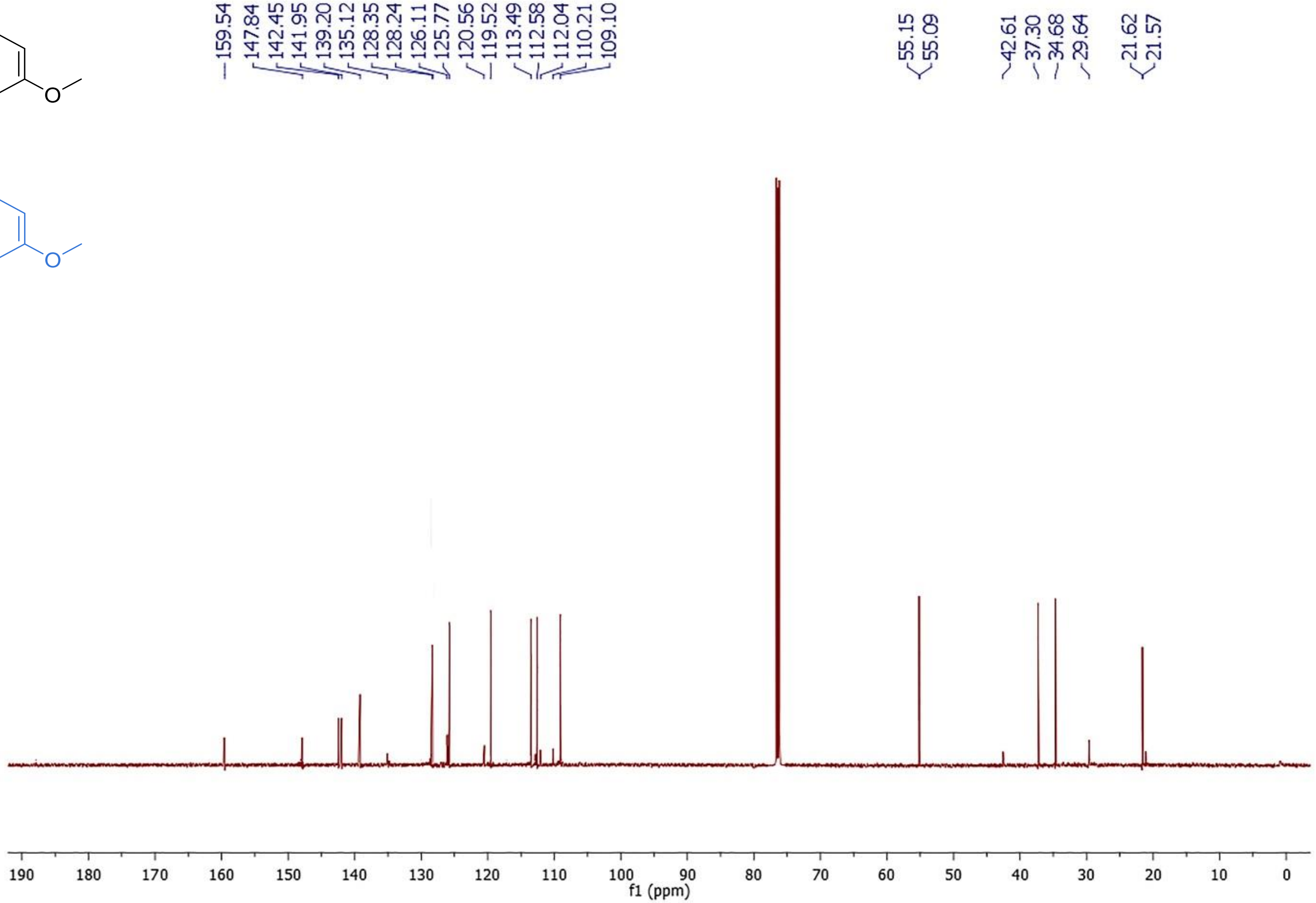

^{1}H-NMR 1-(3,7-dimethyloct-1-en-2-yl)-3-methoxy-5-methylbenzene (3w)

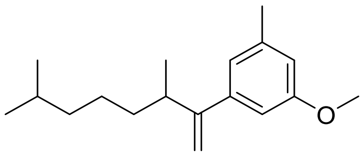

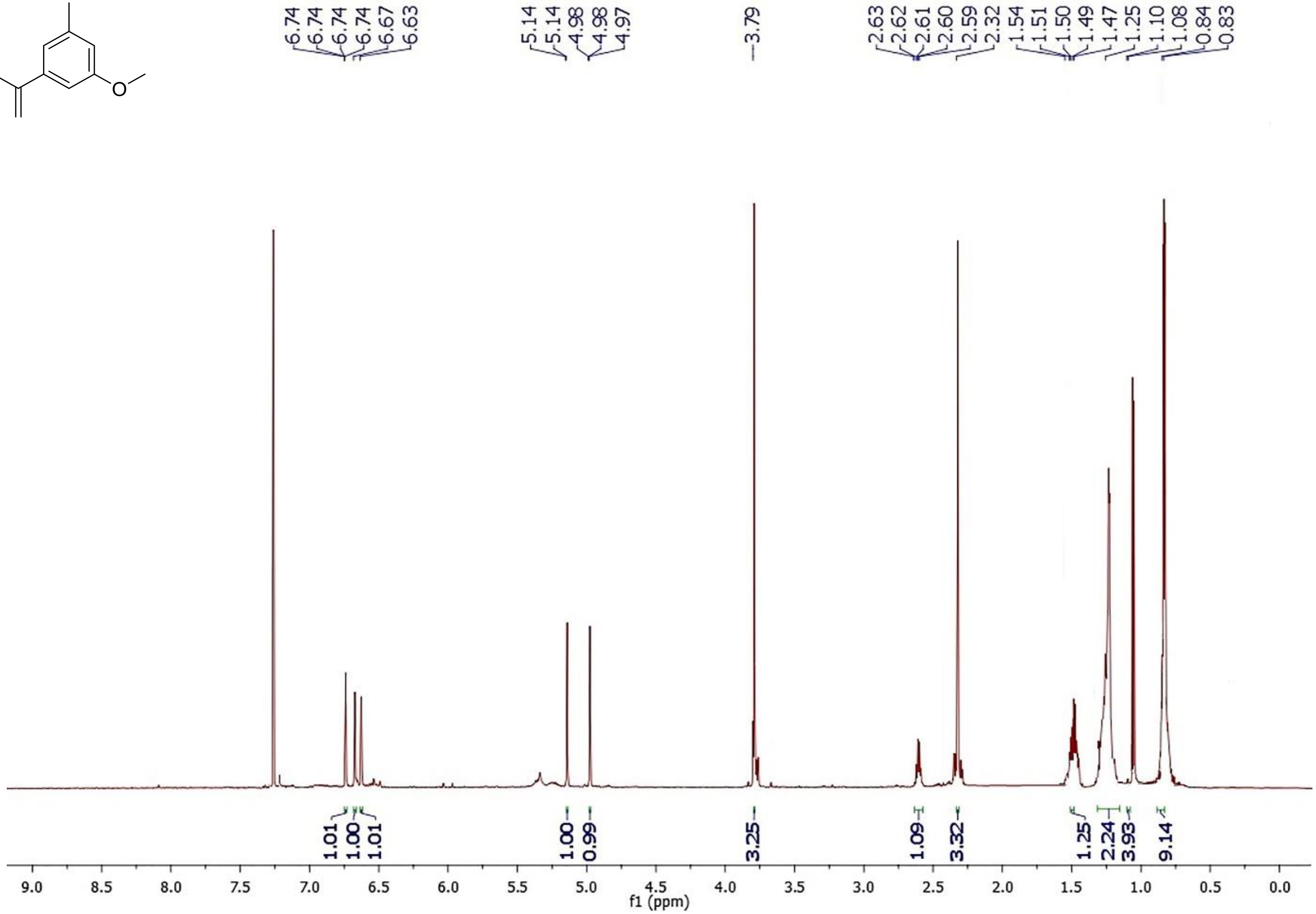

[13]C-APT 1-(3,7-dimethyloct-1-en-2-yl)-3-methoxy-5-methylbenzene (3w)

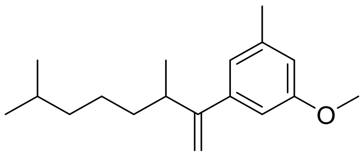

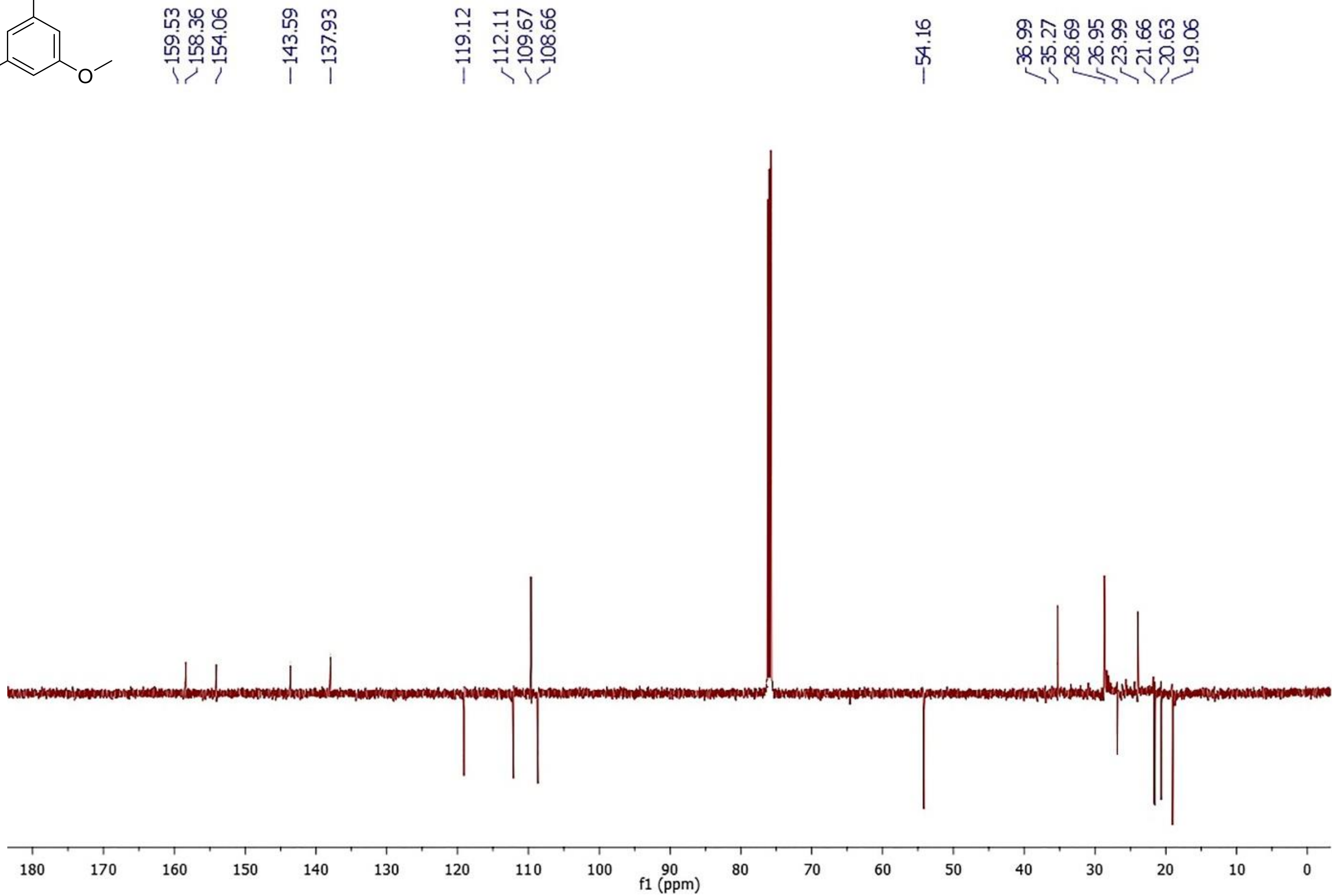

^{1}H-NMR 1-(1-cyclohexylvinyl)-3-methoxybenzene (3x)

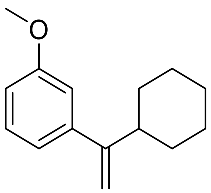

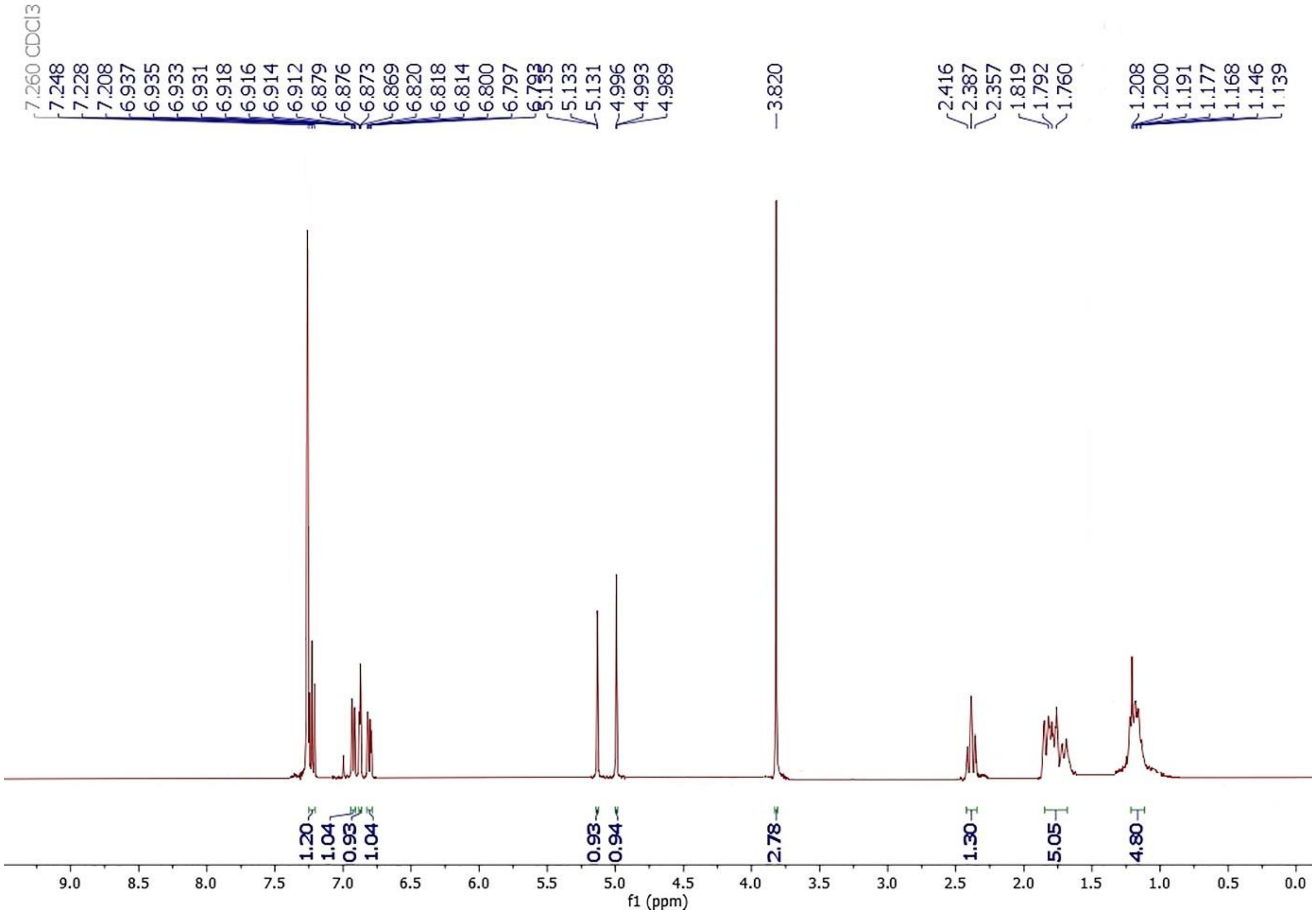

^{13}C-NMR 1-(1-cyclohexylvinyl)-3-methoxybenzene (3x)

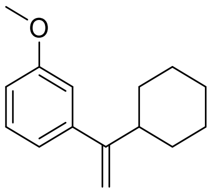

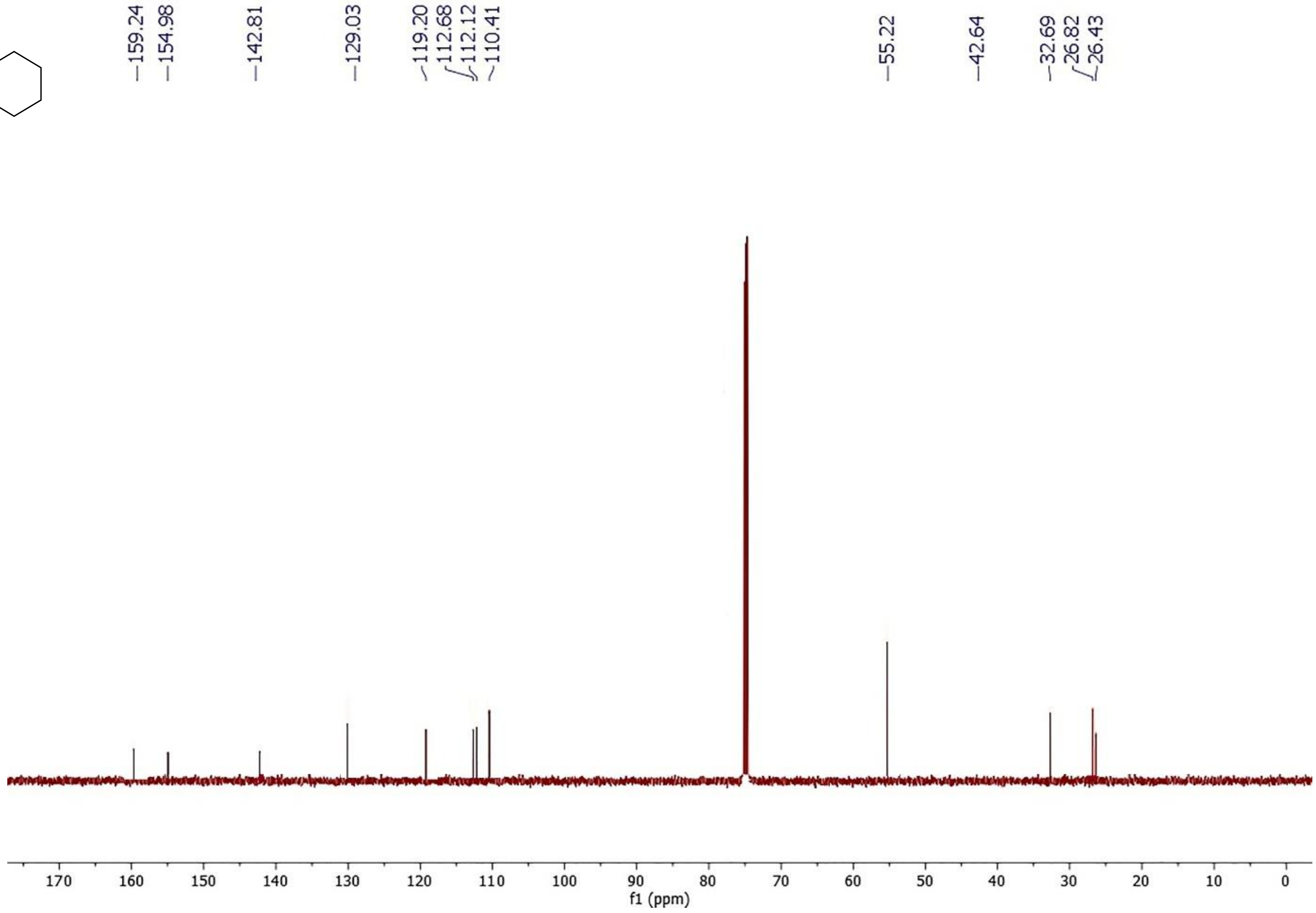

^{1}H-NMR 1-(1-cyclohexylvinyl)-2,3,5-trimethoxybenzene (3y)

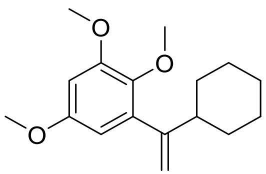

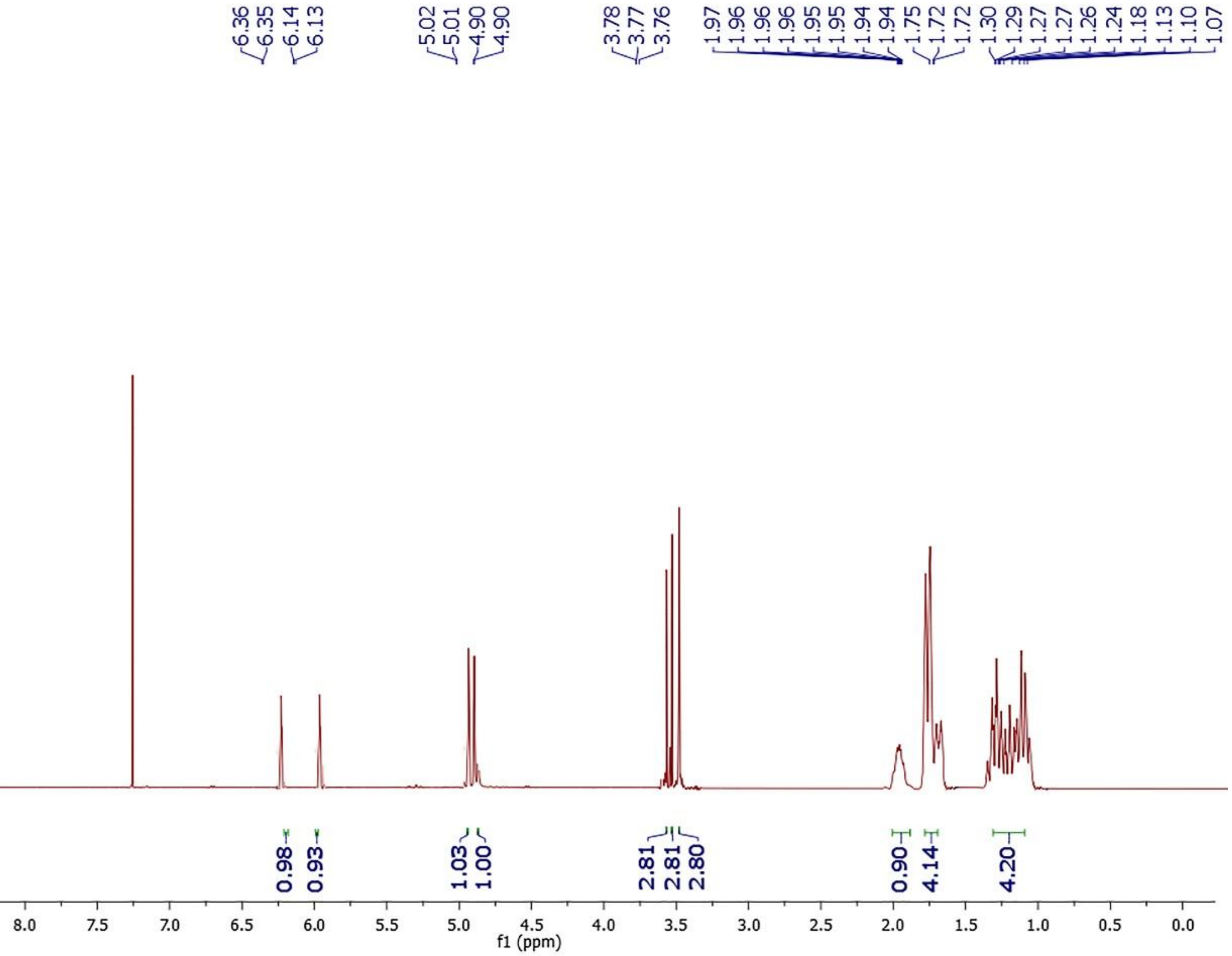

^{13}C-NMR 1-(1-cyclohexylvinyl)-2,3,5-trimethoxybenzene (3y)

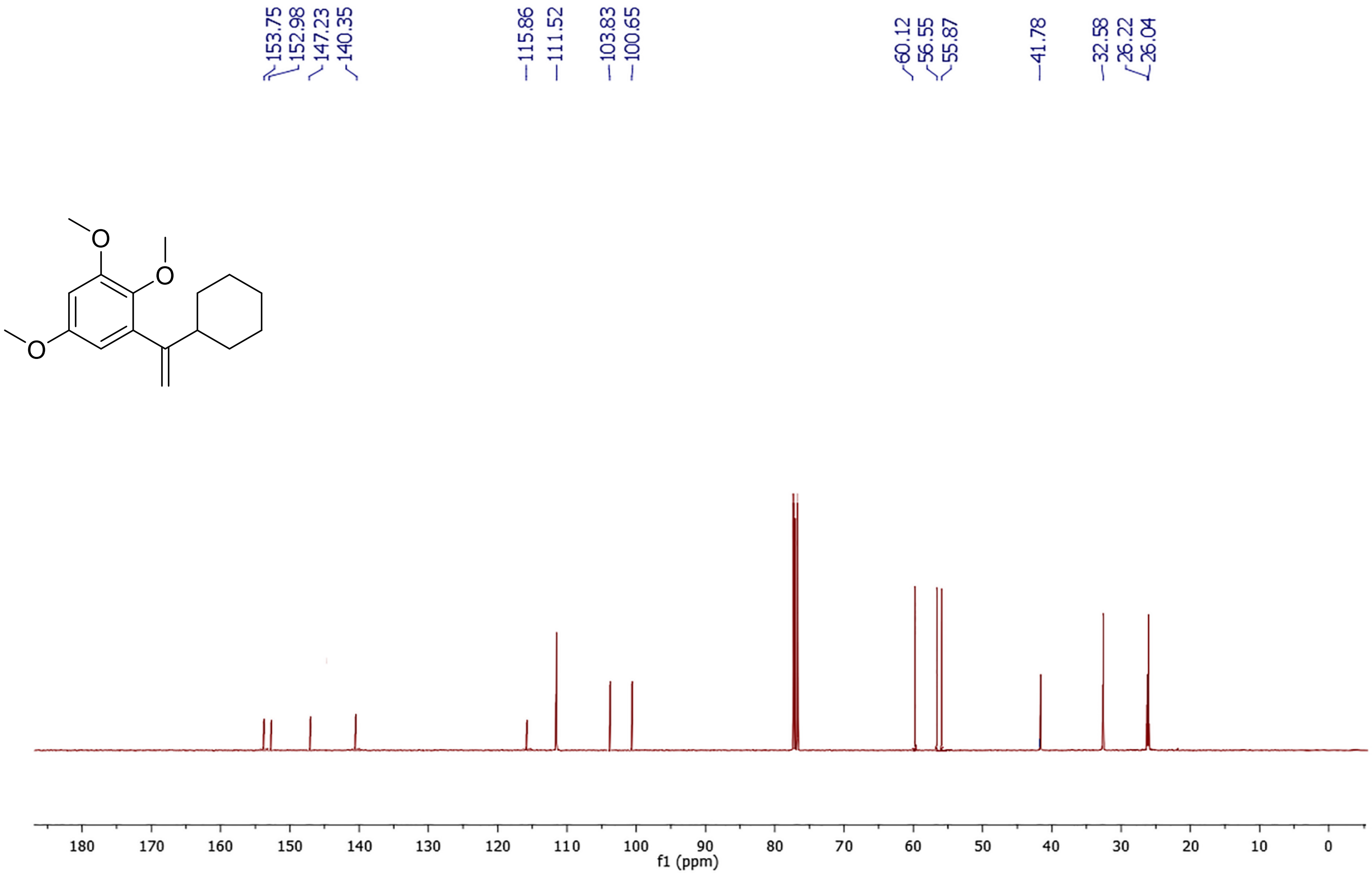

^{1}H-NMR (E)-1-fluoro-3-(oct-2-en-2-yl)benzene (2z)

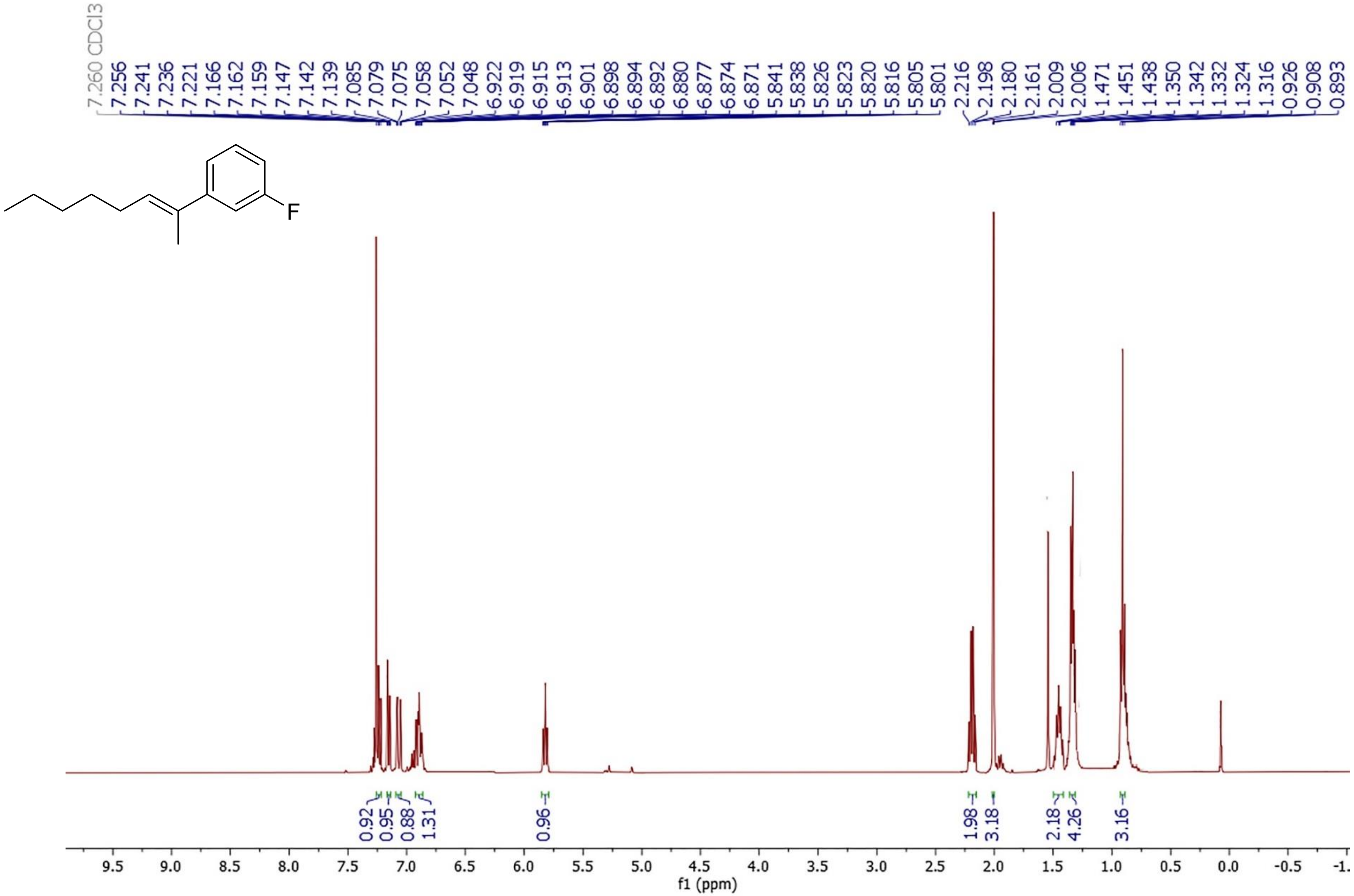

^{13}C-NMR (E)-1-fluoro-3-(oct-2-en-2-yl)benzene (2z)

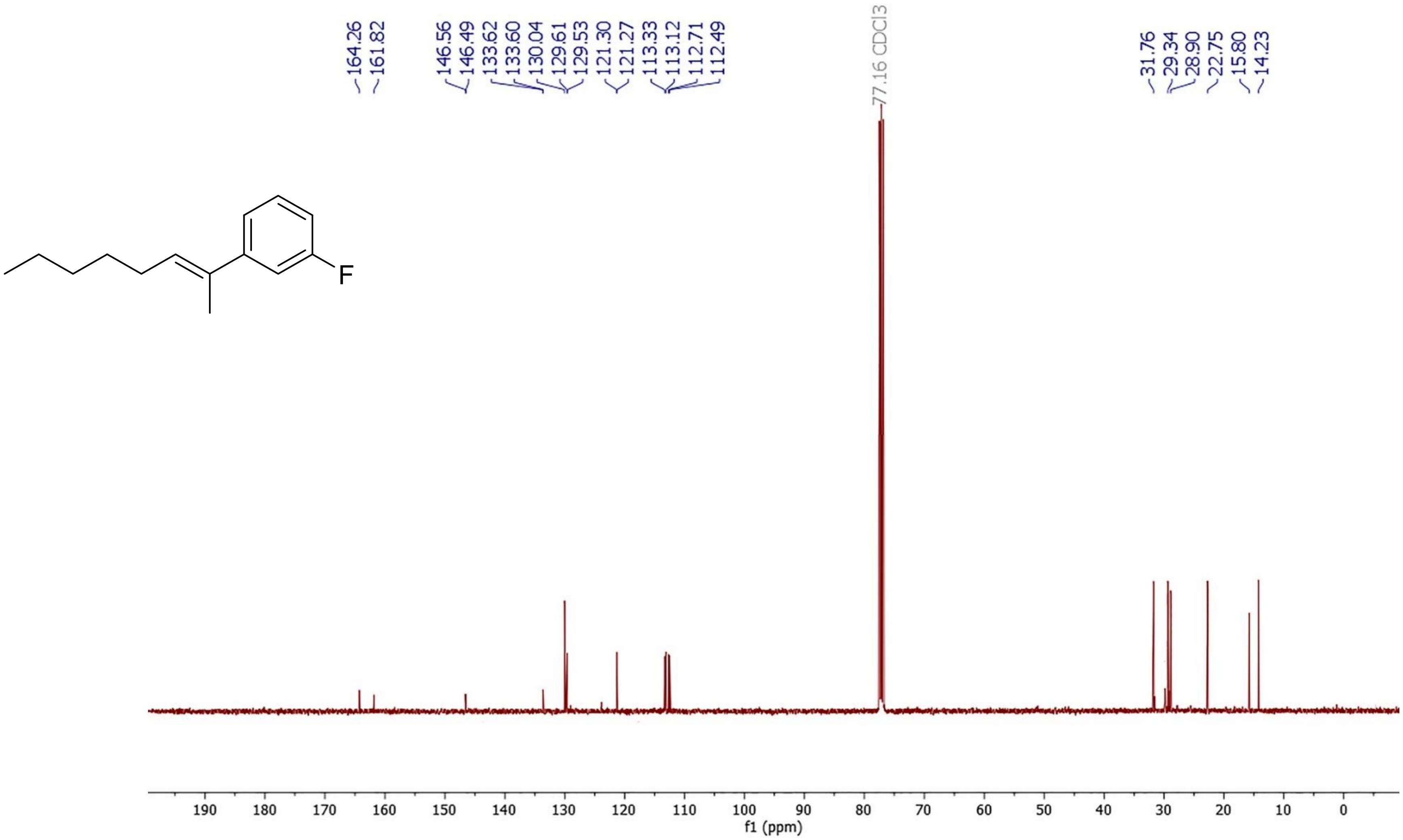

^{19}F-NMR (E)-1-fluoro-3-(oct-2-en-2-yl)benzene (2z)

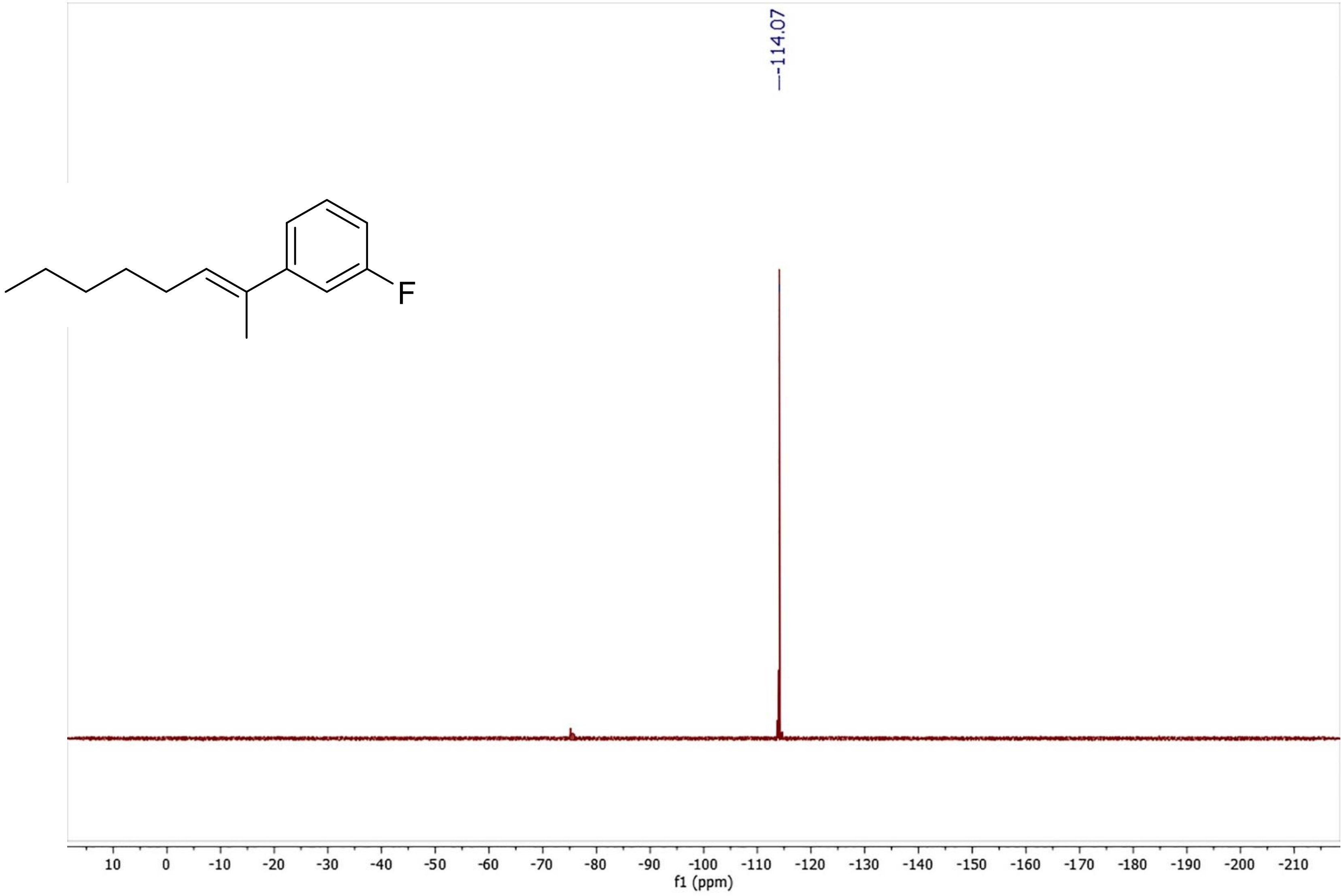

Supplement: Supplementary file 1 — Supporting Information [file EJOC-26-0-s001.pdf]
